# The first specimen of *Camarasaurus* (Dinosauria: Sauropoda) from Montana: The northernmost occurrence of the genus

**DOI:** 10.1371/journal.pone.0177423

**Published:** 2017-05-31

**Authors:** D. Cary Woodruff, John R. Foster

**Affiliations:** 1 Great Plains Dinosaur Museum and Field Station, Malta, Montana, United States of America; 2 Department of Ecology and Evolutionary Biology, University of Toronto, Toronto, Ontario, Canada; 3 Royal Ontario Museum, Toronto, Ontario, Canada; 4 Museum of Moab, Moab, Utah, United States of America; University of Utah, UNITED STATES

## Abstract

A partial skeleton from the Little Snowy Mountains of central Montana is the first referable specimen of the Morrison Formation macronarian sauropod *Camarasaurus*. This specimen also represents the northernmost occurrence of a sauropod in the Morrison. Histological study indicates that, although the specimen is relatively small statured, it is skeletally mature; this further emphasizes that size is not a undeviating proxy to maturity in dinosaurs, and that morphologies associated with an individual’s age and stature may be more nebulous in sauropods.

## Introduction

The macronarian sauropod *Camarasaurus* (represented by the species *C*. *lentus*, *C*. *grandis*, *C*. *supremus*, and *C*. *lewisi*) is far and away the most abundant Morrison Formation (North America; latest Oxfordian–Early Tithonian) sauropod to date (n > 175; [1]). While classically represented at such famous dinosaur localities such as Dinosaur National Monument, Utah, and Como Bluff, Wyoming, the genus is homogeneously distributed throughout the known extent of the formation [2, 3]. Whereas *Camarasaurus* remains have been reported from Montana [4, 1, 3], the Carnegie Museum specimen (CM 1200) is too poorly preserved to be certain of its identity and there is some question as to its formation of origin (M. Lamanna and A. Henrici, pers. comm., 2016); thus, we are unable to confirm these previous reports. The Morrison Formation of Montana is relatively poorly studied and understood compared to most areas within the formation. Montana in fact has more numerous Morrison outcrops and localities than is perhaps commonly perceived. The majority of known localities in the Morrison are restricted to the southernmost portion of the state, and the represented dinosaur genera include the theropod *Allosaurus*, the stegosaurian *Stegosaurus*, and the diplodocoid sauropods *Apatosaurus*, *Diplodocus*, and *Suuwassea* (although this taxon in debated; [5]). Unlike typical Morrison sauropod assemblages, in Montana the sauropod genera are dominated by immature diplodocids with possible *Camarasaurus* material only present in one or two localities (D.C. Woodruff pers. obs.; [1]). This atypical distribution may be attributed to relative under sampling, or perhaps something about the Montana ecosystem was less favorable to the genus. While apparently rare, here we report on the first documented *Camarasaurus* from Montana and one of the northernmost bearing dinosaur localities of the Morrison Formation.

## Materials and methods

**Fig 1:**
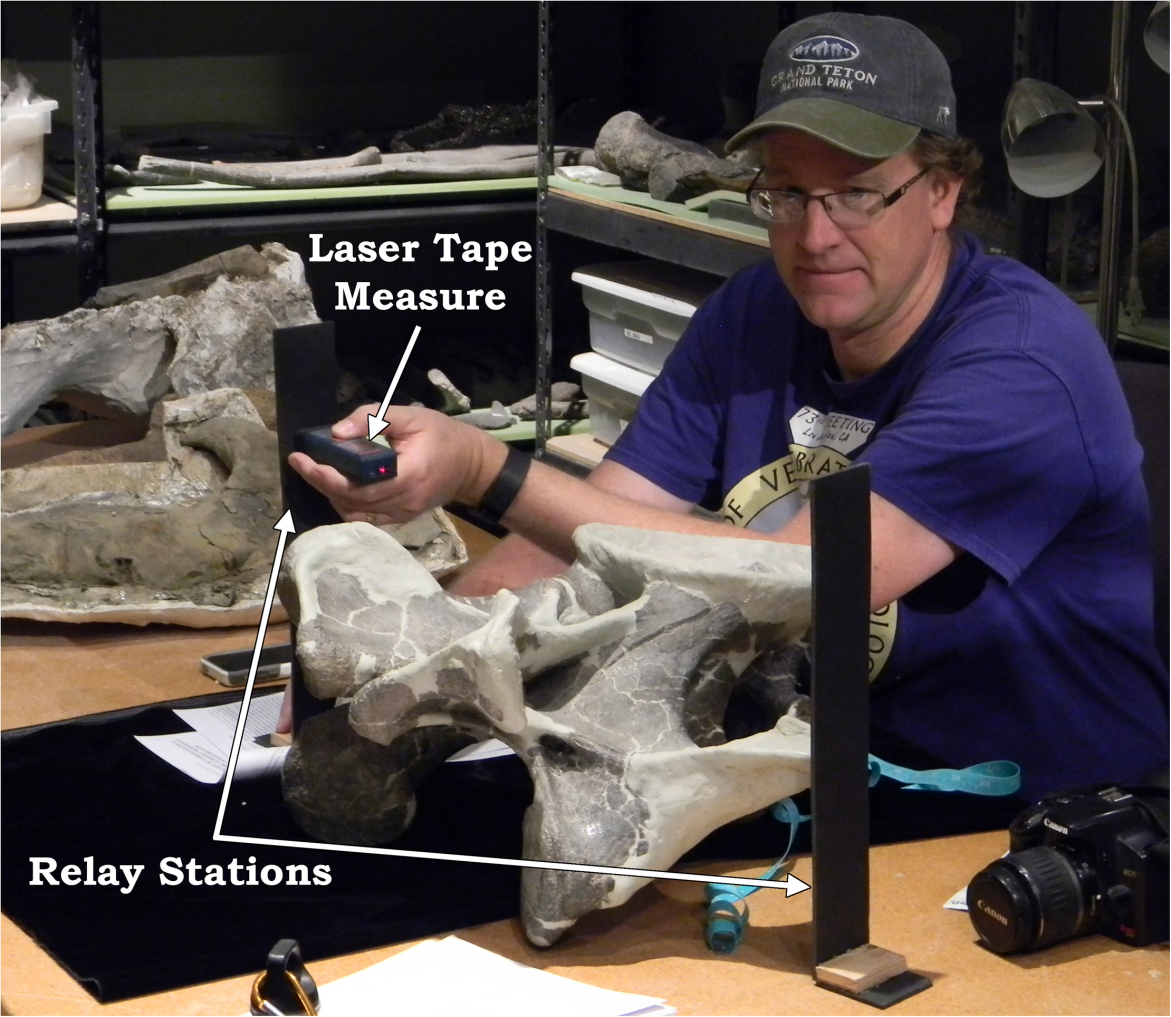
The laser tape measure system used throughout this analysis. J. Foster (pictured) has set the relay stations (denoted) on the opposing ends of a dorsal vertebra’s transverse processes. Placing the laser tape measure (denoted) against the side of one relay station, the beam refracts off of the opposing side, giving a length accurate within 3 mm.

No permits were required for the described study. The specimen was macroscopically examined first hand and documented following standard photographing and measuring techniques. Measurements of skeletal elements utilized a combination of devices from digital calipers, cloth measuring tape, and a Bosch™ GLM 15 Compact Laser Measure. The use of a laser tape measure was a field test and a new addition to aid in the accurate measuring of large elements. Anyone who has ever measured skeletal elements in excess of 1 m is well aware of the difficulties and challenges. Traditionally, measuring large elements required either large calipers or rough approximations with a traditional retracting tape measure. The laser tape measure works by emitting a beam that reflects off the desired end point while deducting the length of the measuring device which results in a total length accurate to within 3 mm (and given that many sauropod elements are in excess of 1 m, a ± 3 mm error is negligible). To measure skeletal elements, two relay stations were constructed which consist of cardstock—or the more durable and preferred Sintra™ PVC foam board—bent at a 90° angle to form an “L”-shaped stand. The relay stations were set up at either end of the desired measurement trajectory, and the measuring device was placed flush against one end and reflected from the opposing station (Fig 1). This digital device allows for far more accuracy than traditional techniques; and with the lightweight relay stations, elements can be measured rapidly, and with sticky wax the stations can even be temporarily adhered to mounted specimens.

Histologic analysis of the specimen was conducted on an anterior thoracic rib and the femur. For the thoracic rib, a section from an anterior rib was sampled distal to the capitulum and tuberculum following the techniques of Waskow and Sander [6]. For the femur, a core section was removed from the anterior face mid-diaphysis following the techniques of Stein and Sander (2009). Preparation and execution of the sectioned elements followed the standard histologic procedures and techniques outlined in Padian and Lamm [7].

### Systematic paleontology

Sauropoda Marsh [8]

Macronaria Wilson and Sereno [9]

*Camarasaurus* Cope [10]

*Camarasaurus* sp.

Here we identify GPDM 220 as belonging to the basal macronarian genus *Camarasaurus*. The overall proportions of the skull–approximately as laterally wide as dorsoventrally tall, the dorsoventrally heightened skull, the vaulted narial region–and the “spatulate” teeth clearly distinguish it as a non-diplodocid. The only other recognized Morrison Formation macronarian is *Brachiosaurus altithorax*. Unlike *Brachiosaurus*, the previously mentioned cranial proportions of GPDM 220 produce a very “box-like” cranial morphology where that of *Brachiosaurus* is more elongate with the strikingly abrupt and dorsally heighted narial region. Furthermore, in examining the vertebrae, GPDM 220 exhibits bifurcated presacral neural spines–which are not present in *Brachiosaurus*, and the cervical vertebrae of *Brachiosaurs* are proportionally far more elongate than any observed in GPDM 220.

**Fig 2:**
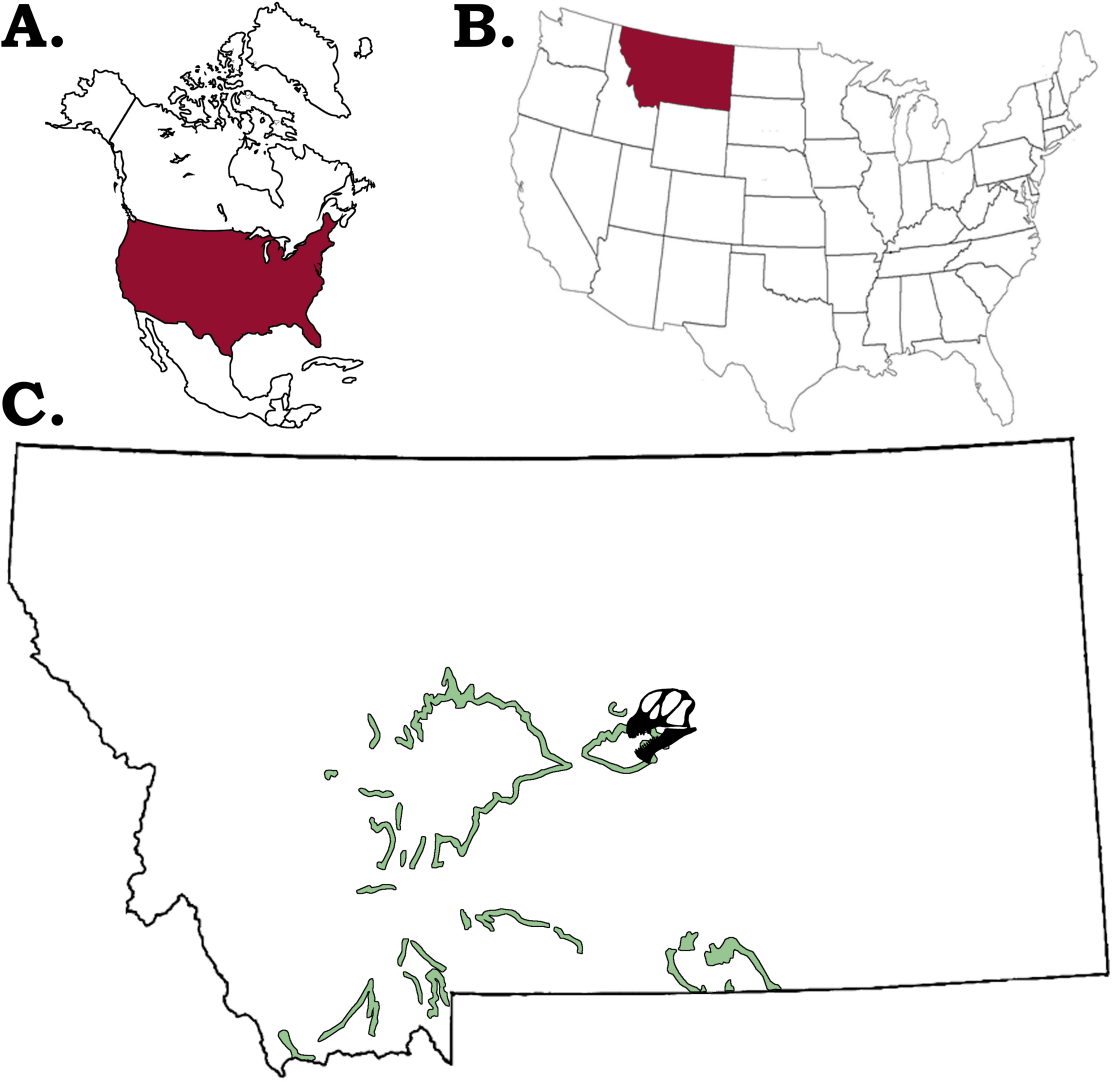
Location of the *Camarasaurus* sp. GPDM 220. A. Map of North America with the United States of America (U.S.A) in burgundy, B. Map of the U.S.A. with the state of Montana in burgundy, C. Map of Montana with the extent of the Morrison Formation in sea green, and the general location of GPDM 220 indicated by the *Camarasaurus* skull silhouette.

Referred Specimen: GPDM 220, partial skeleton consisting of a nearly complete skull and lower jaws, cervicals 1–11, two anterior dorsal vertebrae and a neural arch of a third dorsal, one caudal, a partial left scapula and coracoid, a partial left femur, right tibia, fibula, metatarsal, a single chevron, and several cervical and thoracic ribs (both complete and fragments; Fig 2; all measurements are in Table 1).

**Table 1:** Elemental measurements for the *Camarasaurus* sp. GPDM 220 “Ralph”. All measurements are in mm.

**SKULL**:			
Skull Length -	657 mm		
Skull Width -	313 mm		
Cranial Foramen -	8.57mm long by 3.81 mm wide		
Undistorted Skull Length -	~617 mm		
**DENTARY**:		**LEFT ANGULAR**:	
Left Dentary Length -	359.68 mm	Greatest Anterior-Posterior Length -	285.77 mm
Right Dentry Length -	375.16 mm		
**LEFT PREARTICULAR**:		**LEFT SURANGULAR**:	
Greatest Anterior-Posterior Length -	215.75 mm	Greatest Anterior-Posterior Length -	266.28 mm
		Greatest Proximal-Distal Length -	102.34 mm
**RIGHT SURANGULAR**:			
Greatest Anterior-Posterior Length -	314.33 mm		
Greatest Proximal-Distal Length -	132.05 mm		
**VERTEBRAE**:			
*(All centrum lengths are the total condyle to cotyle length; i*.*e*. *the greatest anterior-posterior length)*			
**Cv1** -		**Cv2** -	
Centrum Length -	57.31 mm	Centrum Length -	183 mm
Centrum Anterior Width -	73.52 mm	Centrum Anterior Width -	80.15 mm
Centrum Anterior Height -	55.05 mm	Centrum Anterior Height -	79.33 mm
Bottom of Centrum To Top Of Neural Spine -	145.30 mm	Dens Height -	30.24 mm
Tip Of Prezyg. To Tip Of Postzyg. -	130.22 mm	Dens Width -	60.67 mm
Centrum Posterior Height -	91.06 mm	Dens Length -	47.60 mm
Centrum Posterior Width -	41.09 mm	Bottom of Centrum To Top Of Neural Spine -	205 mm
		Centrum Posterior Height -	90.60 mm
		Centrum Posterior Width -	80.38 mm
**Cv3** -		**Cv4** -	
Centrum Length -	239 mm	Centrum Length -	292 mm
Centrum Anterior Width -	68.72 mm	Centrum Anterior Width -	61.03 mm
Centrum Anterior Height -	53.76 mm	Centrum Anterior Height -	71.33 mm
Bottom of Centrum To Top Of Neural Spine -	240 mm	Bottom of Centrum To Top Of Neural Spine -	279 mm
Centrum Posterior Height -	100.39 mm	Centrum Posterior Height -	116.33 mm
Centrum Posterior Width -	84.82 mm	Centrum Posterior Width -	90.02 mm
Bifurcation Width -	28.32 mm	Bifurcation Width -	33.68 mm
Bifurcation Depth -	6.42 mm	Bifurcation Depth -	22.70 mm
**Cv5** -		**Cv6** -	
Centrum Length -	332 mm	Centrum Length -	332 mm
Centrum Anterior Width -	108.25 mm	Centrum Anterior Width -	108.25 mm
Centrum Anterior Height -	83.35 mm	Centrum Anterior Height -	86.41 mm
Bottom of Centrum To Top Of Neural Spine -	280 mm	Bottom of Centrum To Top Of Neural Spine -	305 mm
Centrum Posterior Height -	135.68 mm	Centrum Posterior Height -	140.44 mm
Centrum Posterior Width -	101.57 mm	Centrum Posterior Width -	138.19 mm
Bifurcation Width -	51.67 mm	Bifurcation Width -	64.94 mm
Bifurcation Depth -	30.62 mm	Bifurcation Depth -	23.69 mm
		Length Of Pathology -	77.15 mm
		Width Of Pathology -	56.64 mm
		Height Of Pathology -	33.53 mm
		Circumference Of Pathology -	~232 mm
**Cv7** -		**Cv8** -	
Centrum Length -	369 mm	Centrum Length -	373 mm
Centrum Anterior Width -	109.49 mm	Centrum Anterior Width -	93.56 mm
Centrum Anterior Height -	93.08 mm	Centrum Anterior Height -	104.76 mm
Bottom of Centrum To Top Of Neural Spine -	325 mm	Bottom of Centrum To Top Of Neural Spine -	370 mm
Centrum Posterior Height -	156 mm	Centrum Posterior Height -	173 mm
Centrum Posterior Width -	128.25 mm	Centrum Posterior Width -	155.51 mm
Bifurcation Width -	49.42 mm	Bifurcation Width -	43.43 mm
Bifurcation Depth -	26.88 mm	Bifurcation Depth -	37.87 mm
**Cv9** -		**Cv10** -	
Centrum Length -	332 mm	Centrum Length -	304.90 mm
Centrum Anterior Width -	122.19 mm	Centrum Anterior Width -	138.17 mm
Centrum Anterior Height -	135.85 mm	Centrum Anterior Height -	136.03 mm
Bottom of Centrum To Top Of Neural Spine -	405 mm	Bottom of Centrum To Top Of Neural Spine -	335.61 mm
Centrum Posterior Height -	194 mm	Centrum Posterior Height -	169.36 mm
Centrum Posterior Width -	156 mm	Centrum Posterior Width -	154.04 mm
Bifurcation Width -	37.37 mm	Bifurcation Width -	26.81 mm
Bifurcation Depth -	49.26 mm	Bifurcation Depth -	20.85 mm
**Cv11** -			
Centrum Length -	282.28 mm		
Centrum Anterior Width -	143.99 mm		
Centrum Anterior Height -	140.82 mm		
Bottom of Centrum To Top Of Neural Spine -	289.87 mm		
Centrum Posterior Height -	172.15 mm		
Centrum Posterior Width -	150.63mm		
Bifurcation Width -	39.66 mm		
Bifurcation Depth -	21.52 mm		
**D1** -		**D2** -	
Centrum Length -		Centrum Length -	197 mm
Centrum Anterior Width -		Centrum Anterior Width -	227 mm
Centrum Anterior Height -		Centrum Anterior Height -	183 mm
Bottom of Centrum To Top Of Neural Spine -		Bottom of Centrum To Top Of Neural Spine -	464 mm
Centrum Posterior Height -		Centrum Posterior Height -	198 mm
Centrum Posterior Width -		Centrum Posterior Width -	199 mm
Bifurcation Width -		Bifurcation Width -	46.90 mm
Bifurcation Depth -		Bifurcation Depth -	57.77 mm
**Caudal** -			
Centrum Length -	172 mm		
Centrum Anterior Width -	133.76 mm		
Centrum Anterior Height -	123.42 mm		
Bottom of Centrum To Top Of Neural Spine -	274 mm (reconstructed)		
Centrum Posterior Height -	119.94 mm		
Centrum Posterior Width -	136.66 mm		
**SCAPULA**:		**FEMUR**:	
Greatest Anterior-Posterior Length -	779.22mm	Greatest Proximal-Distal Length -	1097.15 mm
Greatest Proximal-Distal Length -	359.56 mm	Diaphysis Circumference -	509 mm
(*posterior to glenoid*)			
**TIBIA**:		**FIBULA**:	
Greatest Proximal-Distal Length -	655 mm	Greatest Proximal-Distal Length -	642 mm
Proximal Width -	230 mm	Proximal Width -	121.46 mm
Distal Width -	238 mm	Distal Width -	138.59 mm
Diaphysis Circumference -	376 mm	Diaphysis Circumference -	224 mm
**METACARPAL**:			
Greatest Proximal-Distal Length -	178.64 mm		
Proximal Width -	74.27 mm		
Distal Width -	54.85 mm		
**DORSAL RIBS**: (*complete ribs*)			
**Dr5** -		**Dr7** -	
Greatest Proximal-Distal Length -	1002.24 mm	Greatest Proximal-Distal Length -	1755.16 mm
Width of Rib -	277.13 mm	Width of Rib -	243.50 mm
(*lateral to medial*)		(*lateral to medial*)	

Age and Locality: From the undivided Morrison Formation in the Little Snowy Mountains within Fergus County, Montana; Late Jurassic. Associated fauna includes partial *Stegosaurus* and two theropod teeth.

## Description

**Fig 3:**
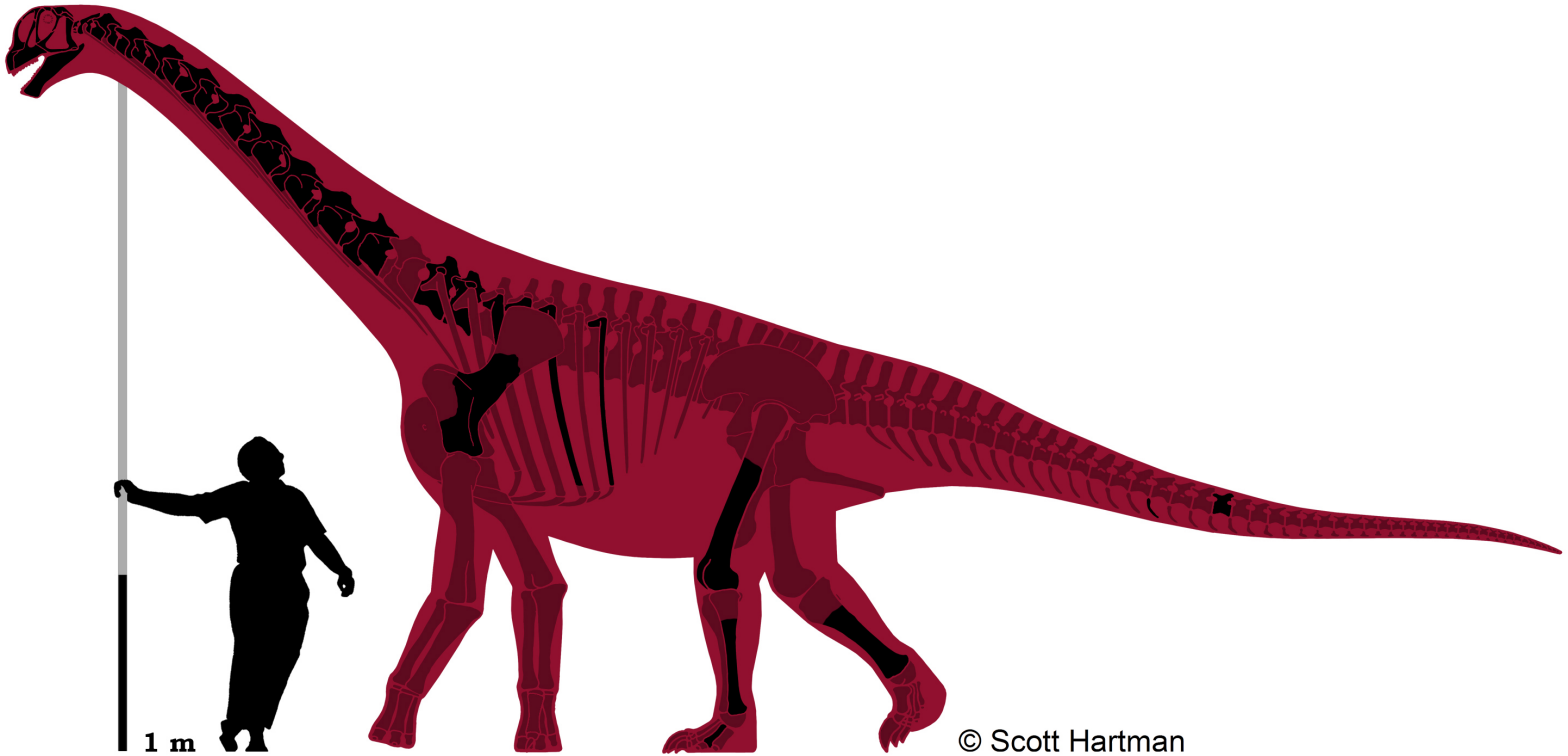
Skeletal reconstruction of the *Camarasaurus* sp. GPDM 220. Black elements represent material known from GPDM 220, burgundy elements are missing. Scale bar = 1 m, silhouette is of esteemed and renowned sauropod expert John “Jack” Stanton McIntosh. Skeletal reconstruction of *Camarasaurus* by S. Hartman.

This specimen, affectionately referred to as “Ralph” in honor of the original farming homesteader of the property–was collected in 2005 by N. Murphy and is from the Little Snowy Mountains region within Fergus County, Montana (Fig 3). While the remains were originally collected by the Judith River Dinosaur Institute, since 2003 the specimen has been under the scientific protection of the Judith River Foundation and the Great Plains Dinosaur Museum.

### Cranial

#### Skull

**Fig 4:**
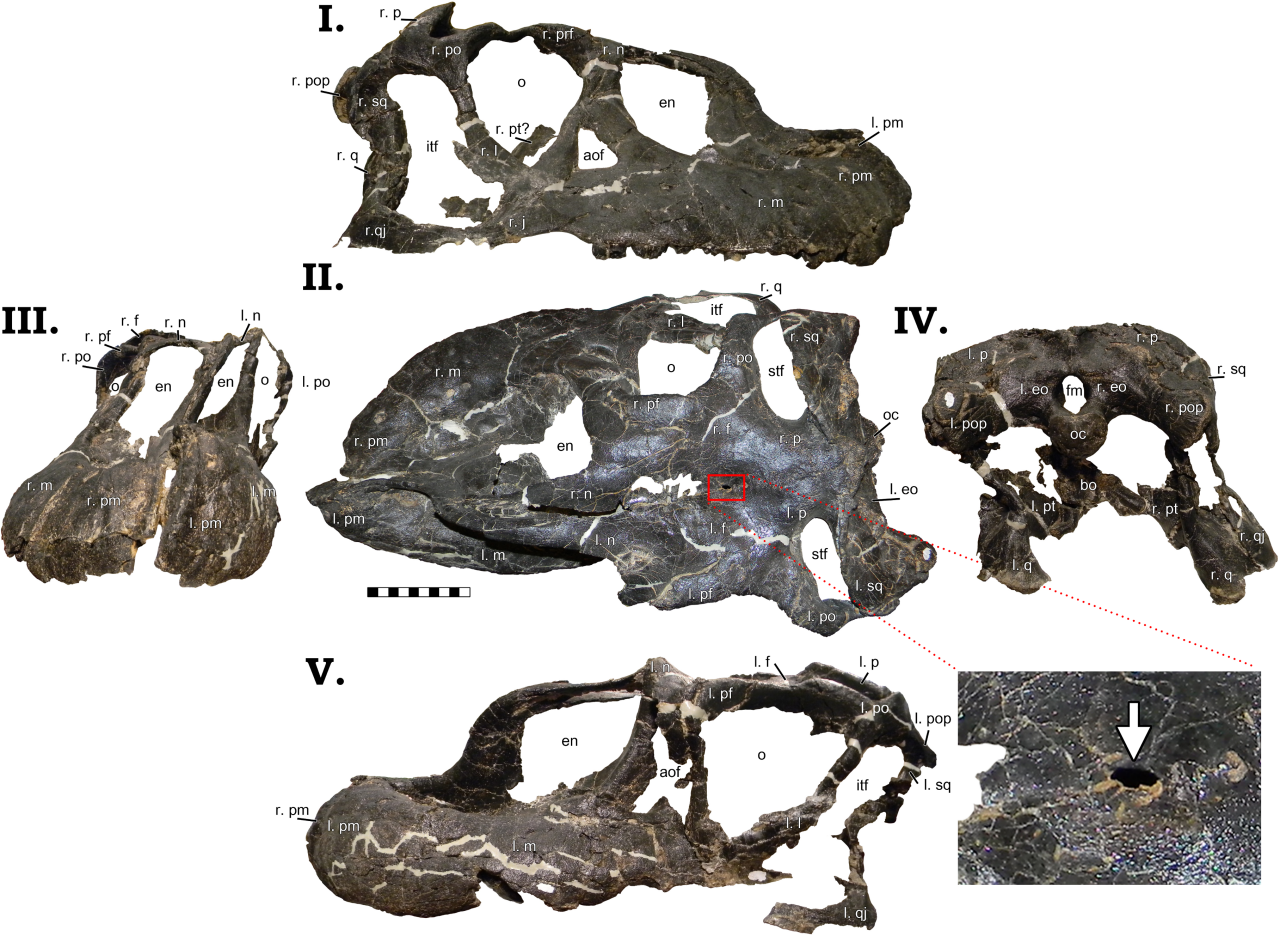
**Skull of GPDM 220 in right lateral [I], dorsal [II], anterior [III] posterior [IV], and left lateral [V] views.** Note the red inset box in the dorsal view (II) which highlights the postparietal aperture (indicated by the white arrow). All orientations to scale. Scale bar = 10 cm. Abbreviations: l. left, r. right, aof antorbital fenestra, bo basioccipital, eo exoccipital, en external naris, f frontal, fm foramen magnum, itf infratemporal fenestra, j jugal, l lacrimal, m maxilla, n nasal, o orbit, oc occipital condyle, p parietal, pm premaxilla, po postorbital, pt pterygoid, pop paraoccipital process, prf prefrontal, sp squamosal, stf supratemporal fenestra, q quadrate, qj quadratojugal.

The most striking portion of GPDM 220 is its skull. Essentially complete, it has nevertheless suffered some taphonomic distortion (some shearing and dorsoventral crushing); yet for a skull of its size, it is in remarkable condition (Fig 4). Left and right lateral, dorsal, anterior, and posterior views of the skull are freely accessible and completely unobscured. The ventral aspect could theoretically be examined–currently the skull is secured in a ventral cradle, so alternative cradles could be constructed to allow access. However, such was not feasible during this analysis, so the ventral aspect could not be examined. Following the practice of McIntosh et al. [11], cranial elements will be described collectively–not separately–unless unique morphology or special circumstance requires such.

As in GMNH-PV 101, the premaxillae of GPDM 220 are large and robust (to quote McIntosh et al. [11]–“massive”). The main portion of the premaxilla is rather rectangular in shape–dorsoventrally taller than anteroposteriorly elongate–which is seen in the other *Camarasaurus* skulls GMNH-PV 101, CM 1138, DNM 28, DNM 13786, SDSM 114501, AMNH 467, DNM 975 [12]. While small foramina are present, they are dispersed over the distal portions of the premaxillae, and none in particular are notably sizeable (unlike the four large and distinct foramina of GMNH-PV 101). Due to the dorsoventral crushing, the exact nature of the dorsal and ventral premaxillary processes is not discernable. However, both lateral orientations have clear views of the nasal processes. The nasal process is dosoventrally elongate, and tapers and projects posteriorly. The right nasal process is dorsoventrally crushed, giving the dorsal border of the naris a sub-horizontal border. It is important to note that dorsal process of the premaxilla forms a nearly 90° with the top edge of the main portion of the premaxilla. This angled process is not observed in other *Camarasaurus* specimens; however the “unique” morphology of this process is due to dorsoventral taphonomic distortion (and such corroborating distortion is observed in the left maxilla as well).While the premaxillary teeth are not complete (and a ventral view is obstructed by the protective cradle), both premaxillae appear to have had four teeth each.

The posterior portions of both nasals are crushed, but they appear to broaden posteriorly towards their contact with the frontals. The left lateral margin is crushed and distorted, but in right lateral view this contact is intact. It could be due to taphonomic distortion of the lacrimal and the nasal process of the premaxilla, but in right lateral view, the posterior and ventrally projecting process of the nasal appears to be laterally wider and extends more posteriorly (not a quick tapering) than in other *Camarasaurus* specimens. Thus, as in all other *Camarasaurus* specimens, the enlarged naris is ringed by the premaxilla, naris, and maxilla. The naris of GPDM 220 initially appears sub-circular–not an elongated ovoid as observed in other *Camarasaurus* specimens. Again we would stress that this is nothing more than a taphonomic artifact, and that in life the nares were elongate and ovoid.

The left maxilla is highly distorted, but the right maxilla is exquisitely preserved. In lateral profile, the maxilla is anteroposteriorly elongate. In some *Camarasaurus* specimens (such as DNM 28, DNM 13786, and SMA 0002), the maxilla is anteroposteriorly elongate (more rectangular) than others (such as YPM 1905, CM 11338, and DNM 975) which are a bit more dorsoventrally tall (more of a square shaped overall profile). The anterior most portion of the maxilla (pre-naris) is anteroposteriorly elongate and fairly uniform in dorsoventral thickness. The nasal process of the maxilla is stout—~3 cm in width–and is posteriorly inclined ~ 40° to the long axis of the maxilla. None of the maxillary teeth are complete, but it appears that the right maxilla has nine teeth (the posterior region of the left maxilla is incomplete, so a dental formula cannot be determined). An interesting attribute to the maxilla (and only visible on the complete right maxilla) is a strong ventral curvature (“notch”) in the posterior region. Immediately after the posterior most maxillary tooth, the maxilla has a strong deflection. Many *Camarasaurus* specimens exhibit this posterior deflection, but in most, this is more of a gentle step (GMNH-PV 101, YPM 1905, DNM 28, CM 11338, DNM 13786, and AMNH 467). However, the *Camarasaurus* specimens SMA 0002 and DNM 975 have a similar posterior maxillary curvature to GPDM 220.

Both lacrimals are slightly distorted, but otherwise fairly complete. The lacrimal has a wide posterior border (contact with the maxilla and jugal), and only slightly tapers anteriorly. An anteriorly tapered lacrimal seems to be evenly divided amongst *Camarasaurus* (present in CM 11338, DNM 13786, DNM 975, and SMA 0002; while absent in YPM 1905, DNM 28, AMNH 467, GMNH-PV 101, and GPDM 220), thus this “gracile” and “robust” morphology may simply be intraspecific variation. Interestingly, the lacrimals of GPDM 220 lack the laterally directed spur along the dorsolateral margin which in observed in other *Camarasaurus* specimens. The antorbital fenestra of all *Camarasaurus* specimens exhibits a widened ventral border and tapers anteriorly, producing an elongated (“teardrop”-shaped) antorbital fenestra. In GPDM 220, the antorbital fenestra exhibits this widened ventral and tapered dorsal margins, yet dorsoventrally it appears to be much shorter and instead triangular in profile. As with much of the cranial profiles, we believe that the dorsoventral taphonomic distortion explains this apparent antorbital fenestra morphology anomaly. In life, we believe that the antorbital fenestra of GPDM 220 was the typical *Camarasaurus* “teardrop” shape.

Both prefrontals are distorted, but they bear the typical *Camarasaurus* morphology of being anteroposteriorly short with a widened medial flange. The postorbital of GPDM 220 is one of the more interesting cranial elements. Superficially it bears the *Camarasaurus* typical morphology–an anteriorly elongate portion that contacts the frontal, a posteriorly squat portion that contacts the squamosal, and an anteriorly curved and elongate ventral process. Yet the posteriorly squat portion that contacts the squamosal is unlike that from any *Camarasaurus* thus described (including GMNH-PV 101). This posterior process in other *Camarasaurus* tapers quickly to either a flat or slightly pointed squamosal contact. In GPDM 220, this posterior process is shorter than the anterior process, but its dorsoventral width is much greater than previously observed. As opposed to posteriorly tapering, this process retains a fairly uniform width and is square-shaped. This particular morphology does not seem to be a taphonomic artifact, and thus we can only surmise it is an intraspecific attribute. The orbit of GPDM 220 in profile is, as McIntosh et al. [11] describe, an “inverted teardrop”. Dorsoventral distortion produces a more flattened dorsal border, but again this would not be the morphology in life. The jugal is distorted, but present on both sides. The anterior portion has expanded maxillary and quadratojugal contacts, and posteriorly, it appears to taper along the postorbital. The right quadratojugal of GPDM 220 is intact, and it has a wide ventral curvature as expressed in other *Camarasaurus* specimens. The right squamosal is the more intact of the two, but it is still highly distorted. The squamosal would appear to have a more anteroposteriorly widened proximal end; and though crushed, it appears to have had a slight sigmoidal-like curvature–as in other *Camarasaurus*.

In dorsal view, the nasals have a larger–more square-shaped–posterior portion. As observed in CM 11338, DMN 28, and GMNH-PV 101, the posterior mostportion of the nasals in GPDM 220 possesses a small posteriorly oriented projection which forms part of the articular contact with the frontal. In dorsal view the frontals constitute the largest portion of the skull roof. In dorsal view, while there is some taphonomic distortion, the frontal express the *Camarasaurus* typical frontal morphology that is rather rectangular–laterally wider than anteroposteriorly elongate. Approximately 7 cm from the posterior portion of the frontal is a small foramen (8.57 mm long by 3.81 mm wide; Fig 4). Other *Camarasaurus* specimens appear to possess this foramen–AMNH 467, AMNH 973, YPM 1905, DMN 28, and UUVP 3568 –while this feature is absent in others–CM 11338, CM 11969, DNM 975, and USNM 13786; and in other preserved skulls this region is highly damaged, thus this foramen may be obliterated. This foramen is located at the sutural contact of articulating frontals, and it is indeed a true opening, not a depression. In diplodocids, there is a similar opening (albeit generally larger) historically known as the “postparietal foramen” (and this feature has even proposed to be a pineal eye; [13]).

In *Camarasaurus*, this feature has been noted historically. In 1879, O.C. Marsh was the first to hypothesize this feature being a pineal eye in *C*. *grandis* [14]. In 1958 T.E. White noted this feature (and dubbed it the pineal foramen; [15]) in several *Camarasaurus* skulls. As noted by Madsen et al. [12], the prevalence of this cranial feature led White [15] to erroneously believe that this was a synapomorphy of the genus. In their extremely thorough review of *Camarasaurus* cranial material, Madsen et al. [12] proposed four theories to explain this feature: 1) the opening is ontogenetic, 2) the feature is a taphonomic artifact, 3) the feature is a vestige of a pineal opening sporadically expressed amongst *Camarasaurus*, or 4) the distribution of this feature indicates two *Camarasaurus* species. Citing the lack of this opening in the smallest skull to date–CM 11338 –Madsen et al. [12] do not believe it to be ontogenetic; and while this feature does appear among different *Camarasaurus* species, Madsen et al. [12] believes the distribution is more likely a result of taphonomy.

The absence of a cranial opening earlier in development does not falsify the possibility of the foramen being an ontogenetic development–the parietal fenestration in *Triceratops* happens late in ontogeny [16]. At this time, a histologic examination has not been conducted to test the ontogenetic distribution. Likewise, an examination of all *Camarasaurus* cranial material has not been conducted, thus we cannot accurately assess the distribution or taphonomic potential. Thus for the time being, we cannot falsify an ontogenetic or taphonomic explanation. In light of this uncertainty, we suggest that for the interim, this feature not be identified as a pineal foramen. Pineal suggests photoreception, and if this feature is ontogenetic, it cannot be a foramen. Therefore we recommend for the time being following a neutral phrasing–thus identifying this feature as the frontal aperture.

The parietals are a bit sheared (the right being the more intact of the two), and as in other *Camarasaurus* specimens (such as GMNH-PV 101) the exact sutural contacts between the parietals is not clearly evident. The posterior portion of the parietal represents the largest portion of this element, and it is strongly rectangular in overall profile (nearly three times wider laterally than they are rostrocaudally). The supratemporal fenestrae of GPDM 220 are very large–nearly 20% of the total cranial length, which appears to deviate from the “typical” *Camarasaurus* morphology (such as CM 11338, UUVP 4286, or UUVP 3568). As with the parietals, the supratemporal fenestrae are strongly laterally elongate (approximately three times wider laterally than they are rostrocaudally). The supratemporal fenestrae are rimmed by the frontals, parietals, and postorbitals.

In posterior view, the skull is sheared some degrees left laterally (see Fig 4 IV). The most striking feature in posterior view is the large foramen magnum. As expressed in other *Camarasaurus* specimens (AMNH 5761, CM 11338, DNM 28, GMNH-PV 101, SMA 0002, UUVP 10070, UUVP 4286, UUVP 3568, YPM 1905), the foramen magnum is oval in profile–dorsoventrally taller than laterally wide. Due to the high prevalence of this ovoid profile, we would suggest that the morphology of the foramen magnum may be synapmorphic in *Camarasaurus*, and likewise potentially diagnostic amongst Morrison sauropod taxa.

At the midline of the parietals is a slight sagittal nuchal crest, which is observed in some other *Camarasaurus* specimens and some immature diplodocids. The occipital condyle is rather circular with a flattened dorsal surface. From this dorsal surface to the foramen magnum is a gentle sulcus. As in other *Camarasaurus* specimens (AMNH 5761, CM 11338, DNM 28, GMNH-PV 101, SMA 0002, UUVP 10070, UUVP 4286, UUVP 3568, YPM 1905), the paroccipital processes are proportionally large and robust. As in these other specimens, the paroccipital processes have a prominent ventrolateral projection. The end of the processes are strongly flared, with a gentle to sub-flat lateral curvature. The quadrates are vertically oriented, and exhibit the *Camarasaurus* typical widened and robust distal ends. More of the delicate posterior cranial process (such as the basipterygoid processes and the stapes) are highly distorted or obscured by matrix, thus their exact morphology is undeterminable at this current time.

#### Lower jaw

**Fig 5:**
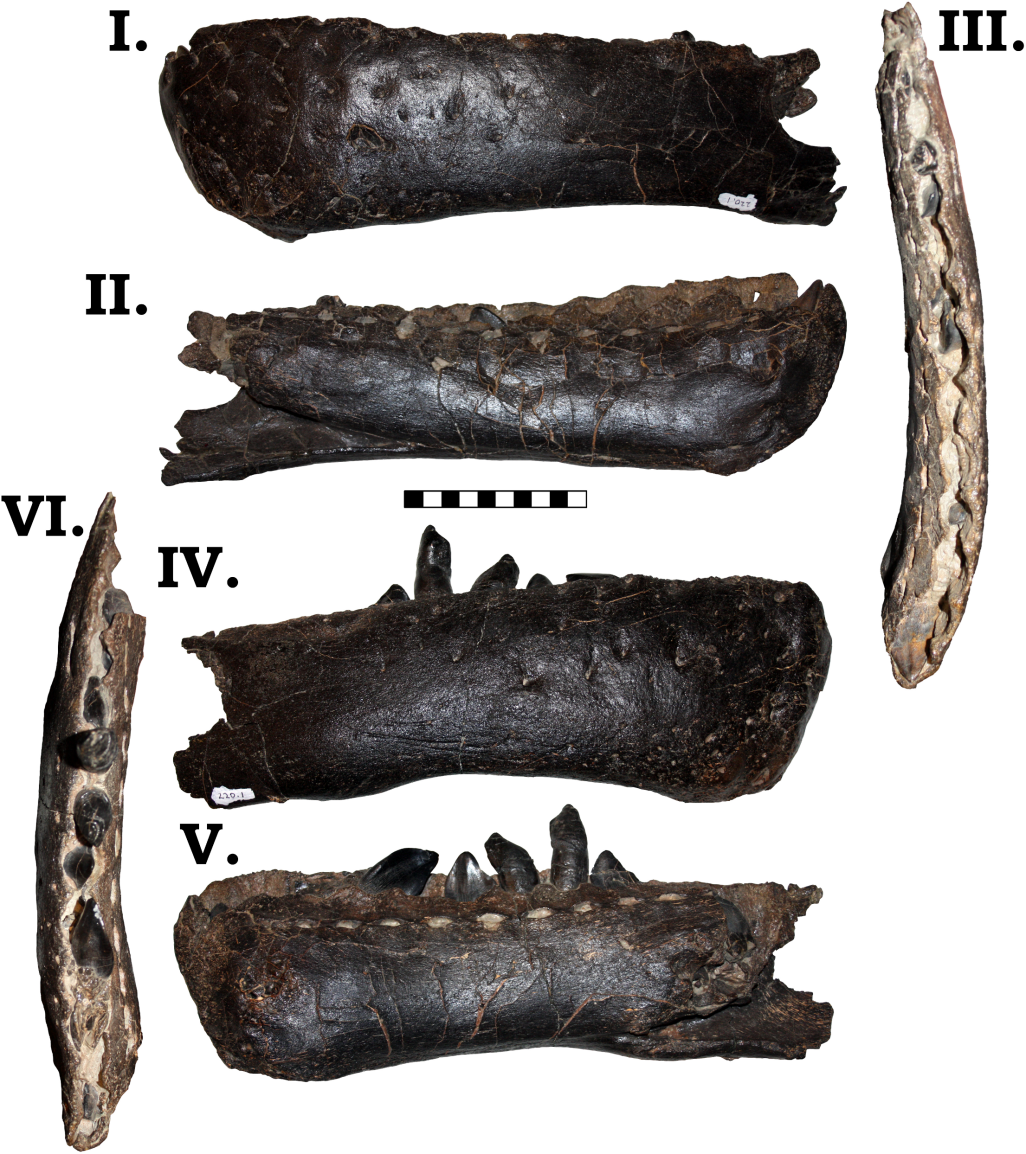
**Left (I–III) and right (IV–VI) dentary of GPDM 220 in lateral (I & IV), medial (II & V), and dorsal (III & VI) views.** All orientations to scale. Scale bar = 10 cm.

Both left and right dentaries are preserved in GPDM 220 (Fig 5). The preserved portion of the left dentary is slightly longer anteroposteriorly than the right. Both dentaries have teeth *in situ* (the right dentary has more teeth in place); and based on alveoli, the left dentary had a total of 14 teeth, while the right dentary had 13. Most *Camarasaurus* dentaries appear to have 11–14 alveoli, and a difference between left and right in the same individual is not unprecedented [3]. GPDM 220 displays the projecting anterior prominence at the dentary symphysis (a.k.a. a “chin”), so often attributed to *Camarasaurus* (however a “chin” is likewise observed in *Mamenchisaurus* and some turiasaurs). However, within *Camarasaurus*, there is quite a degree of variability in the prominence of this “chin”. In GPDM 220, GMNH-PV 101, CM 11338, DNM 28, SDSM 114501, and SMA 0002, this ‘chin” is not as prominent as in AMNH 467, DMN 957, DNM 13786, UUVP 3609, YPM 1905. The developmental presence/absence of this projecting “chin” could be ontogenetic–absent in the small CM 11338, present in the large DMN 13786; however, in consideration of the varied size distribution of this feature, we believe it more parsimonious to explain this distribution as intraspecific variation (alternatively distribution through size could be used to advocate for dimorphism).

**Fig 6:**
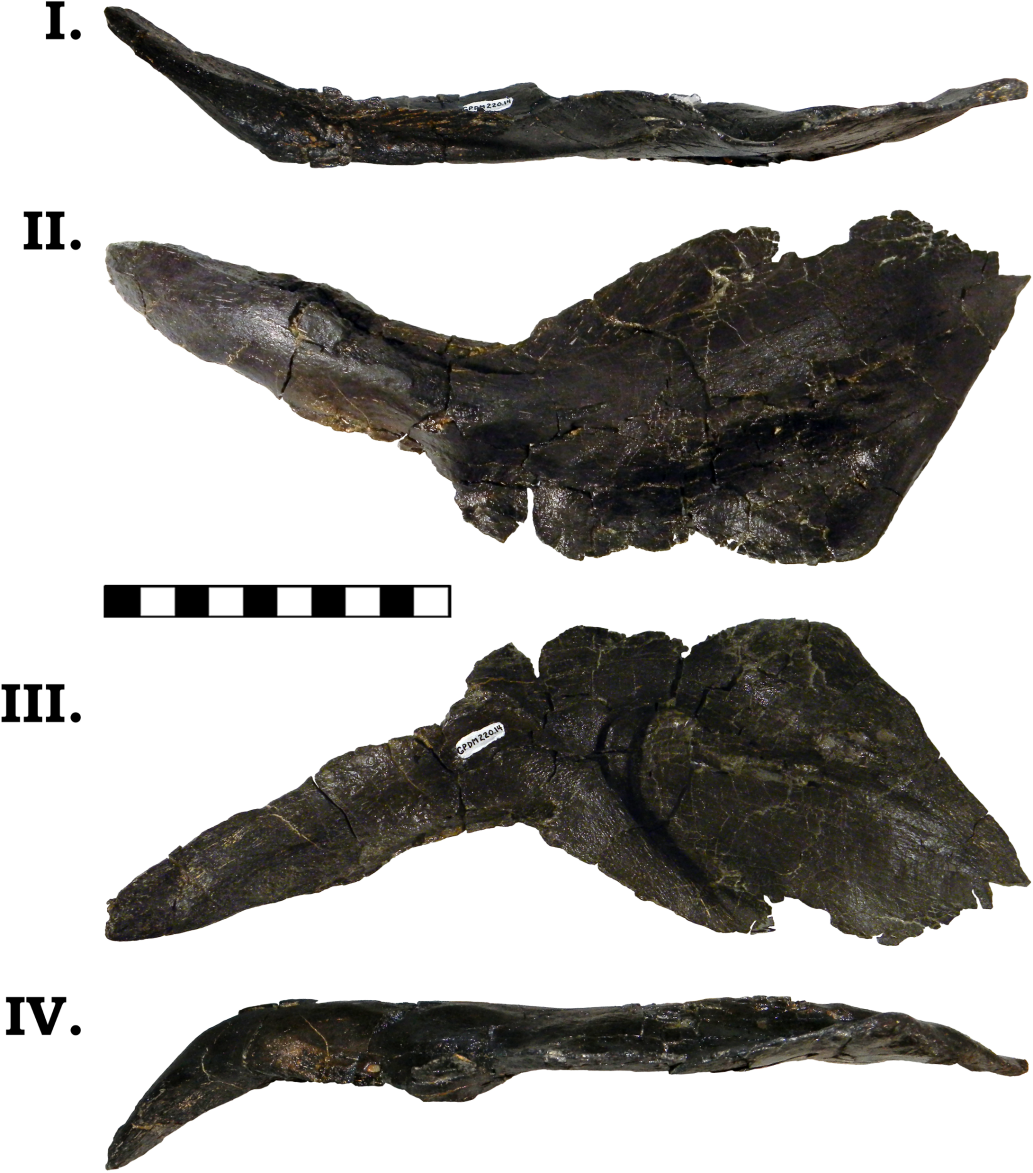
**Left surangular of GPDM 220 in dorsal (I), lateral (II), medial (III), and ventral (IV) views.** All orientations to scale. Scale bar = 10 cm.

**Fig 7:**
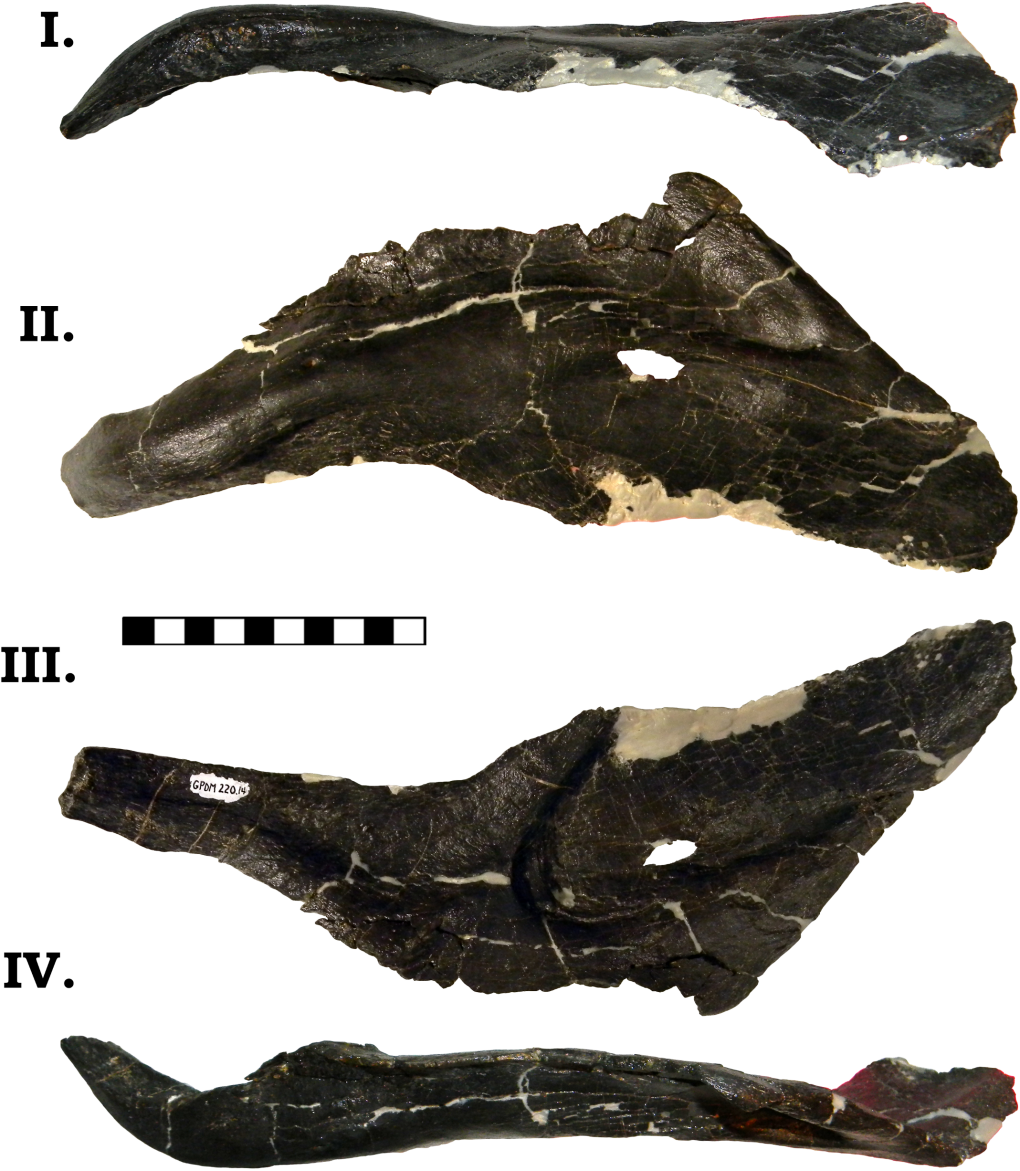
**Right surangular of GPDM 220 in dorsal (I), lateral (II), medial (III), and ventral (IV) views.** All orientations to scale. Scale bar = 10 cm.

**Fig 8:**
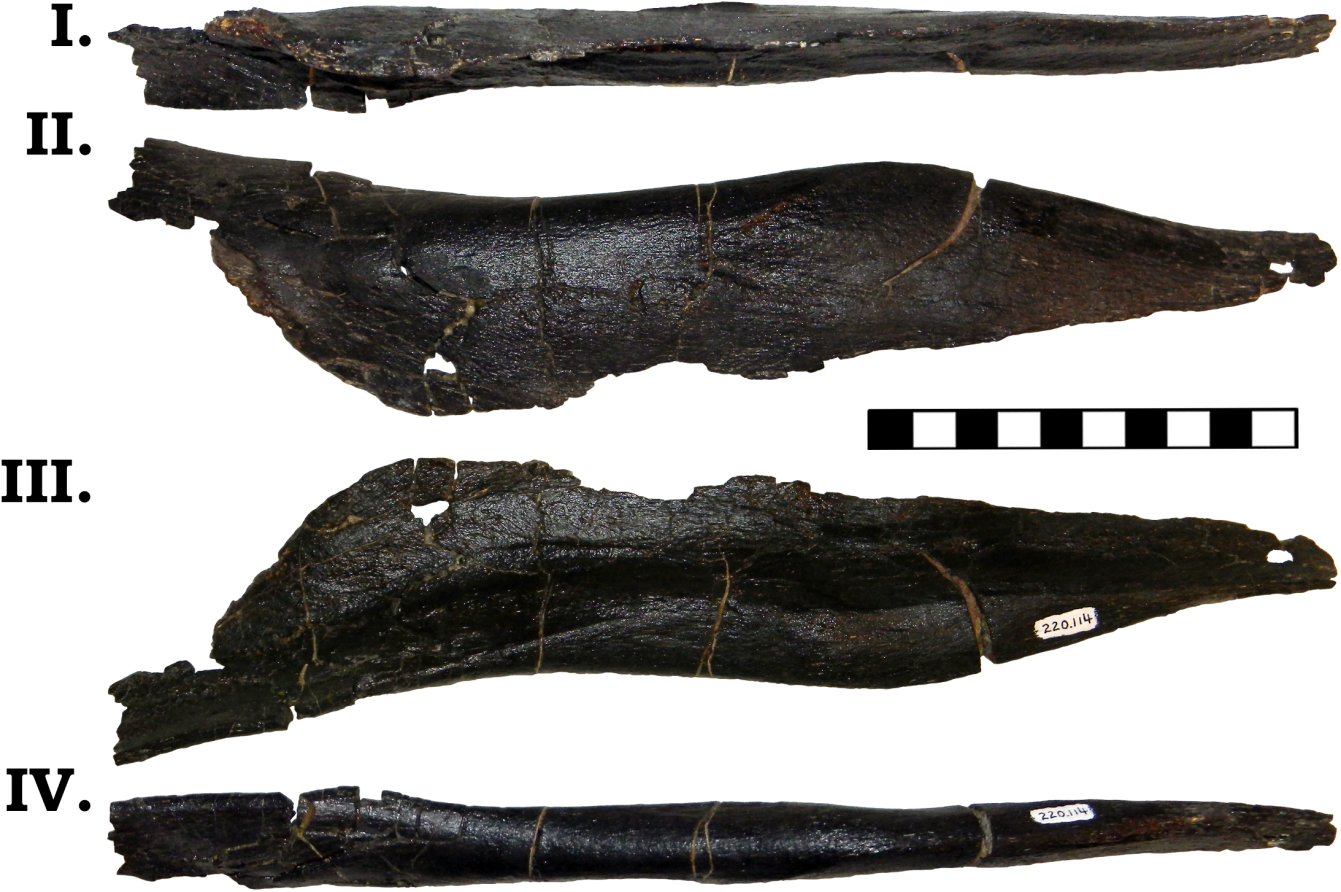
**Left angular of GPDM 220 in dorsal (I), lateral (II), medial (III), and ventral (IV) views.** All orientations to scale. Scale bar = 10 cm.

**Fig 9:**
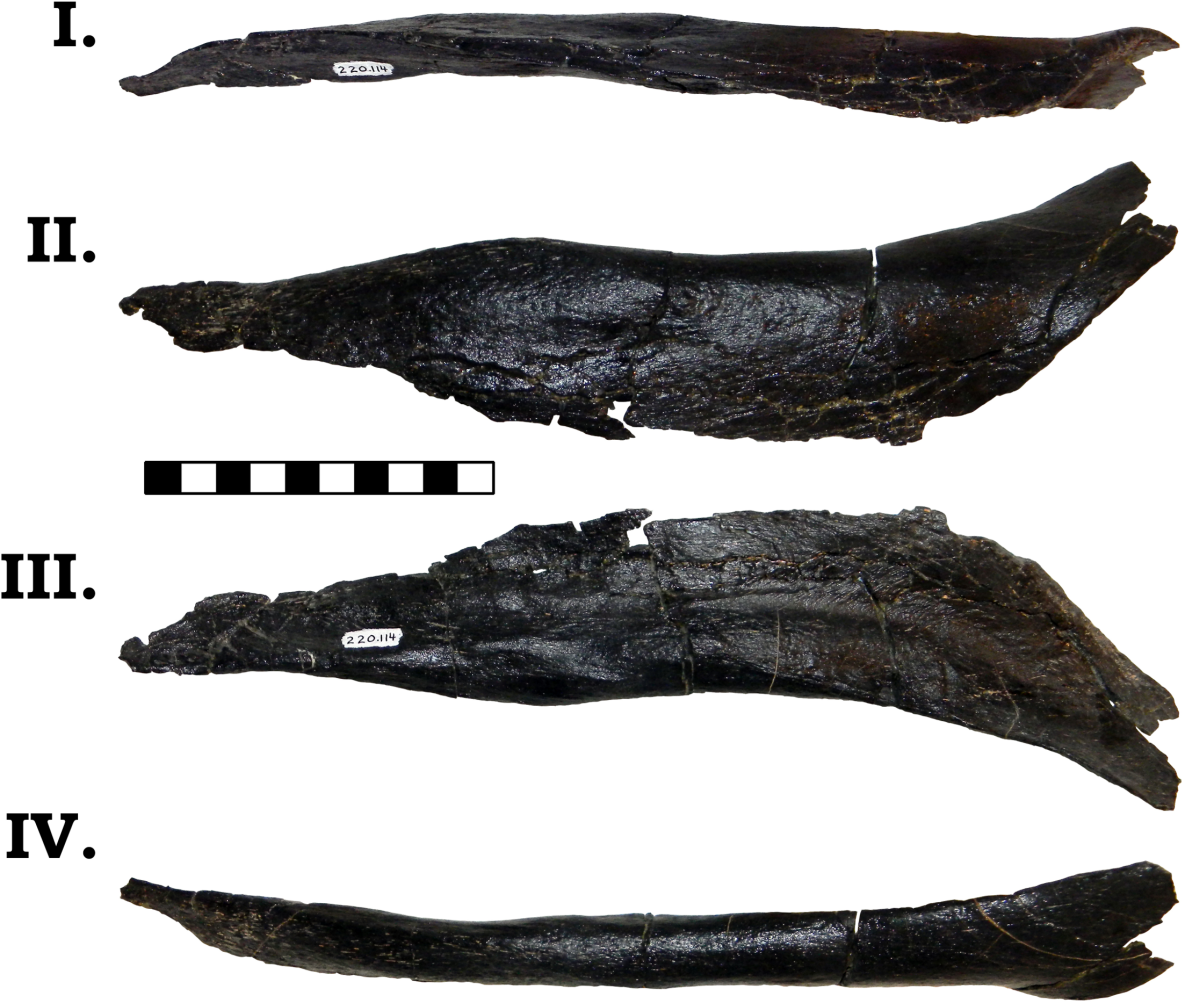
**Right angular of GPDM 220 in dorsal (I), lateral (II), medial (III), and ventral (IV) views.** All orientations to scale. Scale bar = 10 cm.

**Fig 10:**
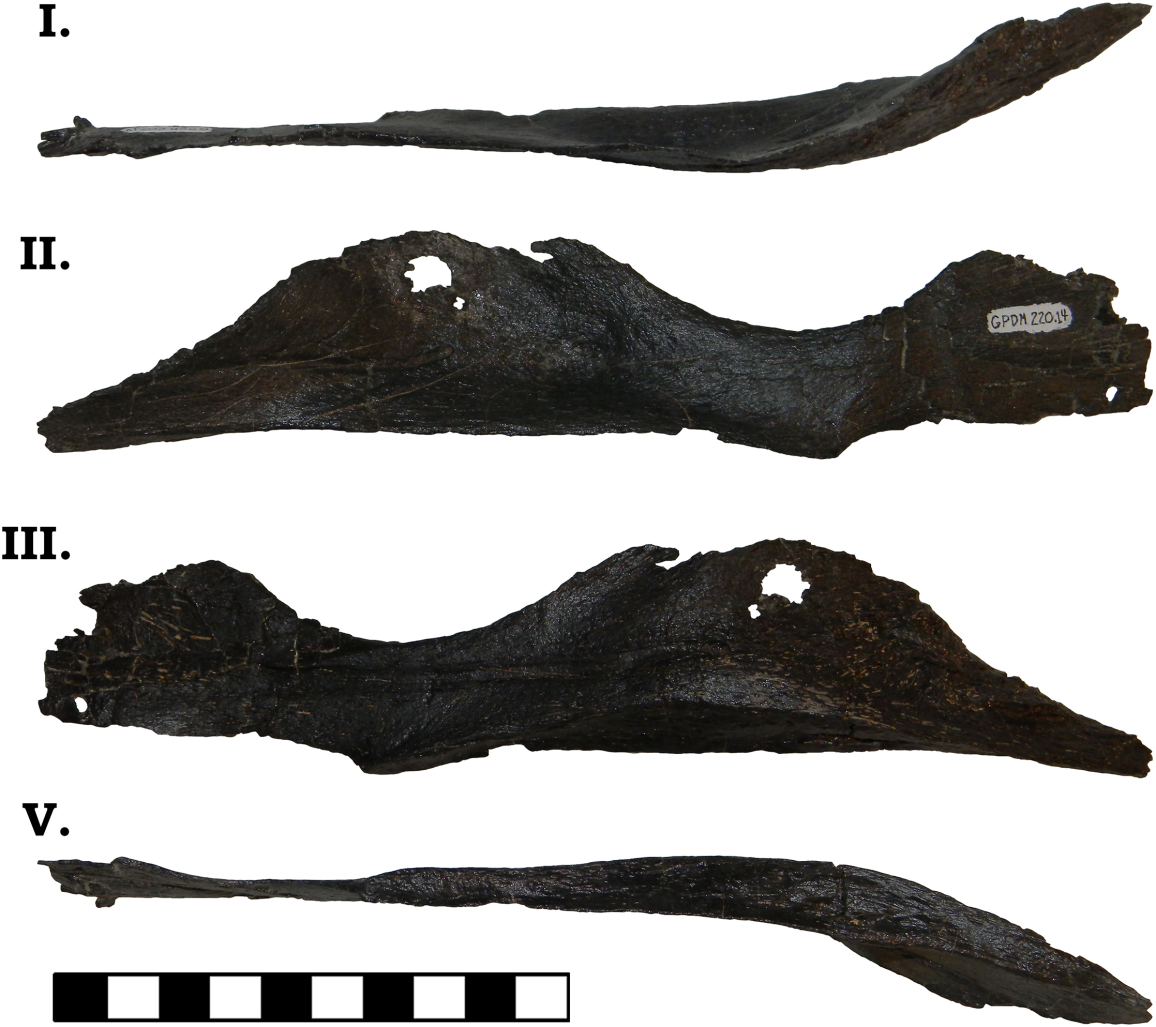
**Left prearticular of GPDM 220 in dorsal (I), lateral (II), medial (III), and ventral (IV) views.** All orientations to scale. Scale bar = 10 cm.

The posterior jaw elements consist of the surangular, angular, and the prearticular. The surangular is represented by both halves (Figs 6 and 7). The surangular of GPDM 220 is strongly similar to that of GMNH-PV 101. As described by McIntosh et al. [11], the surangular is “hatchet”-shaped–the anterior portion is greatly dorsoventrally expanded, with a sudden, dosoventrally thinned posterior “handle”. The angular is represented by both halves, and is largely complete–minus broken delicate anterior and posterior mostmargins (Figs 8 and 9). The angular is anteroposteriorly elongate and strongly anteriorly tapering. Unlike the angular of GMNH-PV 101 that display a weak to gently “sigmoidal” curvature, the angular of GPDM 220 exhibits a greater “sigmoidal” profile more reminiscent of the *Camarasaurus* sp. UUVP 10068. Represented by only the left half, the prearticular of GPDM 220 is very similar to that of *C*. *lentus* DMN 975 (Fig 10). The prearticular is anteroposteriorly elongate and very delicate. The splenial articulating end is dorsoventrally widened, while the articular connection is shortened and abruptly tapered.

### Post-cranial

#### Axial. Vertebrae

**Fig 11:**
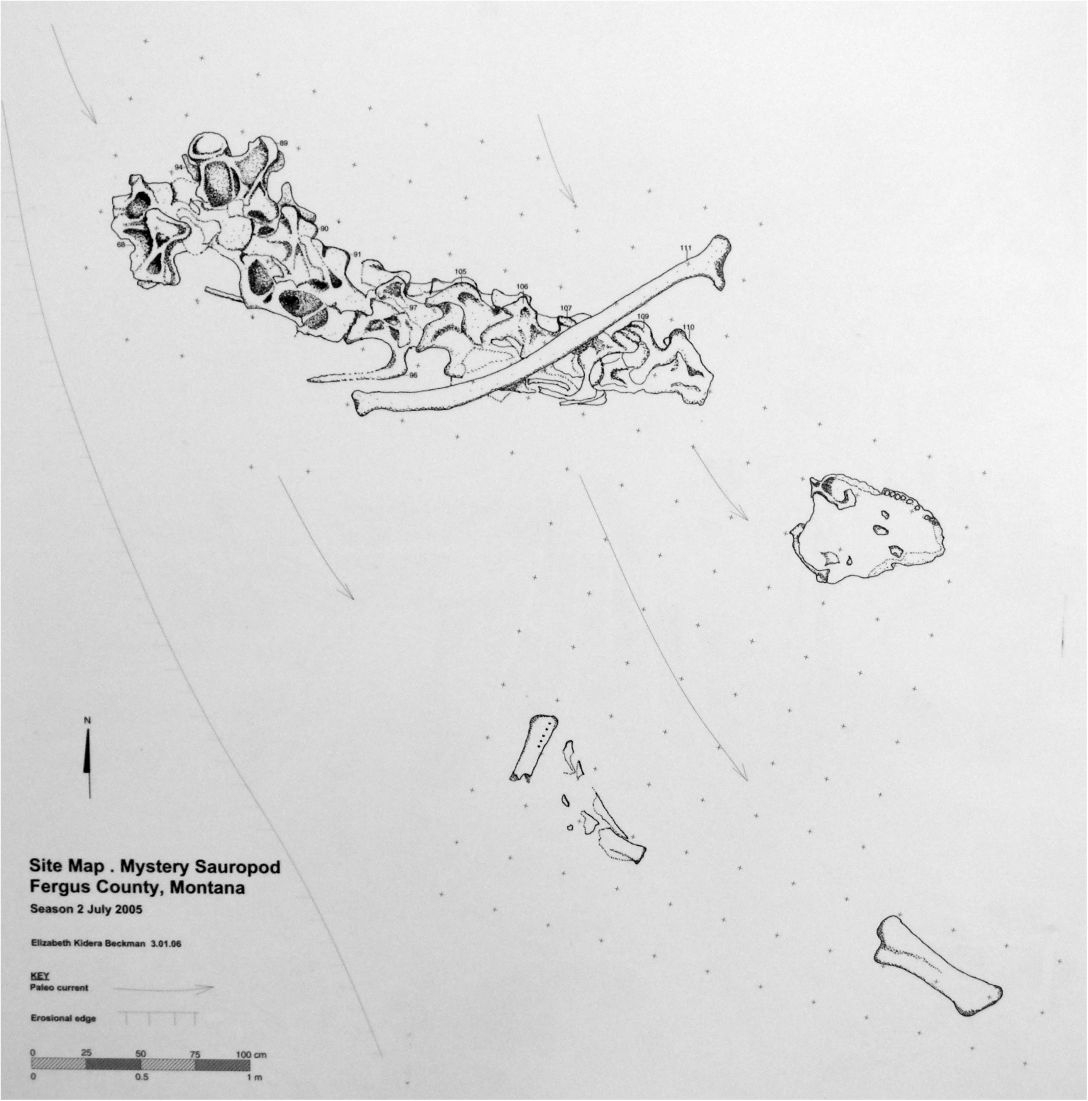
Quarry map of GPDM 220. Note for an unknown reason, only a portion of the referred elements are portrayed on the map. Map created by Elizabeth Kidera Beckman.

The vertebral column of GPDM 220 is represented by a partial articulated cervical series (Cv1-Cv11), two dorsals, and a single caudal. It is important to note that the quarry map for GPDM 220 only illustrates ten (possibly eleven) vertebrae (Fig 11). These vertebrae appear to represent the largely articulated cervical series (the posterior most vertebrae shown are associated but out of articulation). Aside from this vertebral series, the only other elements represented on the quarry map are a single dorsal rib, the skull, a dentary (and presumably associated lower jaw elements), and the tibia. None of the other skeletal elements are documented, thus their proximity and exact degree of association to the mapped material is completely unknown. While the cervical count is an important feature in *Camarasaurus* (AMNH 5761 –*C*. *supremus* and SMA 0002—*C*. *lewisi* both have thirteen, while other *Camarasaurus* specimens have twelve), the morphology of the cervicals present indicates that only eleven are represented. While the two represented dorsals are identified as D1 and D2 (see below), we cannot offer a grounded explanation as to why C12 (nor any of the other dorsals) is not present.

#### Cervical vertebrae. Cv1

**Fig 12:**
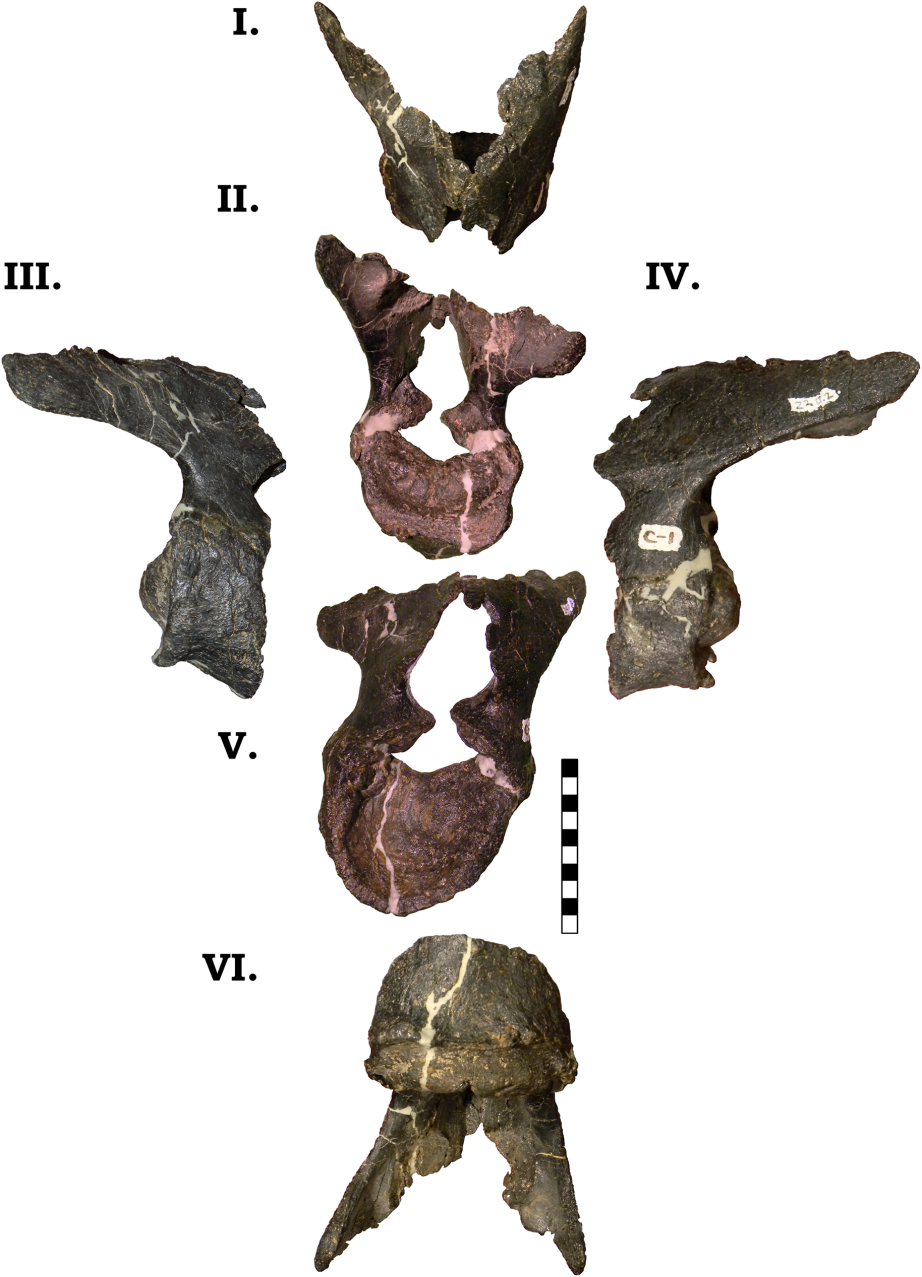
**Cervical vertebra 1 of GPDM 220 in the six anatomical planes (dorsal [I], anterior [II], right [III] and left lateral [IV], posterior [V], and ventral [VI]).** All orientations to scale. Scale bar = 10 cm.

The atlas closely resemble that of the *Camarasaurus grandis* (GMNH-PV 101; Fig 12). The procoelous anteroposteriorly short centrum possesses a cotyle that has a pronounced “lower lip” [11], and likewise the neurapophyses appear to be firmly fused, yet the neurocentral suture is still highly evident. It is interesting to note that in all of the other vertebrae, the neural arches are completely fused, and neurocentral sutures are largely non-evident. As in GMNH-PV 101, the posteriorly elongate neurapophyses are directed dorsally and laterally. In GMNH-PV 101, McIntosh et al. [11] describes a circular “cup-like” opening ventral to the neural canal that accommodates the odontoid process. The margins of this circular opening are formed by two “jaw-like” processes that do not join [11]. However, in GPDM 220, these processes nearly join, which results in a nearly closed, “keyhole” type opening. Two distinct facets are observed on the ventral surface of the cotyle, which McIntosh et al. [11] proposed was the articulation site for cervical ribs (a unique character previously only observed in GMNH-PV 101; [11]). No material or fragments are present that we believe to be remnants of the atlas ribs.

#### Cv2

**Fig 13:**
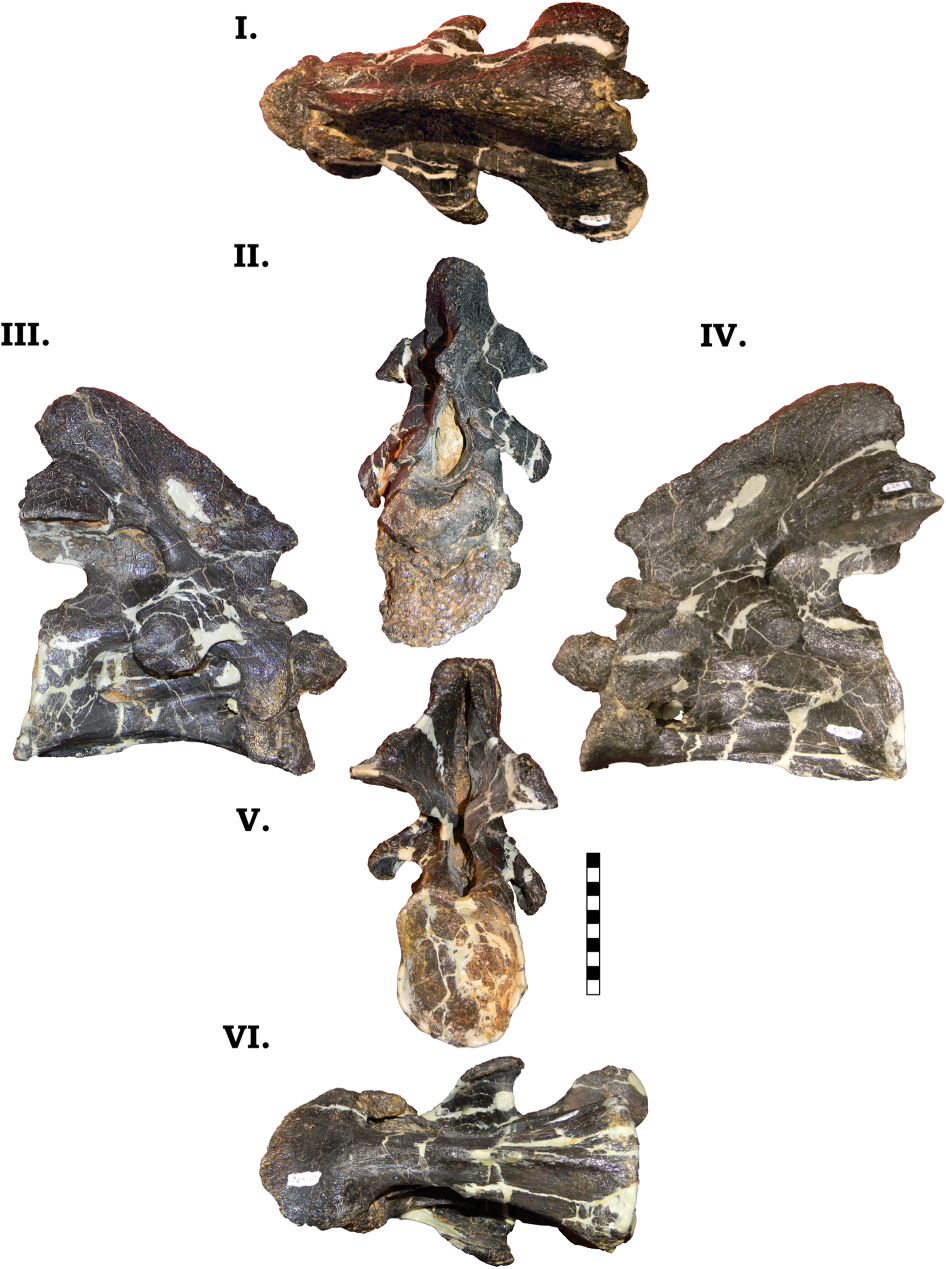
**Cervical vertebra 2 of GPDM 220 in the six anatomical planes (dorsal [I], anterior [II], right [III] and left lateral [IV], posterior [V], and ventral [VI]).** All orientations to scale. Scale bar = 10 cm.

The axis has a single, un-divided, large, anteroposteriorly ovoid pneumatic foramen that appears procamerate to weakly camerate (Fig 13). The robust odontoid process, is more “square”-shaped than that observed in GMNH-PV 101 (approximately as anteroposteriorly long as dorsoventrally tall), yet both specimens exhibit an equally prominent anteriorly angled superior articular facet. The neural arch (approximately 2/3 the height of the centrum) terminates with a bulbous spine apex. In anterior view, the neural spine is approximately 2/3 the width of the centrum, yet in both GMNH-PV 101 and BYU 9047, the inflated neural spine apex is approximately 1:1. In lateral view, the entire neural arch appears to be shorter in overall length and slightly more robust than GMNH-PV 101 (a trait shared with BYU 9047). The postzygapophyses of GPDM 220 appear to be more horizontally inclined than in GMNH-PV 101, and this slight difference could possibly be explained as an artifact of taphonomic distortion. Interestingly, in GMNH-PV 101 the posteriorly inclined neural arch extends approximately ¼ past the cotyle, yet in GPDM 220, the posteriorly angled neural spine does not project past the cotyle. The robust diapophyses and parapophyses indicates a bicapitate cervical rib, yet the cervical ribs were not fused (note numerous isolated cervical ribs are known from GPDM 220, however as of this analysis attempts to pair them to the cervical series were notconducted). As in GMNH-PV 101, GPDM 220 Cv2 possesses a very prominent and strong ventral keel. The keel increases in prominence posteriorly, and at its zenith projects nearly 1 cm ventrally. As in GMNH-PV 101, the ventral keel in GPDM 220 exhibits a more “plateaued” posterior portion.

An unusual feature beginning in this cervical of GPDM 220 is a paired opening in the postzygapophyses. Within the cervical vertebrae that exhibit this feature, it is predominantly present on the ventral side of the postzygapophyses. In Cv2 of GPDM 220, this feature is present only on the left postzygapophysis. This feature is located laterally on the postzygapophyseal facet, and the larger of the two openings is more medial. In total these openings occupy an area ~1 square cm. These openings are anteroposteriorly elongate, yet there are no grooves, scratches, or marks that are more typically associated with feeding traces [17, 18], nor are there bulges or cratering in conjunction with the openings as associated with lesions [19].

#### Cv3

**Fig 14:**
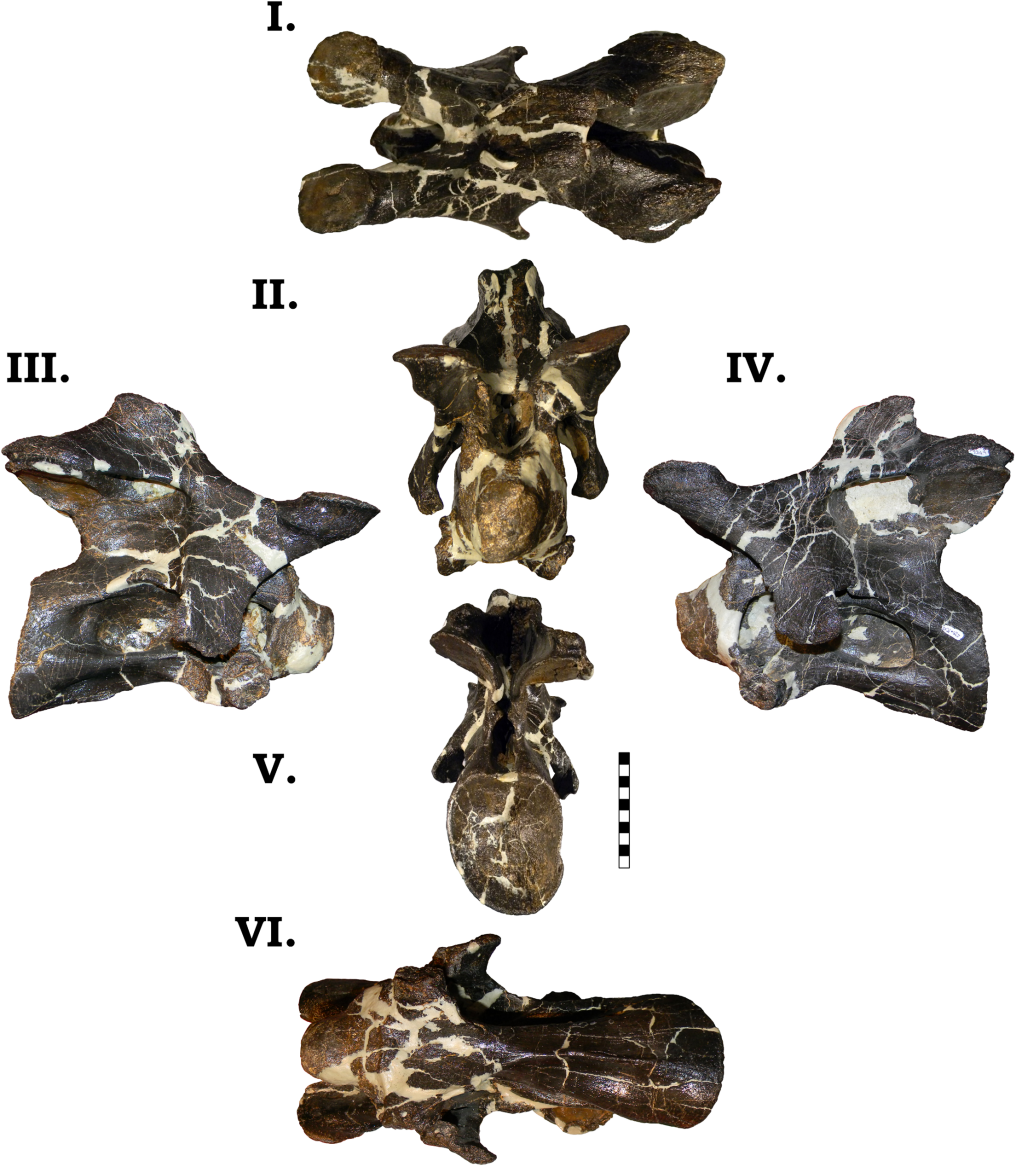
**Cervical vertebra 3 of GPDM 220 in the six anatomical planes (dorsal [I], anterior [II], right [III] and left lateral [IV], posterior [V], and ventral [VI]).** All orientations to scale. Scale bar = 10 cm.

In the third cervical, the large, anteroposteriorly ovoid pneumatic foramen is camerate (Fig 14). The lateral profile of this centrum is different than that of GMNH-PV 101 or BYU 9047. In these specimens, the centrum has a strong ventral “kink”, and the ventral margin of the centrum is very sinuous. In GPDM 220 the centrum lacks this strong “kink”–resulting in a straighter (anteroposterior) centrum. In GMNH-PV 101 the spherical and very prominent condyle possesses a slight ventral posteriorly projecting hook; while the condyle of GPDM 220 is more gracile and lacks this ventral hook. The cotyle is ovoid (dorsoventrally taller than laterally wide), opposed to the very circular cotyle of GMNH-PV 101. As in the preceding vertebra (and in Cv3 of GMNH-PV 101) there is a strong ventral keel. As in Cv2, the cervical ribs are not fused to the diapophyses and parapophyses. The neural arch of GPDM 220 is more “square” shape (dorsoventrally shorter: ~2/3 the height of the centrum; and dorsoposteriorly short: sub equal to the centrum height) and the posterior portion of the neural arch is sub equal to the cotlye–opposed to the more dorsoposteriorly elongate–“rectangular”–neural arch of GMNH-PV 101. As in both GMNH-PV 101 and BYU 9047, the apex of the neural spine in GPDM 220 is asymmetrically incipiently bifurcated. The trough of bifurcation is laterally wider than dorsoventrally deep–producing a shallow and “U”-shaped trough. As in Cv2, Cv3 of GPDM 220 possess the paired zygapophyseal openings. However, the paired openings in Cv3 are only on the left prezygapophyses.

#### Cv4

**Fig 15:**
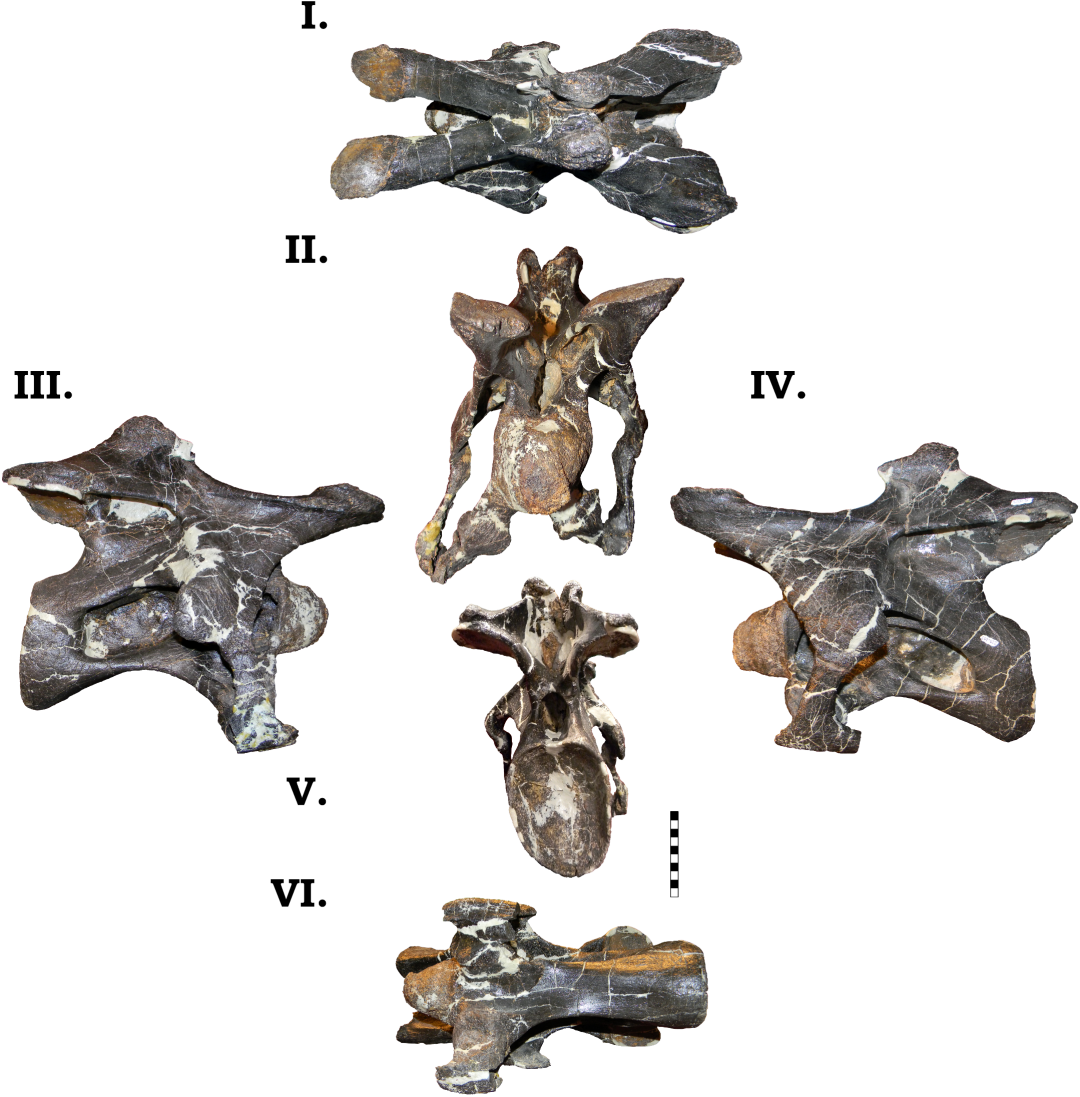
**Cervical vertebra 4 of GPDM 220 in the six anatomical planes (dorsal [I], anterior [II], right [III] and left lateral [IV], posterior [V], and ventral [VI]).** All orientations to scale. Scale bar = 10 cm.

In the strongly opisthocoelous centrum, the large camerate pneumatic fossae are slightly angled dorsally, which is similarly observed in GMNH-PV 101 (Fig 15). In lateral profile the centrum has less of a ventral “kink” than the preceding vertebra. The strongly projecting condyle is slightly dorsoventrally taller than anteroposteriorly long, and the overall lateral shape of the condyle tapers anteriorly (unlike the circular and “squat” condyle of GMNH-PV 101). As in GMNH-PV 101, in GPDM 220, in lateral view, moving ventrally from the cervical ribs to the end of the cotyle, the ventral side of the centrum lacks the strong ventral keel previously encountered. Instead, there is a strong ventral curvature to the end of the cotyle. As opposed to the strongly circular cotyle of GMNH-PV 101, the cotlye of GPDM 220 is dorsoventrally ovoid (however, note that this vertebra does exhibit shearing, so the strongly ovoid cotyle could be taphonomic distortion). In ventral orientation the overall shape differs from that of GMNH-PV 101. In GMNH-PV 101 the ventral morphology is reminiscent of an I-Beam–the condyle and cotyle are laterally wide, and progressing towards the middle, the centrum width greatly tapers (analogous to the “transversely wide” morphotype of Ikejiri [20]). The distance from the anteriormost portion of the condyle to the posterior portion of the parapophyses is approximately 2/3 the length from the posterior portion of the parapophyses to the posterior most portion of the cotyle. Yet in GPDM 220, the ventral aspect of the centrum does not have the I-Beam profile, and the condyle to parapophyses length is shorter than the parapophyses to cotyle length.

As in GMNH-PV 101, the ventral portions of the parapophyses where the capitula of the cervical ribs attach are robust, and in Cv4 the cervical ribs are fused to the vertebra. However, in GPDM 220 these parapopheseal contacts are incredibly bulbous. In fact, these portions are so enlarged, that if they were not paired, one could be easily taken for a pathology. These bulbous articular ends are approximately half the width of the condyle, and are nearly as laterally wide as the parapophyses are dorsoventrally long. Interestingly, these bulbous articular ends are not likewise reflected in the form of enlarged capitular facets. In lateral profile, the prezygapophyses are very prominent, and project anteriorly past the condyle. The prezygapophyseal facets are laterally and anteroposteriorly enlarged, and anteriorly they are slightly angled (as in GMNH-PV 101). The neural arch of GPDM 220 is anteroposteriorly elongate (as in GMNH-PV 101), except the postzygapophyses posteriorly extend past the cotyle (opposed to that of GMNH-PV 101). As in GMNH-PV 101, in lateral view, the posterior portion of the diapophyses posteriorly project; yet in GPDM 220 the projections are very prominent–almost “wing-like” (approximately equal in dorsoventral height to the condyle). In regards to the neural spine, the spine of GPDM 220 and GMNH-PV 101 are very different. The neural spine apex of GMNH-PV 101 is very “isosceles”- like in lateral view (strongly dorsally peaked with the anterior and posterior slopes and length being near equal), while the neural spine apex of GPDM 220 is anteroposteriorly elongate. Posteriorly from the prezygapophyses, heading “up” the spine, there is a strong anteriorly oriented projection. Dorsally after this projection, the apex of the spine plateaus for approximately 3 cm. Unlike the corresponding spine of GMNH-PV 101, yet observed in BYU 9047, the spine apex of GPDM 220 is bifurcated. The trough of bifurcation is laterally wider than dorsoventrally deep, but the steep sides of the spine apices produce an asymmetric “V”-shaped bifurcation trough.

#### Cv5

**Fig 16:**
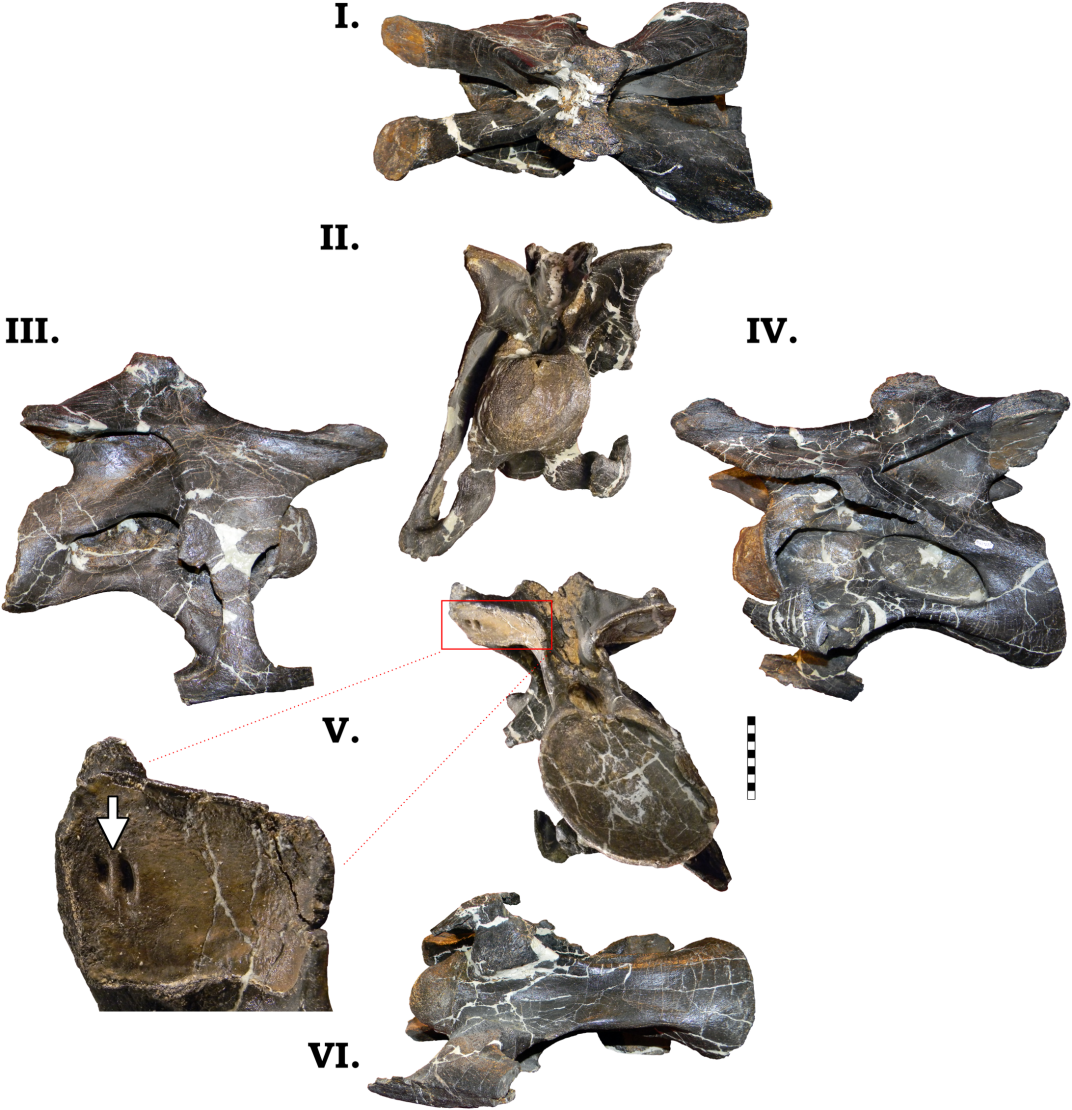
**Cervical vertebra 5 of GPDM 220 in the six anatomical planes (dorsal [I], anterior [II], right [III] and left lateral [IV], posterior [V], and ventral [VI]).** All orientations to scale. Scale bar = 10 cm. Note the red inset box in the posterior view (V) which highlights the odd, paired openings on the left postzygapophysis (indicated by the white arrow).

In the opisthocoelous fifth cervical, the large camerate pneumatic foramen are slightly angled dorsally, as in the preceding vertebra (Fig 16). In lateral profile the centrum is anteroposteriorly straight–not “kinked” (akin to GMNH-PV 101), yet the posterior region of the centrum lacks the strong ventral curvature seen in the preceding vertebra or Cv5 of GMNH-PV 101. In ventral view, the centrum is more I-Beam shaped (laterally widened condyle and cotyle with a narrow mid-centrum) as in GMNH-PV 101. The condyle is very circular (as in GMNH-PV 101), and there is a slight dorsal rim apparent in lateral view. The cotlye would appear to have been fairly circular, but taphonomic distortion has produced an angled, ovoid cotyle. As in the preceding vertebra, the cervical rib, which is fused, projects ventrally; however, in this vertebra the cervical rib is so strongly projecting that it is very reminiscent of the apatosaurine condition. As in the preceding vertebrae, the posterior portion of the diapophyses are prominently projected. Likewise this cervical further displays the bulbous articular ends of the parapophyses; yet proportionally they do not appear to be as bulbous as those documented in the preceding vertebra. The neural arches of GPDM 220 and GMNH-PV 101 are very similar. Both exhibit elongated prezygapophyses that project anteriorly from the condyle, rather dorsoventrally short neural spines, and postzygapophyses that terminate by the posterior end on the cotyle. However, in GPDM 220 minor differences are apparent: the prezygapophyses are slightly less elongate and more of a ventral curvature, and the apex of the neural spine is slightly more anteroposteriorly elongate with a slight projection as in the previous vertebra. The neural spine of GPDM 220 is more bifurcated than that of either GMNH-PV 101 or BYU 9047. In anterior view, the trough of bifurcation is laterally wider than dorsoventrally deep, producing a wide, shallow “U”-shaped trough. At the base of the bifurcation trough is a faint median tubercle.

As in Cv2 and 3, Cv5 of GPDM 220 possesses the paired openings on the left postzygapophysis. This feature is located more laterally on the postzygapophyseal facet, and the larger of the two openings is more medial. In total these openings occupy an area of slightly over 1 square cm.

#### Cv6

**Fig 17:**
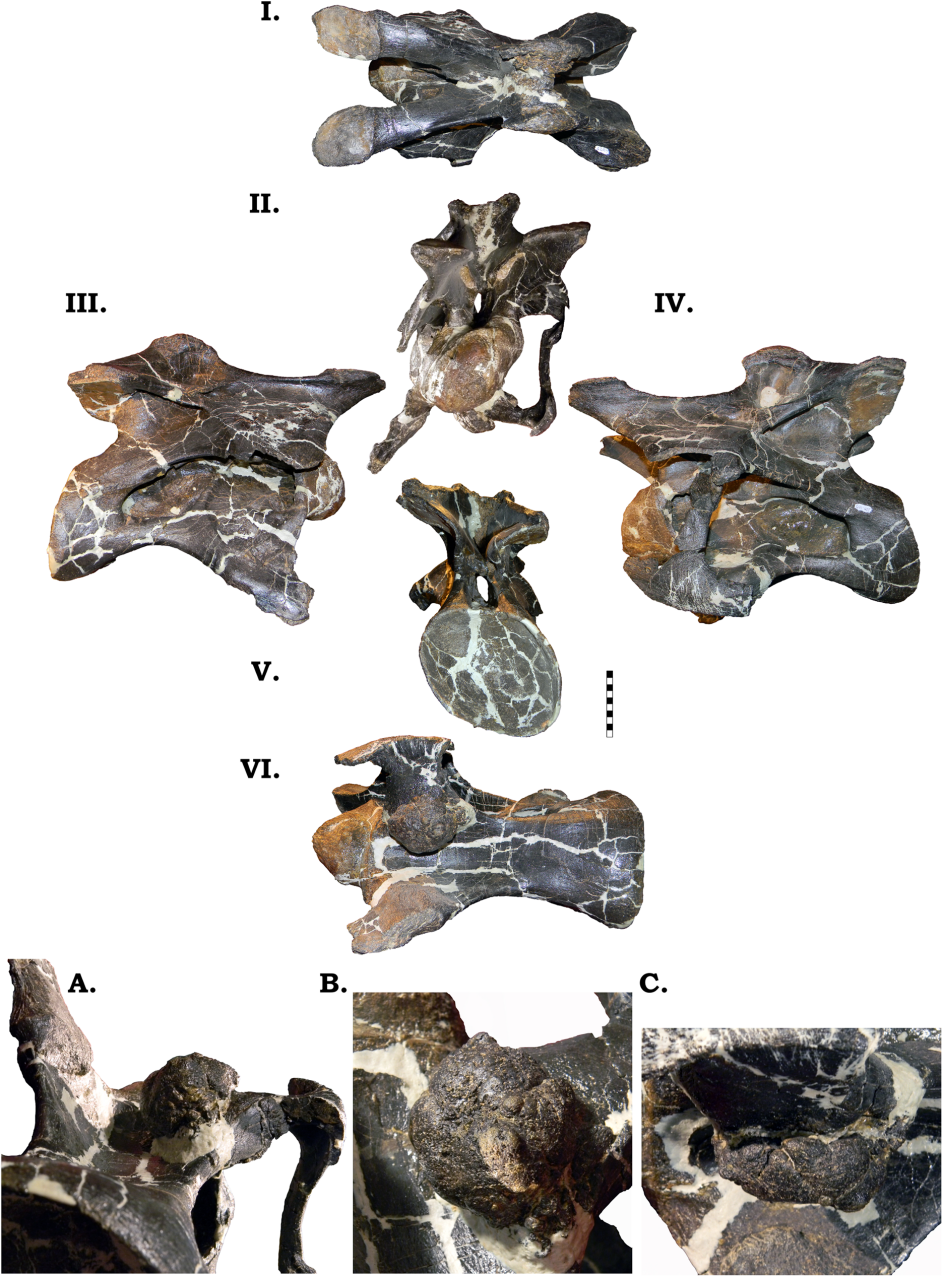
**Cervical vertebra 6 of GPDM 220 in the six anatomical planes (dorsal [I], anterior [II], right [III] and left lateral [IV], posterior [V], and ventral [VI]).** All orientations to scale. Scale bar = 10 cm. A–C illustrates the large pathology in posteroventral oblique (A), ventral (B), and lateral (C) view. A–C not to scale.

In the sixth cervical vertebra, the large camerate pneumatic fossae pervade into the centrum as in the preceding vertebrae (Fig 17). In lateral profile the centrum has a slight ventral curvature–reminiscent of the preceding “kinked” centra. The cotyle has a greater dorsoventral height than the condyle, and this gives the appearance that the cotyle is situated lower than the level of the condyle. The cotyle has a slight anterior orientation (the ventral margin of the cotyle extends more posteriorly than the dorsal rim). The condyle is subcircular with the lateral aspect being slighter greater in width than the dorsoventral height. The subcircular cotyle (wider laterally than dorsoventrally tall) is much larger than the condyle–approximately 44% greater area. In ventral orientation the centrum has the I-Beam profile as seen in GMNH-PV 101. While the diapophyses and parapophyses are damaged and distorted on both sides, they appear to be as robust as the preceding vertebrae. The parapophyses exhibit the typical bulbous posterior articular ends, and the fused and intact left cervical rib head highlights a dorsoventrally robust head. The neural arch is dorsoventrally “squat”, and reminiscent of that of GMNH-PV 101 (approximately as dorsoventrally tall as the centrum). The prezygapophyses are nearly horizontal and do not project as anteriorly as in GMNH-PV 101. The apex of the neural spine is dorsoventrally low, and the postzygapophyses terminate before the rim of the cotyle. The morphology of spine bifurcation differs between GPDM 220 and either GMNH-PV 101 and BYU 9047. While the neural spines of these specimens are dorsoventrally short, the trough of bifurcation in GMNH-PV 101 and BYU 9047 is dorsoventrally deeper than laterally wide, which produces a “V”-shaped trough. In GPDM 220, the bifurcation trough is laterally wider than dorsoventrally deep, producing a wide, shallow “U”-shaped trough. Both of the prezygapophyses and the left postzygapophysis possess the ventral foramina, but they are smaller than those observed in Cv5. Likewise the left postzygapophyseal facet weakly displays the paired foramina.

The most striking feature of Cv6 is an incredibly large pathology on the ventral side of the centrum (Fig 16A, 16B and 16C). This pathology is on the ventral portion of the left parapophysis, but it is distinctly not part of the previously observed bulbous articular facets. The pathology is roughly “mushroom”-shaped in lateral profile–there is a stout “stem” that medially projects from the parapophysis, and the bulbous “cap” is situated below the condyle. This convex “cap” has a very irregular, globular surface morphology very akin to cauliflower florets. Relative to the centrum size, this pathology is very large: anteroposteriorly 77 mm in length, lateral width 56 mm, dorsoventral height 33 mm, and a circumference of 232 mm–approximately 21% of the centrum length and 71% of the condyle width.

#### Cv7

**Fig 18:**
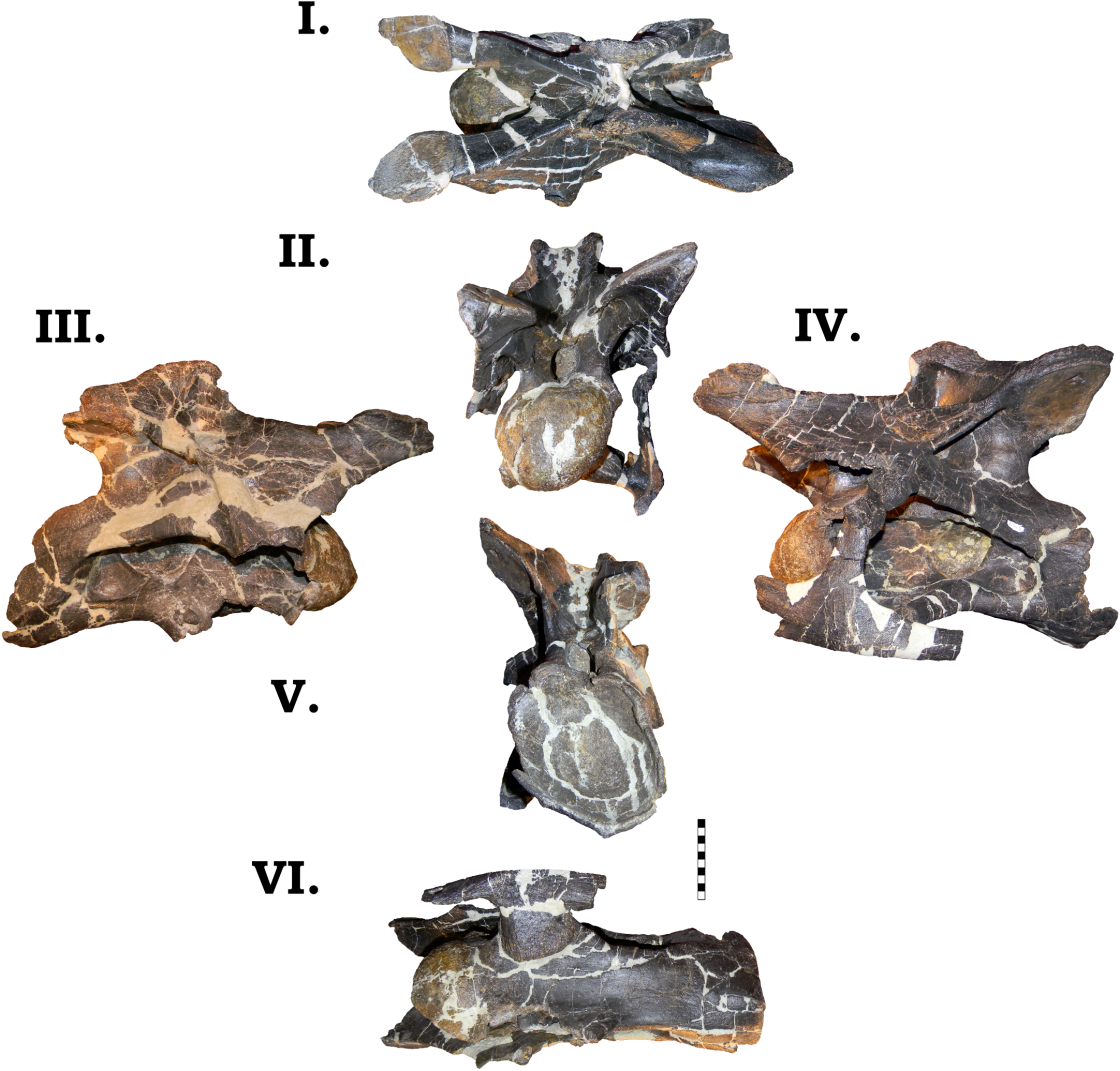
**Cervical vertebra 7 of GPDM 220 in the six anatomical planes (dorsal [I], anterior [II], right [III] and left lateral [IV], posterior [V], and ventral [VI]).** All orientations to scale. Scale bar = 10 cm.

The seventh cervical vertebra exhibits a camerate centrum that in lateral view is very anteroposterior straight (not “kinked”; Fig 18). The condyle in anterior view is circular (slightly laterally wider than dorsoventrally tall), and in lateral view, the anterior-most tip of the condyle has a slight projection. Moving ventrally from the condyle to the cotyle, there is a gentle ventral curvature that increases at the cotyle to produce a ventral margin that is well below the condyle. This morphology is very different from that of GMNH-PV 101 and BYU 9047 which exhibits a centrum that tapers posteriorly. The cotyle is slightly taphonomically distorted, so it is difficult to determine if one orientation is larger than the other. However, the circular cotyle has a greater area than the condyle; specifically it is about 41% larger. Overall this vertebra has quite of bit of damage and distortion (in comparison to the other cervicals), so some morphologies are barely visible or not discernable. In ventral orientation the centrum does not have the I-Beam nature of GMNH-PV 101. Instead the ventral aspect of the centrum is very rectangular–anterposteriorly elongate with the condyle, mid-centrum, and cotyle widths being more similar. As is GMNH-PV 101 and BYU 9047, the fused cervical rib heads are incredibly dorsoventrally robust and are reminiscent of sled runners. As in the preceding vertebrae, the parapophyses exhibit the bulbous posterior articular ends, yet in Cv7 they are less robust. Likewise, while the posterior aspect of the diapophyses are damaged on both sides, they do not appear to have been as prominent. The neural arch of GPDM 220 compared to GMNH-PV 101 and BYU 9047 are rather different. All exhibit slightly inclined, elongate prezygapophyses that anteriorly project past the condyle, yet the spine morphology between the two is very different. In GMNH-PV 101, from the prezygapophyses, the neural spine inclines posteriorly, quickly apexes in a small rounded spine tip, and then quickly merges with the postzygapophyses. In GPDM 220, from the prezygapophyses the neural spine sharply inclines near vertical with a similar anterior projection as observed in the preceding vertebrae. The neural spine apex is anteroposteriorly elongate with a slightly angled posterior portion; and after the spine apex, it quickly merges with the postzygapophyses–as in GMNH-PV 101. The left postzygapophysis exhibits the paired foramina. The degree of neural spine bifurcation also greatly differs between that of GPDM 220 and GMNH-PV 101 and BYU 9047. In GMNH-PV 101 and BYU 9047, the trough of bifurcation is dorsoventrally deeper than laterally wide, producing a deep and steep sided “V”-shaped trough. In GPDM 220 the bifurcation trough is laterally much wider than dorsoventrally deep, producing the familiar shallow and wide “U”-shaped trough.

#### Cv8

**Fig 19:**
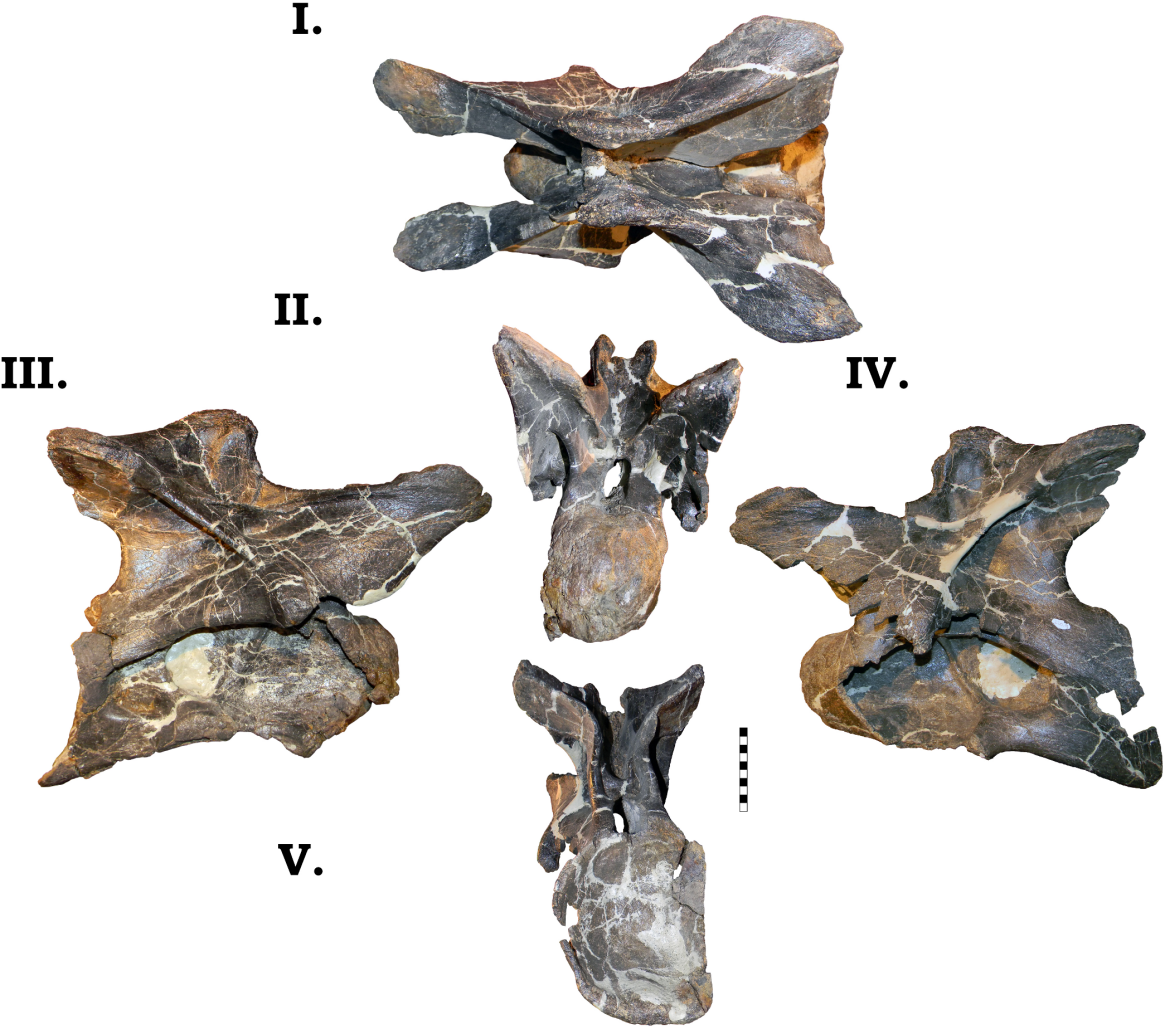
**Cervical vertebra 8 of GPDM 220 in dorsal [I], anterior [II], right [III] and left lateral [IV], and posterior [V] views.** All orientations to scale. Scale bar = 10 cm.

The centrum of the eighth cervical vertebra has widespread damage, yet it is the most anteroposteriorly elongate vertebra in the series (Fig 19; Table 1). The large camerate pneumatic fossae are more horizontally oriented relative to the centrum (not as angled as in preceding vertebrae). In anterior view the condyle is circular, being slightly dorsoventrally taller than laterally wide. In lateral view the ventral aspect of the centrum slopes posteriorly to the cotyle, producing a ventral margin of the cotyle that is well below that of the condyle. Although highly damaged, the centrum of GPDM 220 does not have the “kinked” profile nor the “cone”-like posterior region as expressed in GMNH-PV 101; in this respect it is more like that of BYU 9047. The margins of the cotyle are incomplete, but it appears to be ovoid in shape (dorsoventrally taller than laterally wide). Unfortunately the diapophyses and parapophyses are not present on either side, so we cannot infer any morphologic attributes, nor nature of cervical rib fusion. In Cv8 of GMNH-PV 101, the cervical ribs are remarkable dorsoventrally and anterioposteiorly massive–so much so that they constitute a significant portion of the centrum. The neural arches between GPDM 220 and GMNH-PV 101 have differing morphologies. While the prezygapophyses of both specimens are dorsally angled and project anteriorly past the condyle, in GPDM 220, the prezygapophyseal facet is more enlarged and has a slight ventral deflection. The neural arch and spine of GMNH-PV 101 is dorsoventrally low, and the neural spine is strongly posteriorly angled, terminating is a short “isosceles” spine apex. In GPDM 220, the entire arch complex is dorsoventrally taller than the centrum (approximately 1.5 times). Posteriorly from the prezygapophyses, the neural spine sharply rises (with the previously observed anterior projection), slightly plateaus, and then quickly merges into the postzygapophyses. As previously observed, the right prezygapophysis and the left postzygapophysis possesses the paired openings. While the arches between GPDM 220 and GMNH-PV 101 greatly differ, the degree of spine bifurcation is more akin. In GMNH-PV 101, the trough of bifurcation is dorsoventrally deep (approximately half the height of the cotyle), while the width between the spines is narrow–producing a deep, steep sided “U”-shaped trough. In GPDM 220 the trough of bifurcation is not as deep as GMNH-PV 101, but the overall morphology is similar in that the trough is dorsoventrally deeper than laterally wide, producing a steep sided trough.

#### Cv9

**Fig 20:**
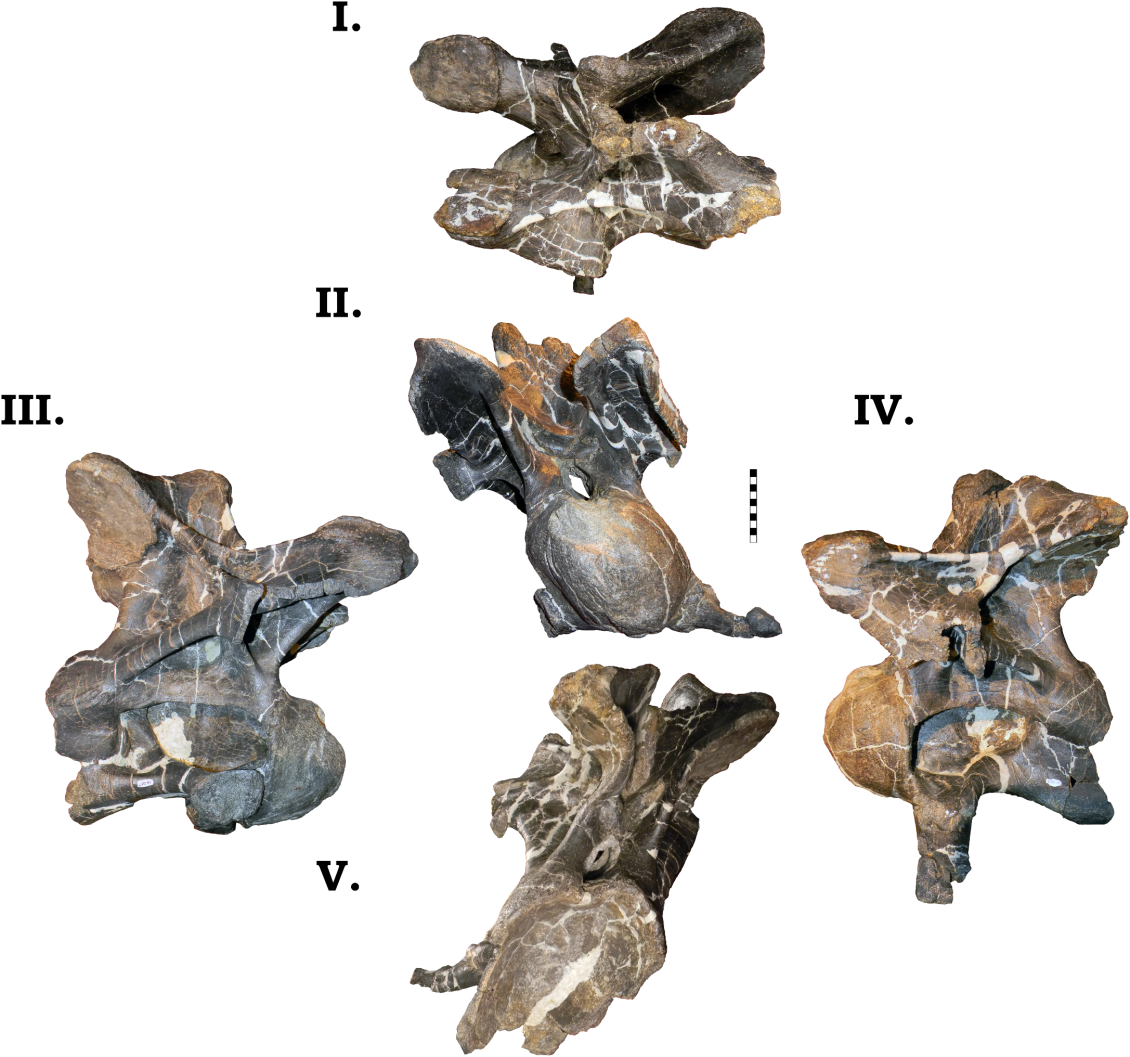
**Cervical vertebrae 9 of GPDM 220 in dorsal [I], anterior [II], right [III] and left lateral [IV], and posterior [V] views.** All orientations to scale. Scale bar = 10 cm.

Unfortunately GMNH-PV 101 does not have any cervicals posterior to cervical 8, so no further comparisons between the cervical series of GPDM 220 and GMNH-PV 101 can be made (Fig 20). The centrum of cervical nine is strongly opisthocoelous, and the pneumatic fossae are anteroposteriorly elongate and camerate. In lateral view the centrum has a steep ventral deflection, yet the centrum does not exhibit the overall “kinked” profile as some of the preceding vertebrae; nor the strongly “cupped” posterior profile of BYU 9047. The anterior face of the condyle is damaged, but it is strongly circular (slightly laterally wider than dorsoventrally tall). In lateral view, the ventral margin of the centrum steeply inclines dorsally towards the midpoint of the centrum, and has a near matching angle of incline from the cotyle. The ventral ~1/3 of the cotyle is missing, but it appears that in overall morphology it is ovoid (dorsoventrally taller than laterally wide). The prezygapophyses have enlarged, ovoid articular facets, and overall the prezygapophyses are very elongate (approximately 2/3 the length of the centrum), and they are inclined approximately 31° above the level of the centrum. The diapophyses are missing on either side, but the parapophyses are intact, and coupled with the size of the parapophyseal facets and the medial margin of the costotransverse ansa, suggests an enlarged and robust cervical rib (potentially similar to that seen in cervical 8 of GMNH-PV 101). Interestingly, in right lateral view it would appear that the cervical rib was unfused–unlike the preceding 4 cervicals. Yet it would appear that part of the rib is present and attached on the left side. Unfortunately the damage to the articular facets prevents definitive identification. In lateral profile the neural arch is dorsoventrally elongate–approximately 1.5 times the centrum height. Posteriorly from the prezygapophyses, there is a steep ventral deflection, then an abrupt and steeper incline to the neural spine. The neural spine is dorsoventrally short, and after the spine apex it quickly and gentle grades into the postzygapophyses. The ovoid postzygapophyseal facets of this cervical are very large–nearly as anteroposteriorly long as the pneumatic foramen in the centrum. The margins of the postzygapophyseal facets are damaged, but conservatively, restored borders suggest a surface area of approximately 54 square cm. As in cervical 5, the postzygapophyseal facets exhibit the unusual paired openings. No pathologies or additional vertebral oddities are evident or apparent on this vertebra. The neural spine is dorsoventrally short, and in anterior view the dorsoventral depth and lateral width of the bifurcation trough is nearly equal. The steep sided spine apices coupled with the trough dimensions produce a steep sided “U”-shaped trough. At the ventral portion of the trough is a prominent and laterally wide median tubercle.

#### Cv10

**Fig 21:**
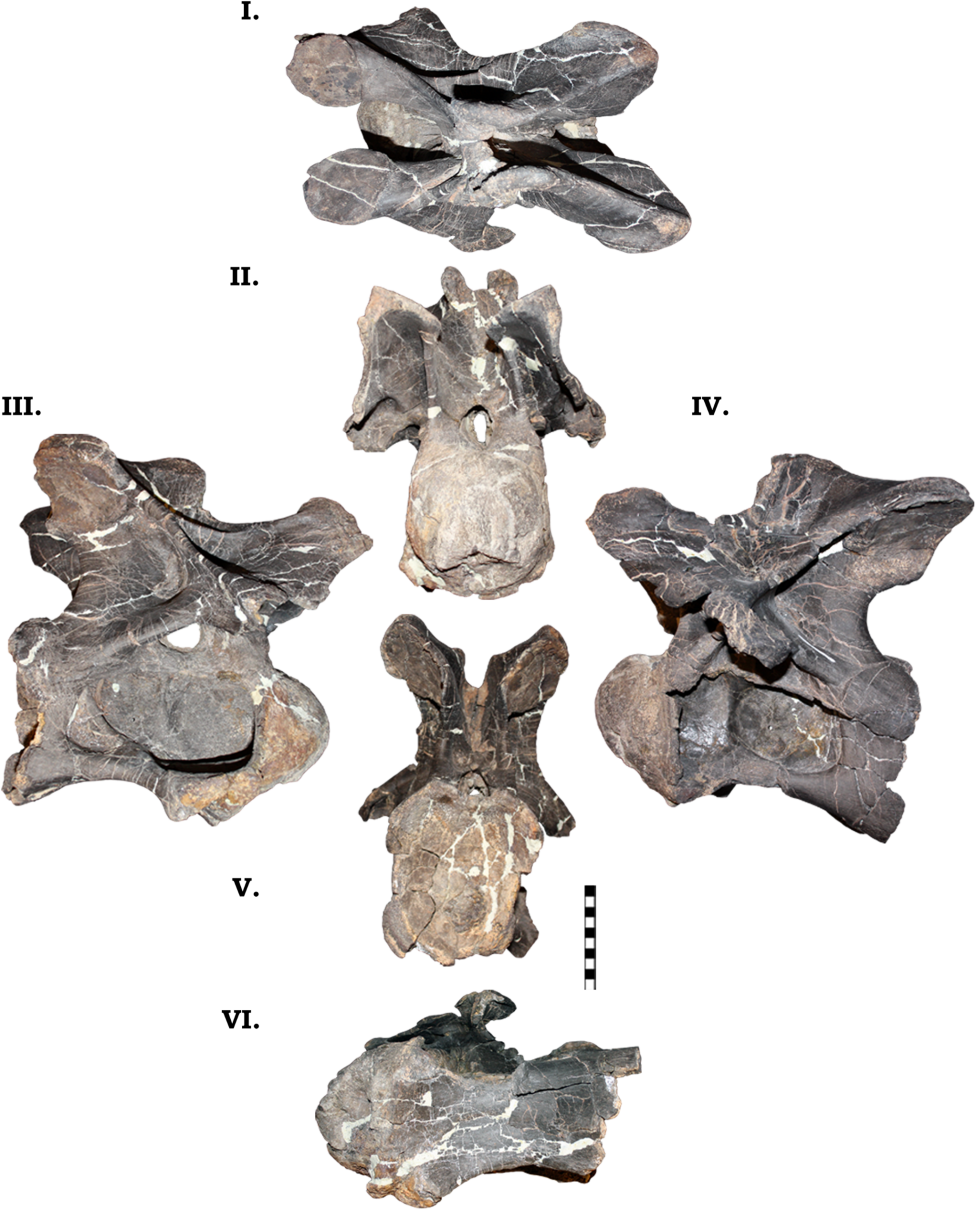
**Cervical vertebra 10 of GPDM 220 in the six anatomical planes (dorsal [I], anterior [II], right [III] and left lateral [IV], posterior [V], and ventral [VI]).** All orientations to scale. Scale bar = 10 cm.

The centrum of GPDM 220 cervical 10 is strongly opisthocoelous, and the pneumatic fossae are proportionally larger than observed in any of the preceding vertebrae (Fig 21). These camerate fossae seem to possess a reduced, almost “vestigial” accessory lamina, resulting in what appears to be a single, large, sub-rectangular fossa. In lateral view the centrum is very anteroposterior straight, and does not exhibit any of the deflection or “kinked” morphology seen previously. Progressing posteriorly to the cervicodorsal transition, the anteroposterior length of the centrum decreases while the dorsoventral height of the neural arch increases, and Cv10 continues to show this trend. In anterior view, the ventral portion of the condyle is damaged, yet the overall circular morphology can still be readily discerned (just slightly dorsoventrally taller than laterally wide). The ventral aspect of the centrum is very damaged–missing the entire ventral margin of the cotyle, but in overall morphology it appears to have wide anterior and posterior ends while tapering mid-centrum length to produce an overall “hourglass” profile. While the margins of the cotyle are highly damaged, in posterior view the cotyle appears to have an ovoid profile (dorsoventrally taller than laterally wide). The prezygapophyses have quite a dorsoventral profile with large anteroposterior elongate facets, but they only extend anteriorly from the condyle a few centimeters. Neither lateral side preserves the diapophyses and parapophyses, yet given the preceding examples and the remnants of the parapophyseal facet, the cervical ribs were likely anteriorly robust and potentially unfused or partially fused. In lateral profile the neural arch is dorsoventrally taller than the centrum (approximately 1.5 times). Posteriorly from the prezygapophyses, there is a steep ventral deflection, then an abrupt and steeper incline–near vertical—to the neural spine (as seen in C9). The neural spine is dorsoventrally short, and after the spine apex it quickly and gentle grades into the enlarged postzygapophyses. As in Cv9, the postzygapophyseal facets are enlarged and very ventromedially oriented. Likewise, the lateral most margins of the left postzygapophysis possess the paired openings. In anterior view, the neural spine is dorsoventrally short, and the dorsoventral depth and lateral width of the bifurcation trough is nearly equal. The steep sided spine apices coupled with the trough dimensions produce a steep sided “U”-shaped trough. At the ventral portion of the trough is a laterally wide median tubercle.

#### Cv11

**Fig 22:**
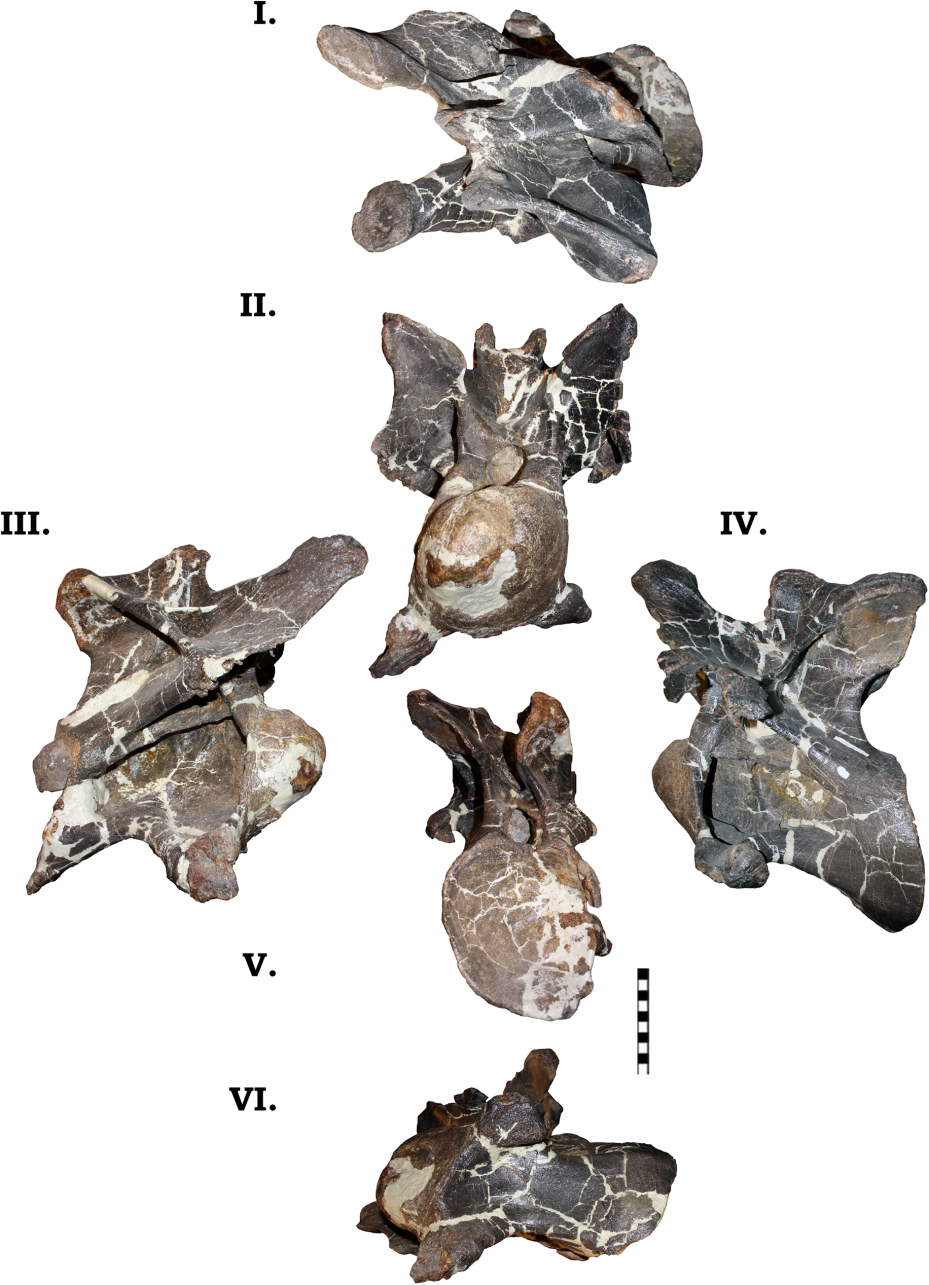
**Cervical vertebra 11 of GPDM 220 in the six anatomical planes (dorsal [I], anterior [II], right [III] and left lateral [IV], posterior [V], and ventral [VI]).** All orientations to scale. Scale bar = 10 cm.

The last cervical vertebrae present in GPDM 220 is Cv11 (Fig 22). In lateral view the entire profile of this cervical is highly anteriorly rhomboidal. As in the preceding cervical, the large camerate pneumatic fossa occupies almost the entire lateral area of the centrum. In lateral view the great rhomboidal displacement gives the illusion that the midpoint of the condyle and cotyle are on differing planes (the condyle being more dorsally situated of the two). In anterior view the condyle is strongly circular (nearly equal in dorsoventral height and lateral width). The prezygapophyses prominently extend laterally from the condyle (approximately half the condyle width); likewise they extend dorsally above the apices of the neural spine. In ventral view Cv11 exhibits the commonly occurring “hourglass” profile–anterior and posterior ends wider and tapering mid-centrum. In posterior view, while a portion of the right lateral margin of the cotyle is damaged, the cotyle is very circular (slightly dorsoventrally taller than laterally wide). The prezygapophyses of Cv11 are the most elongate yet observed in GPDM 220. In lateral view the prezygapophyses are approximately half the anteroposterior length of the centrum, and extend approximately 6 cm anteriorly past the condyle. As in Cv10, the prezygapophyses have quite a dorsoventral profile with large anteroposterior elongate facets. As seen in preceding cervicals, neither lateral side preserves intact diapophyses nor parapophyses with cervical ribs, and thus the nature of fusion is indeterminable. The largely intact parapophyses exhibit the bulbous posterior articular ends, and these remnants combined with the medial margin of the costotransverse ansa allude to massive and robust cervical ribs. In lateral view the neural arch is approximately 2 times the dorsoventral height of the centrum. Posteriorly from the prezygapophyses, there is a long and gentle incline before reaching the base of the neural spine. At the junction the neural spine strongly vertically projects, with a slight anteriorly oriented projection (similarly observed in many of the preceding cervicals). The neural spine itself is very dorsoventrally short, and after the spine apices it quickly grades into the enlarged postzygapophyses. As in Cv10, the postzygapophyseal facets are enlarged and very ventromedially oriented. Both the left prezygapophysis and postzygapophysis weakly display the paired opening as seen previously. As previously seen, the neural spine is dorsoventrally short. The lateral width of the neural spines is slightly greater than the dorsoventral depth of the bifurcation trough, producing a wide “U”-shaped trough. The ventral portion of the trough displays a slight median tubercle.

#### Dorsal vertebrae

From GPDM 220 there are only two complete dorsal vertebrae. In comparison to GMNH-PV 101—which is only represented by dorsals (D) 6–11 –the two dorsals of GPDM 220 have bifurcated neural spines, unlike those of GMNH-PV 101, therefore we believe these two to be anterior to D6 (however, note that the articulated series of *C*. *lewisi*– D7-11 –possesses bifurcated neural spines [21]). Based on comparison to other *Camarasaurus* dorsal material, we believe the two dorsal vertebrae from GPDM 220 are D1 and D2.

#### D1

**Fig 23:**
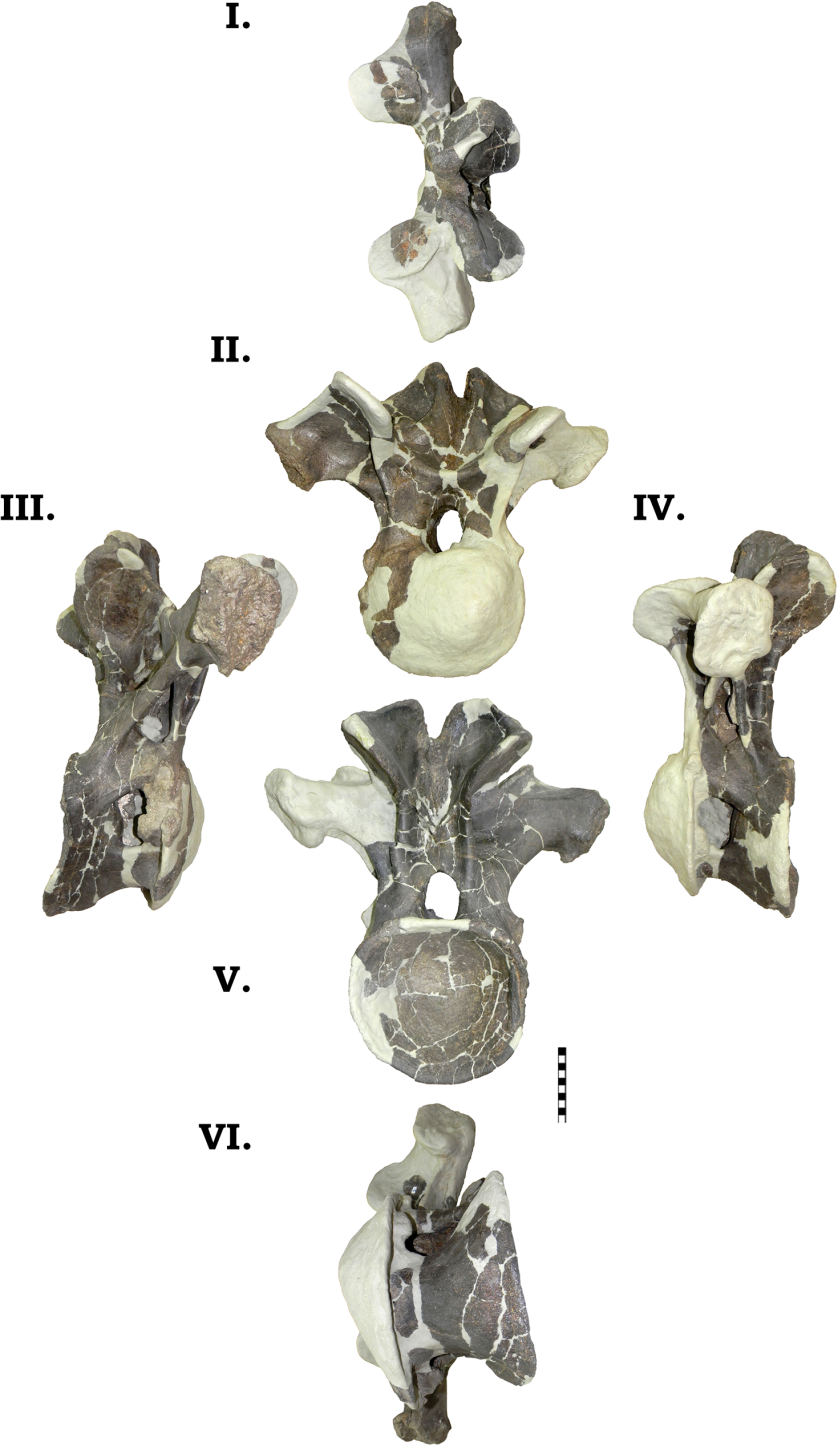
**Dorsal vertebra 1 of GPDM 220 in the six anatomical planes (dorsal [I], anterior [II], right [III] and left lateral [IV], posterior [V], and ventral [VI]).** All orientations to scale. Scale bar = 10 cm.

D1 of GPDM 220 is not complete, and a significant portion has been modeled, but the overall condition of the vertebra is great (Fig 23). In lateral view the overall profile is that of a dorsoventrally elongate rhomboid. In the centrum there is a large pneumatic fossa that occupies almost the entire lateral aspect of the centrum. Anterior and ventral to the pneumatic fossa is a prominently projecting parapophysis. As in the last cervical vertebra, the great displacement of the ventral margins of the condyle and cotyle gives them the appearance of being of different planes. In anterior view, the condyle very circular (both orientations being sub equal). In posterior view, the entire margin of the condyle is reconstructed, but if it is an accurate reflection, the condyle is likewise very circular–slightly dorsoventrally taller than laterally wide. In posterior view, the centrum is anteroposteriorly short, and laterally wide–reminiscent of a square in overall profile. As observed in some of the preceding cervical vertebrae, the mid-point of the centrum is “pinched” medially, producing an “hourglass” shape–albeit not to the same degree as observed in the cervicals. In ventral and lateral view is a demarcated ring around the condyle. This raised margin is posterior to the condyle, and is prominent and continuous along the condyle. In lateral view the neural arch is dorsoventrally elongate–over 2 times the height of the centrum. The prezygapophyses anteriorly extend approximately 10 cm past the condyle. The prezygapophyseal facets are sub-circular and very large–approximately 10 cm by 10 cm in anteroposterior length and lateral width. Proceeding posteriorly from the prezygapophyses, there is a sharp ventral incline to the base of the neural spine. The neural spine steeply rises, and after the apex quickly grades into the postzygapophyses. The postzygapophyses in fact are dorsally higher than the apex of the neural spine. The postzygapophyseal facets, like the prezygapophyseal facets, are incredibly enlarged. The postzygapophyseal facets are dorsoventrally oriented, and they are ovoid in profile–approximately 13 cm dorsoventrally by 10 cm anteroposteriorly. Neither transverse process is complete, but they appear to project laterally from the centrum approximately 20 cm. As seen previously in many of the cervical vertebrae, the neural spine is dorsoventrally short. The lateral width of the neural spines is sub-equal to the dorsoventral depth of the bifurcation trough, producing a steep sided “U”-shaped trough. Unlike the cervical vertebrae, there does not appear to be a protruding median tubercle in the ventral portion of the trough.

#### D2

**Fig 24:**
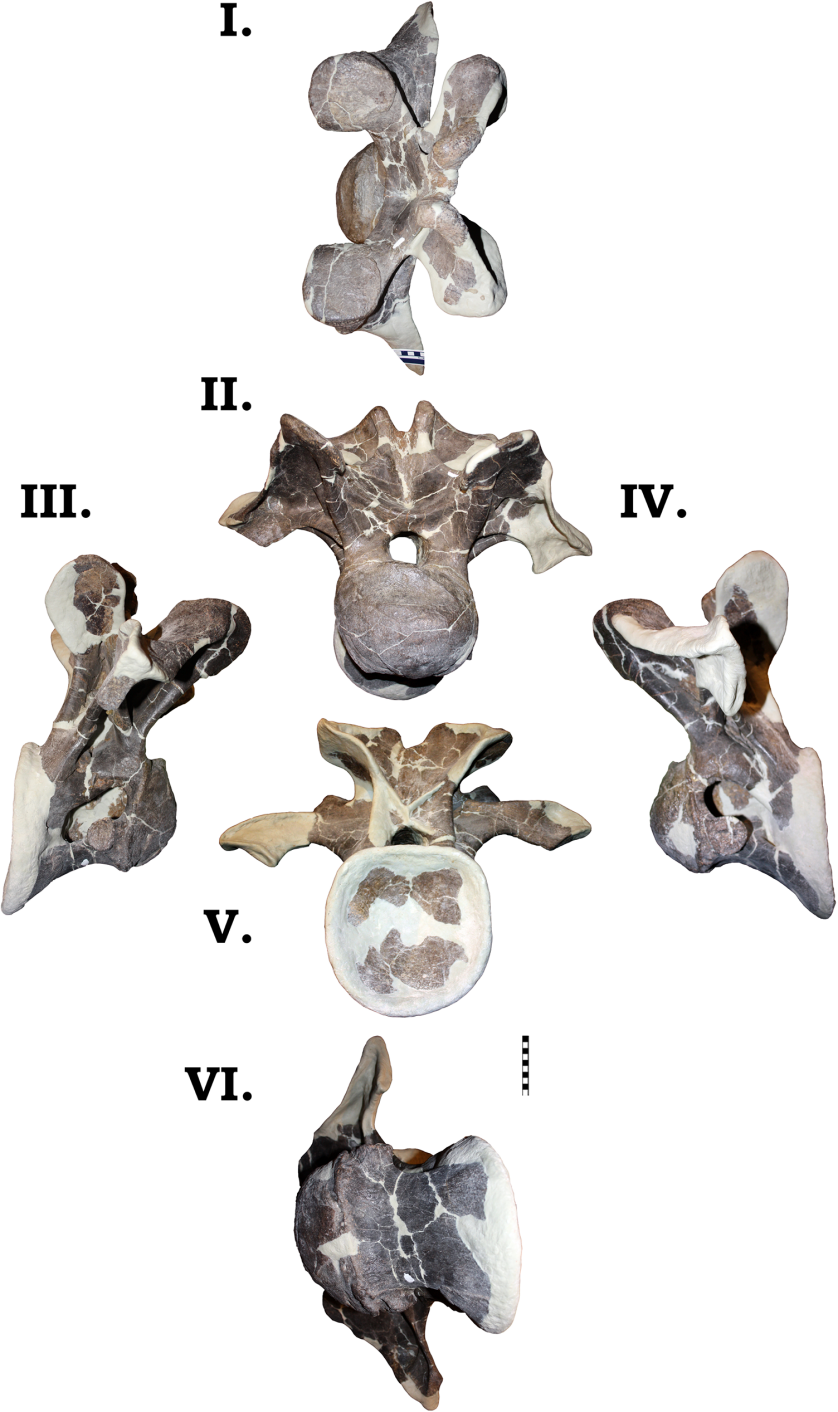
**Dorsal vertebra 2 of GPDM 220 in the six anatomical planes (dorsal [I], anterior [II], right [III] and left lateral [IV], posterior [V], and ventral [VI]).** All orientations to scale. Scale bar = 10 cm.

The last dorsal vertebrae present in GPDM 220 is that of D2 (Fig 24). As in D1, much of the vertebrae has been restored, but the overall condition of the bone is pristine. D2 exhibits a rhomboid lateral profile similar to D1. The centrum is strongly opisthocoelous (further supporting an anterior serial position), and there is a large pneumatic fossa in the anterior half of the centrum. The pneumatic fossa is smaller in D2 than in D1. As in D1, there is a prominent parapophysis, but it is smaller than that of D1, and more ventral to the pneumatic fossa. In anterior view, almost all of the condyle is missing and reconstructed; but if it has been reconstructed accurately, the condyle is circular, being slightly laterally wider than dorsoventrally tall. In posterior view, the cotyle is nearly complete; and like the condyle, it is circular, being slightly laterally wider than dorsoventrally tall. The profile in ventral view of D2 is very similar to that of D1. The centrum is anteroposteriorly short and laterally wide. There is a bit of midline “kink” (i.e. “hourglass” profile) as in D1, but in D2 this “kink” is not as strong, and the centrum is more square-shaped. In lateral view, the neural arch is very tall–over two times the height of the centrum. The prezygapophyses anteriorly project from the condyle under 10 cm, and in dorsal view the prezygapophyseal facets are large and sub-circular as in D1 (approximately 10 cm by 10 cm in anteroposterior length and lateral width). Posteriorly from the prezygapophyses, there is a steep, ventral decline to the base of the neural spine. The neural spine is steeply vertically inclined with an anteroposteriorly more elongate spine apex than in D1. After the spine apex it quickly grades into the postzygapophyses. As in D1, the postzygapophyses of D2 have large, ovoid facets (again approximately 13 cm dorsoventrally by 10 cm anteroposteriorly). Only the right transverse process is complete, but they appear to project laterally approximately 10 cm from the condyle, and the lateral ends have large, robust tuberculum facets. As in D1, the neural spine is dorsoventrally short. The lateral width of the neural spines is sub-equal to the dorsoventral depth of the bifurcation trough, but it tapers medially, producing a steep sided “U”-shaped trough. Likewise, there does not appear to be a protruding median tubercle in the ventral portion of the bifurcation trough.

#### Dorsal ribs

A large portion of the fragments recovered from GPDM 220 constitute dorsal rib fragments. These fragments will not be described or attempted to be serial positioned. The only dorsal ribs to be described here are ones with intact heads which allow for serial positioning.

**Fig 25:**
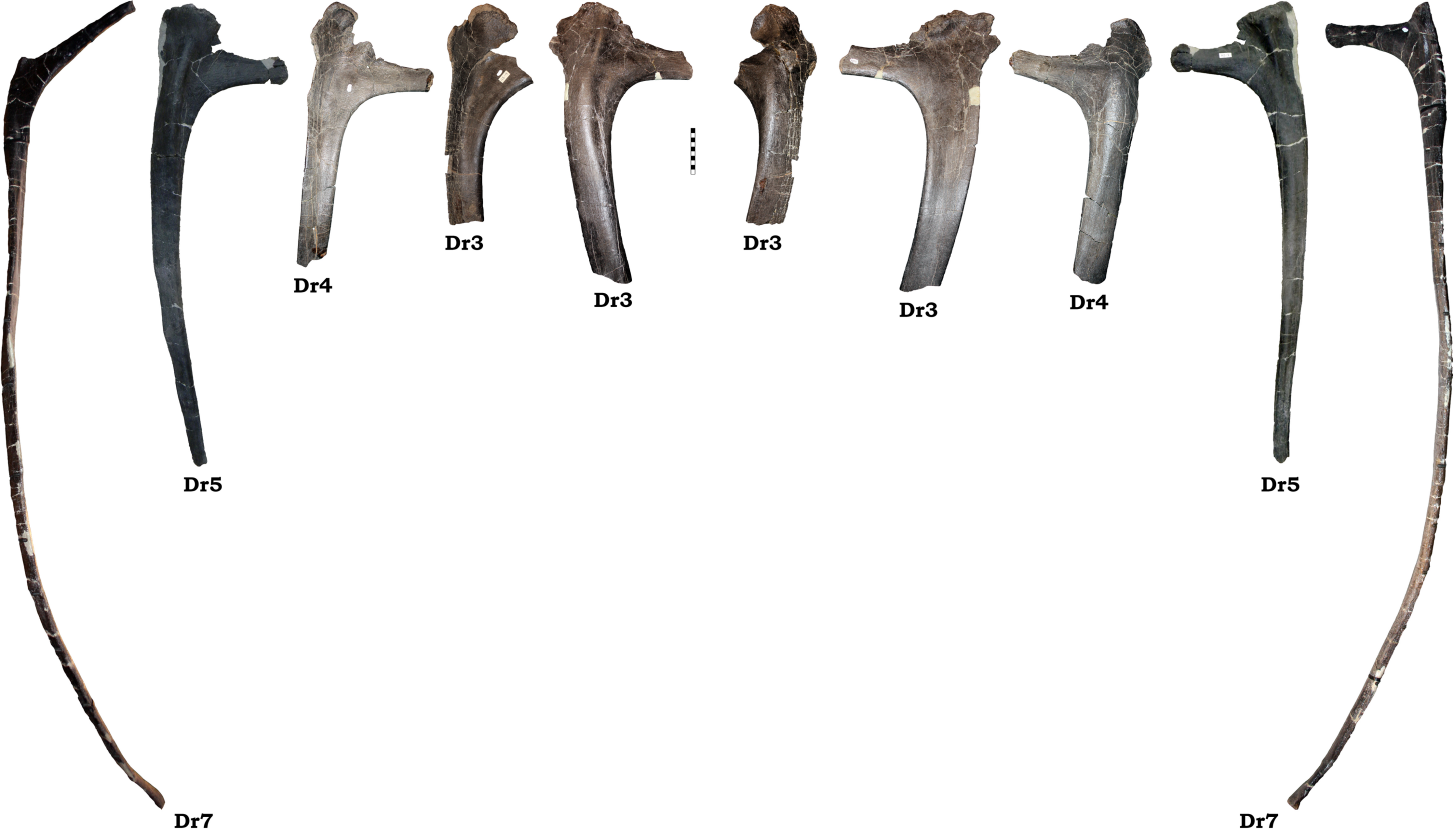
Serially identifiable dorsal ribs of GPDM 220. All orientations to scale. Scale bar = 10 cm.

Out of a total of 24 dorsal ribs, only five have attached heads in GPDM 220 (Fig 25). Comparisons to known *Camarasaurus* rib series were used to help identify serial position. Unfortunately the best ribs series documented in the *Camarasaurus* literature belong to different species, GMNH-PV 101 (*C*. *grandis*), BYU 9047 (*C*. *lewisi*), and SMA 0002 (*C*. *lewisi*). While there are some morphologic trends between these species (such as size and orientation of the capitulum and tuberculum), the differences makes direct comparisons difficult. Likewise, comparing the dorsal ribs of GPDM 220, there is a great degree of morphological difference. Collectively, the dorsal ribs of GPDM 220 are more like those of GMNH-PV 101 and SMA 0002 than BYU 9047. The dorsal ribs of GPDM 220 (and GMNH-PV 101 and SMA 0002) generally have more enlarge or robust heads, and the shafts have less degree of curvature than BYU 9047. Serial positions for the GPDM 220 dorsal ribs have been hypothesized, but note that the differing morphology could result in slighting differing positioning–however, we do believe our serial assignments to be extremely close approximations.

#### Dr3

The first identifiable dorsal rib from GPDM 220 is rib 3. For this serial position, we believe that we have both left and right counterparts. Both sections constitute the approximately 40 cm proximal most portion of each rib. The tuberculum of both ribs is laterally wide and broadly triangular in shape. The tuberculum of the left Dr3 is damaged proximally, but its overall morphology corresponds to that of its counterpart. The capitulum for neither thoracic rib is complete, and the one for the left rib is the more intact. The capitulum is under 20 cm in length, and it quickly tapers from the tuberculum and the neck of the shaft. In lateral view, the shaft of both Dr3’s is laterally wide, yet they appear to taper distally rather quickly. In relation to the tuberculum and capitulum, the shaft of these thoracic ribs appears to strongly medially curve, producing an overall rib morphology that is under 90°.

#### Dr4

The second identifiable dorsal rib from GDPM 220 is rib 4. As in the preceding Dr3, this rib is represented by the fragmentary ~45 cm proximal most portion. Constituting the left Dr4, the tuberculum is laterally wide and triangular (as in the Dr3s); although it is slightly more dorsoventrally tall than laterally wide than the preceding tubercula. The capitulum is elongate as in the preceding thoracic ribs, yet it tapers more quickly than in the Dr3s. The shaft of the rib is laterally narrower than that of the preceding Dr3s, and while proximally the shaft tapers, it is much more gradual. In relation to the tuberculum and capitulum, as in the preceding Dr3s, the orientation of the rib head to shaft produces an overall angle under 90°. Yet this angle is greater than that of the Dr3s and closer to 90°. Dr4 was histologically sampled.

#### Dr5

The third identifiable dorsal rib from GDPM 220 is rib 5. Representing the right Dr5, this dorsal rib is nearly complete–missing only the distal most portion. The tuberculum is laterally wide and dorsoventrally tall, yet unlike the widened triangular profile in the preceding ribs, the tuberculum in Dr5 is very square shape with a somewhat flattened apex. The capitulum tapers medially, and terminates in a flared articular facet. The shaft of the rib is laterally narrower than in the preceding dorsal ribs, and it gentle tapers to a few cm in width at the distal most end. Opposed to the preceding dorsal ribs, the orientation of the rib head and shaft produces an overall angle over 90°.

#### Dr7

The last identifiable rib from GPDM 220 is rib 7. This is the most complete of all of the GPDM 220 dorsal ribs–this rib is fully intact. The rib head is very gracile in comparison to the previous ribs; the tuberculum is dorsoventrally elongate, and very laterally narrow, producing an “isosceles triangle” profile. The capitulum is narrow, but it does not strongly taper as in the preceding thoracic rib. The capitulum terminates in a flared and prominent articular facet. The orientation of the tuberculum and capitulum are almost perpendicular, producing a rib head with a strong 90° profile. The shaft of the rib is ~1.7 m long, and distal to the tuberculum and capitulum, the shaft quick tapers, yet maintains a fairly uniform thickness throughout. Approximately 2/3 of the shaft exhibits little curvature–effectively straight, but the distal most 1/3 of the shaft suddenly and strongly curves medially before terminating in the costal articulation. As observed in the Dr5, the orientation of the rib head and shaft produces an overall angle greater than 90°. While this overall morphology may be legitimate, given that dorsal ribs are elongate, thin elements, shape and curvature can easily be taphonomically distorted.

#### Caudal vertebra

**Fig 26:**
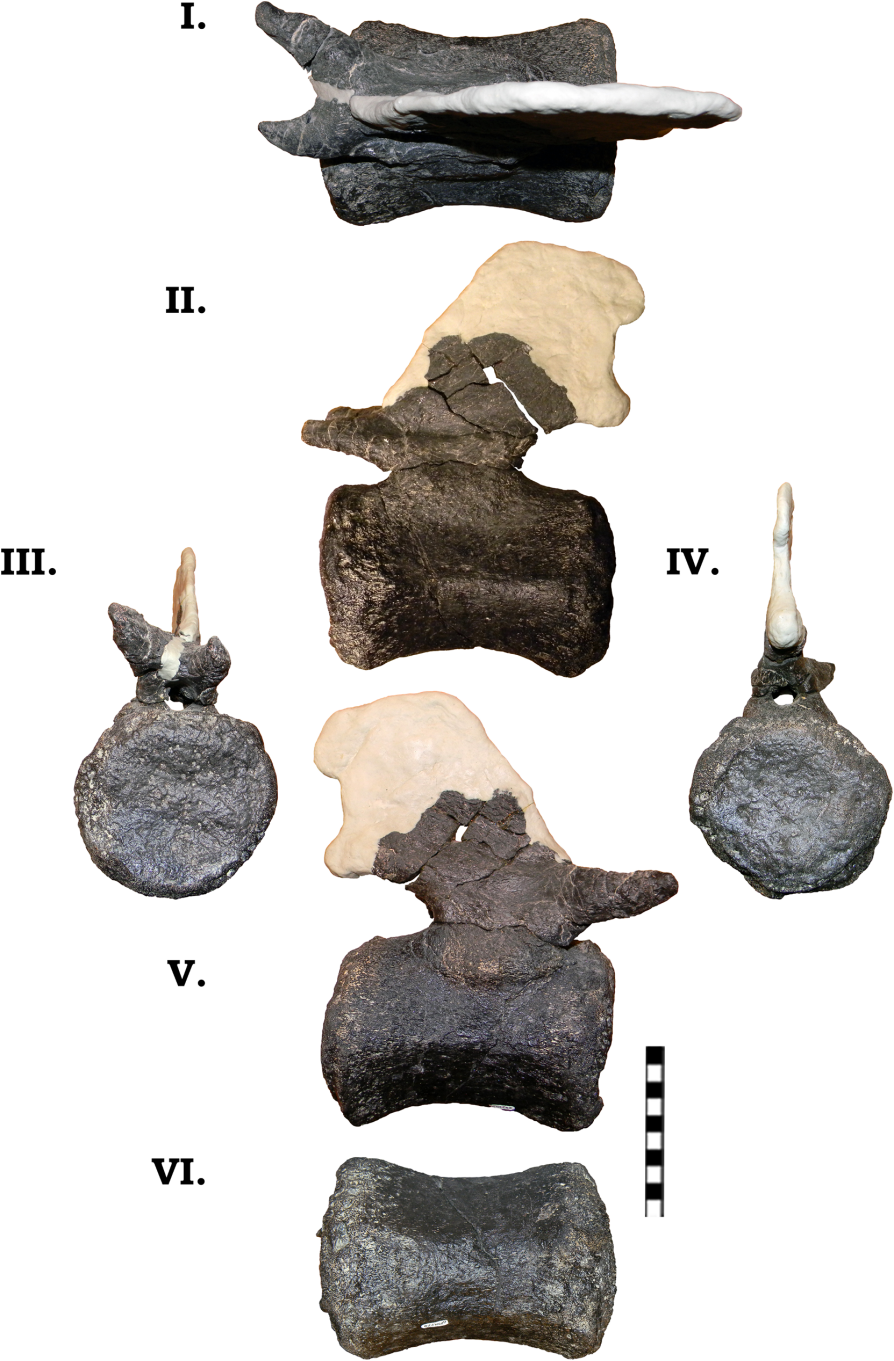
**Caudal vertebra of GPDM 220 in the six anatomical planes (dorsal [I], anterior [II], right [III] and left lateral [IV], posterior [V], and ventral [VI]).** All orientations to scale. Scale bar = 10 cm.

There is a single caudal vertebra represented from GPDM 220 (Fig 26). The centrum is anteroposteriorly more elongate than dorsoventrally tall, and is incipiently procoelous to amphiplatyan. Both anterior and posterior faces of the centrum are strongly circular and widened—laterally and dorsoventrally. In ventral view, the centrum gently tapers medially, giving the centrum a “spool”-shaped profile. The prezygapophyses are nearly complete, and they anteriorly project sub horizontally past the anterior face. There are no transverse processes present, but slight laterally projecting keels attest that serially, this vertebra is posterior to those with full processes. The neural spine is not complete and is missing virtually the entire spinous process. The base of the spine is anteroposteriorly robust, which would suggest that the spine was largely rectangular in lateral profile (as is reconstructed). While we cannot determine an exact caudal position, serially, based on the anteroposteriorly elongate centrum, rectangular neural spine, and lack of transverse processes, we suggest that this vertebra is situated between caudal 20–27.

#### Chevron

**Fig 27:**
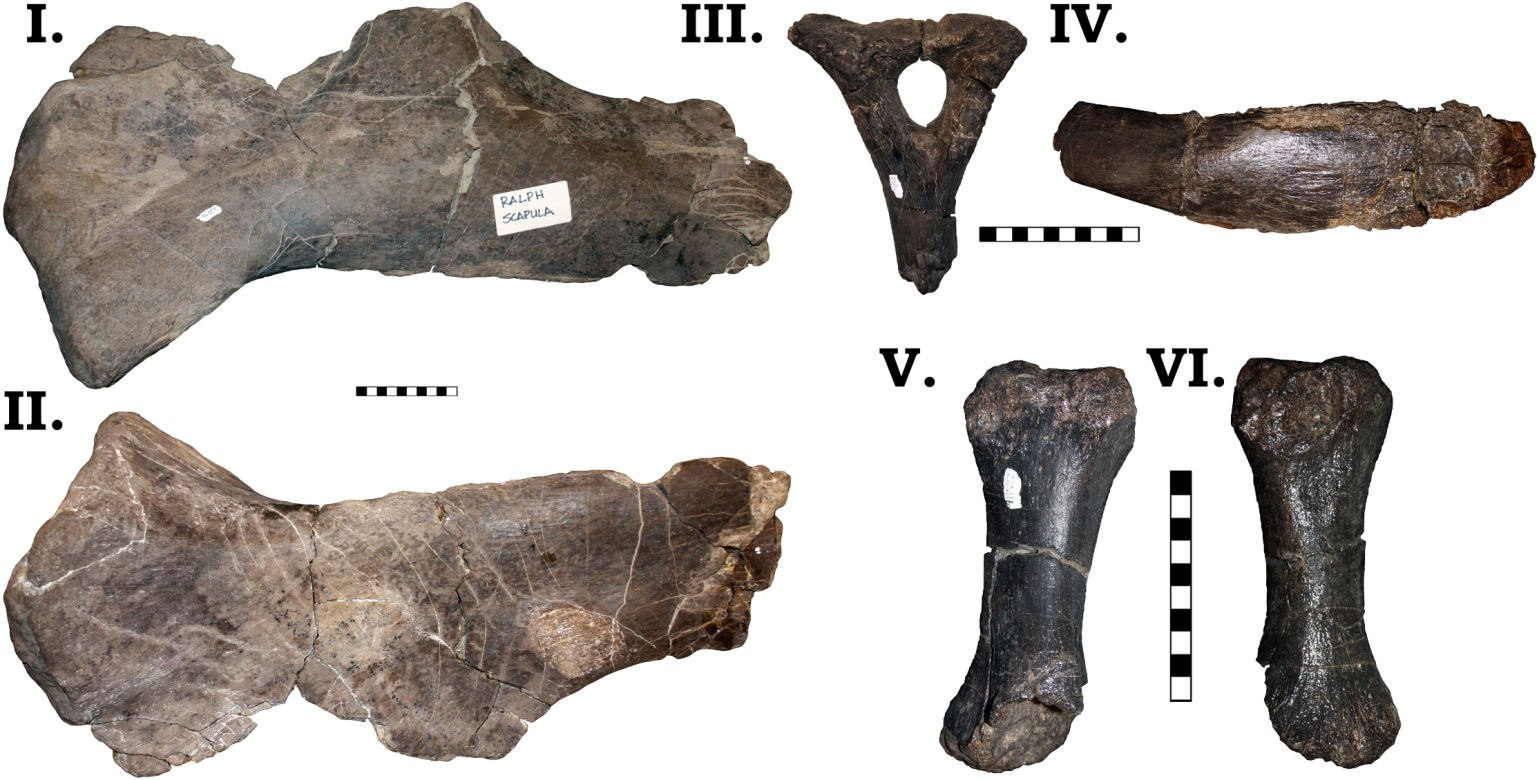
**Incomplete left scapula–in left lateral (I) and medial (II) views, single caudal chevron–in anterior (III) and left (IV) lateral views, and single right metatarsal–in anterior (V) and posterior (VI) views, of GPDM 220.** Elements not to scale with one another. Scale bar = 10 cm.

There was one chevron recovered for GPDM 220 (Fig 27). This chevron is fragmentary, but it represents both proximal and distal portions. In anterior view, the rami are the chevron are dorsoventrally short and forked (i.e., “Y”-shaped, [22]), but the articular ends of the rami are connected, producing a pseudo “fenestra”. In lateral view the blade is posteriorly kinked with an anteroposteriorly widened distal section. From the rami morphology, articular connections, and “blade”-like curvature, in comparison to articulated *Camarasaurus* tail sequences (particularly BYU 9047), we theorize that this chevron is serially from chevron 13–22.

#### Appendicular. Scapula

The only portion of the pectoral girdle represented in GPDM 220 is a fragmentary scapula (Fig 27). The fragmentary left scapula is only represented by a partial anterior portion. A section of the scapular blade is present—represented by the scapular neck, the entire acromion process is missing, and the most complete and intact portion is the proximal plate. The proximal plate is laterally wide, and strongly deflects anteroventrally as in GMNH-VP101. The scapular contribution to the glenoid fossa is nearly intact; unfortunately neither the coracoids nor humeri are represented.

#### Hind limb

The hind limbs of GPDM 220 are represented by 4 elements: an incomplete femur, tibia, fibula, and metatarsal.

#### Femur

**Fig 28:**
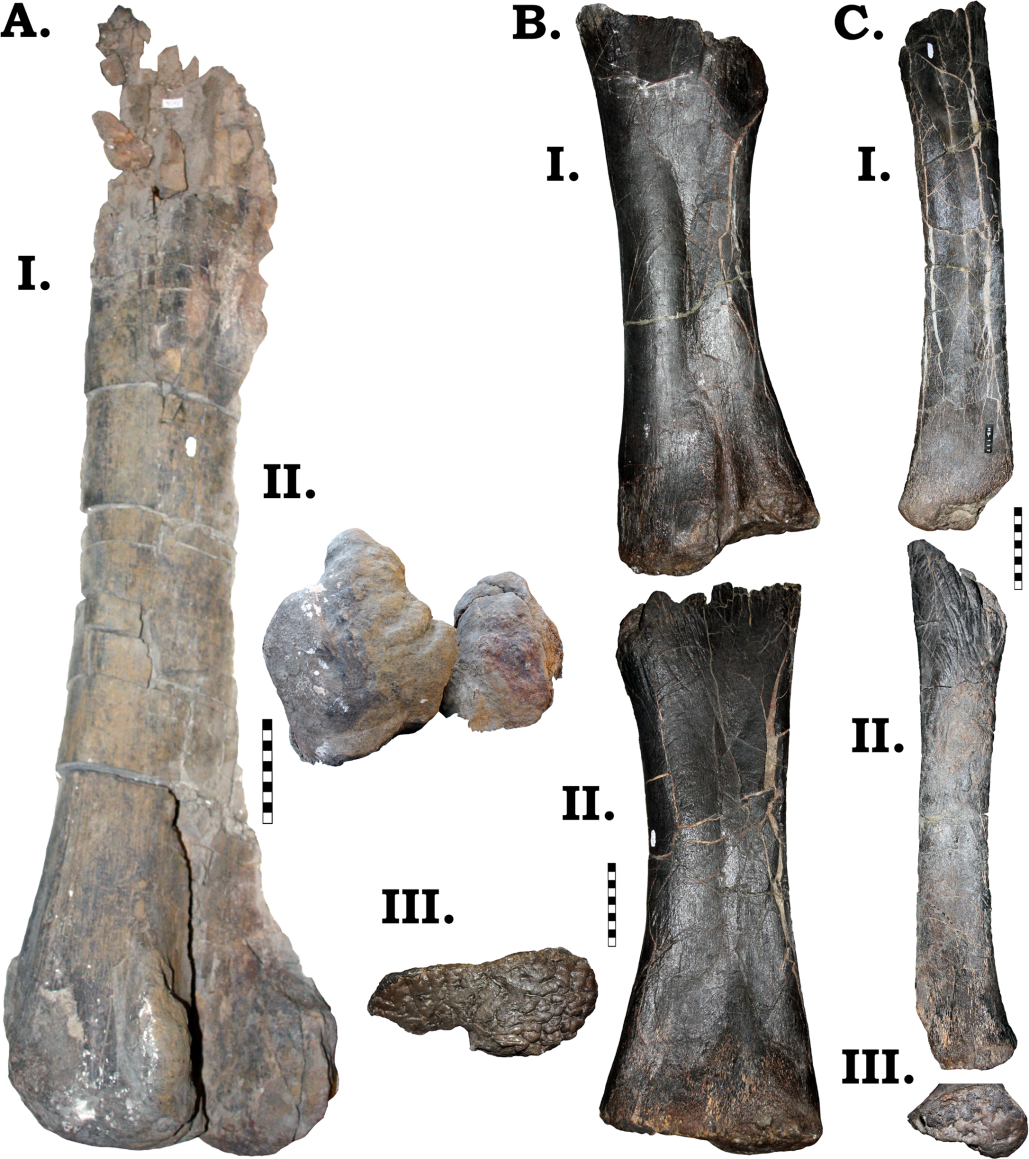
Primary hind limb elements of GPDM 220. A. Incomplete and unprepared left femur in I. posterolateral oblique view, and II. distal view. B. Incomplete right tibia in I. anterior, II. posterior, and III. distal views. C. Incomplete left fibula in I. anterior, II. posterior, and III. distal views. Elements not to scale with one another. Scale bar = 10 cm.

The only femur recovered from GPDM 220 is fragmentary and only constitutes approximately the distal two-thirds (Fig 28). At the time of our analysis, the femur had not be fully prepared, so our analysis could only examine it in oblique view from an opened field jacket. The proximal most portion is directly above the 4^th^ trochanter. This proximal region is highly fragmented and incomplete. Distally from the 4^th^ trochanter, the condition and consolidation of the femur improves. Given that the 4^th^ trochanter is a medially located landmark in reptile femora, we can identify this as the left femur. The overall morphology of the diaphysis appears slightly less robust (more “gracile”) than that of GMHN-VP 101. The diaphysis appears more laterally narrow, and the mid-section of the femur (removed in entirety for histologic analysis) is very circular in cross-section). As opposed to the more “gracile” diaphysis, the distal condyles are large and robust. The medial condyle is significantly more bulbous than the lateral condyle (anteroposteriorly, laterally, and dorsoventrally). The overall distal morphology is very similar to that of GMNH-PV 101. Further preparation of the element will be needed for detailed a discussion of its morphology.

#### Tibia

The tibia recovered from GPDM 220 in incomplete, and constitutes the approximately distal ¾ (Fig 28). While the proximal end is missing, the preservation and nature of the remaining bone is excellent, representing one of the best preserved elements from GPDM 220. Because there is some anteroposterior crushing of the diaphysis, the overall cross-sectional morphology of the diaphysis is strongly elliptical (as seen in AMNH 5761). Especially in posterior view, the distal most portion of the cnemial crest (as it starts to flare out from the diaphysis) can be observed. The diaphysis is very straight, and progressing distally, there is a gentle expansion finally terminating with the eroded distal condyles. In anterior view, along the distal end, there is a slight medioventral angle (~10°); this medioventral angle is observed in other *Camarasaurus* tibiae (such as GMNH-VP-101 and AMNH 5761) and allows us to identify this as the right tibia. In anterior view, the medial malleolus is the more robust of the two condyles (yet nowhere near as robust as the femoral condyles), and is slightly more distally and anteroposteriorly elongate. The distal articular surface of the tibia is completely intact, and especially in ventral view, one can see it laterally tapers to a strong point (as seen in AMNH 5761).

#### Fibula

As in the other two hind limb elements, the fibula of GPDM 220 is fragmentary (Fig 28). The fibula constitutes approximately the distal ¾. While the proximal end is missing, the distal portion is largely intact–save for some damage to the articular surface. The fibula of GPDM 220 compares favorably to that of GMNH-PV 101. The diaphysis is fairly straight–save for a slight medial curvature–and the cross-section would appear to be ovoid. Based on the more right lateral position of the tibial articulating medial crest, and the lateral malleolus, we identify this as the left fibula.

#### Metatarsal

This single metarsal of GPDM 220 is under 20 cm in proximodistal length (Fig 27). As in YPM 1910 and GMNH-PV 101, the proximal end is greatly widened laterally with a slight concavity, opposed to the narrower, transversely oriented distal end. Unfortunately, the distal end is damaged, so its precise morphology cannot be determined. However the articulating sulcus with the preceding phalanx is evident. The strong “triangular” symmetry of the proximal end–in comparison especially to YPM 1910 –suggests this is metatarsal IV.

### Histology

#### Femur core

**Fig 29:**
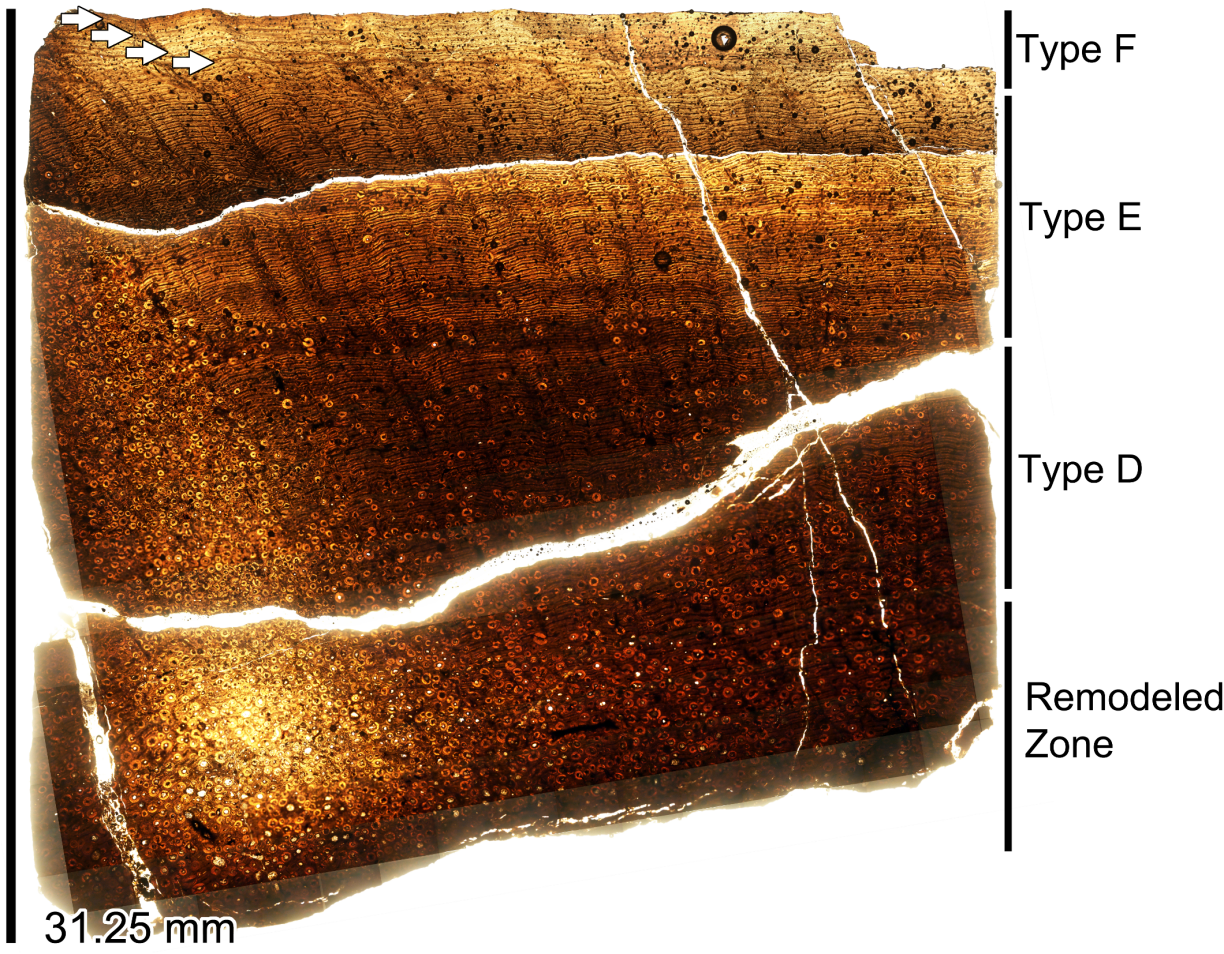
Histology of femur core from GPDM 220. The concentrations of tissue types D—F indicate a HOS of 10 out of 13. An EFS is present, and the LAGs constituting it are marked with white arrows.

A core section from the femur was removed following the coring methods of Stein and Sander [23]. The complete core section taken from GPDM 220 is 30.94 mm thick (Fig 29). There is a dramatic elongated zone of remodeling in the core section. The core was taken from the anterior face approximately mid-diaphysis, yet there we no externally visible processes or pathologies that could indicated such localized remodeling. Histologic descriptions are largely based on the lateral side of the core. Endosteally there is a zone of Haversian bone, while progressing periosteally, the cortex consists of less frequent remodeling. The majority of the cortex consists of Type D and E bone (see Klein and Sander [24] for bone tissue demarcations. Simply put, this method follows a 7-part alphabetical hierarchy denoting bone microstructure–bone Type A having no primary osteons, bone Type G consisting entirely of multi-generational secondary osteons). A few Lines of Arrested Growth (LAGs) appear to be present in the Type E zone, and the proportionally small Type F bone zone records 5 to 6 closely spaced LAGs that appear to be an External Fundamental System (EFS). GPDM 220 appears to represent Histologic Ontogenetic Stage (HOS) 10 out of 13 [24]. The calculated femur length of GPDM 220 (approximately 110 cm) is also consistent with femoral length versus HOS correlations found by Klein and Sander [24]. That the femur of GPDM 220 possesses an EFS and a cortex of non-Haversian bone, indicates that this animal was skeletally mature, but not extreme dotage compared to higher HOS specimens documented by Klein and Sander [24].

#### Dorsal rib

**Fig 30:**
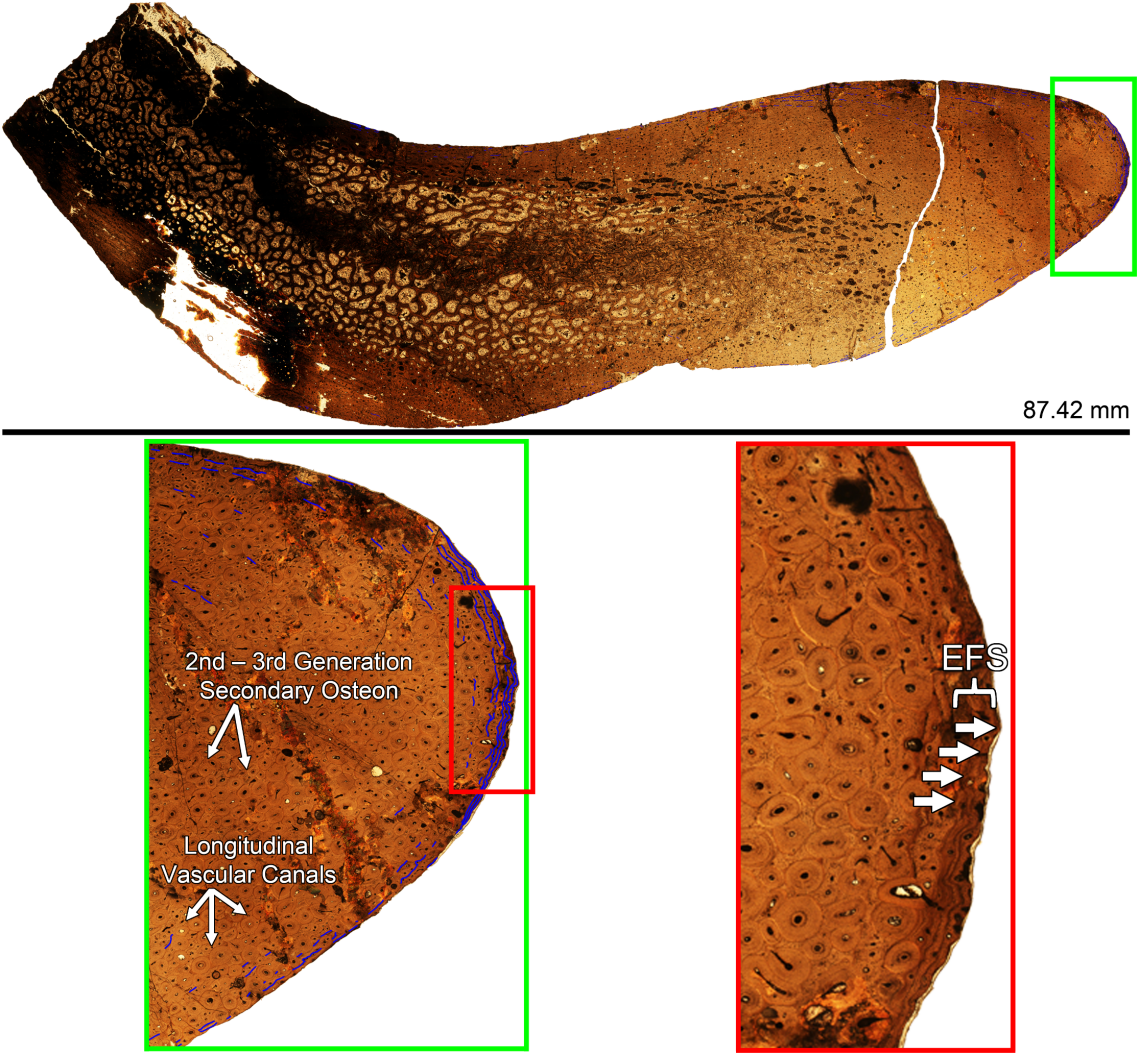
Histology of dorsal rib from GPDM 220. The green inset box showing greater detail of the histologic composition of this element–from the generations of secondary osteons, to vascular canal forms, to LAGs (outlined in blue). Red inset box is a higher magnification of the periosteal most portion of the outer cortex highlighting the four LAGs that constitute the EFS (marked by white arrows).

A transverse section from the right dorsal rib 4 was histologically sampled for age determinant histology (Fig 30). Upon initial observation, the mineralized tissue is highly vascularized with numerous resorption cavities. The anterior intercostal ridge is composed entirely of dense Haversian bone, however the medial portion of the rib records pertinent life history information. Laterally adjacent to the deep cortex trabecular bone, the remainder of the cortex is an approximately 2 cm zone of highly vascular, and predominantly fibrolamellar bone. The vascular canal orientation of the entire section is primarily longitudinal. The cortex of this dorsal rib is predominantly composed of secondary osteons. Note, however, that it is not Haversian bone–interstitial tissue in the form of woven primary bone is still evident though the cortex. In fact the deep cortex trabecular bone, the trabeculae themselves appear to be mainly composed of primary bone with little signs of remodeling. From the deep cortex trabecular bone–endosteally to periosteally–the number of generations of secondary osteons appears to be at most three, periosteally decreasing to two generations. Due to this patchwork remodeling, it is difficult to denote LAGs; yet several LAGs are discernable in the periosteal-most ~1 cm. In this zone, approximately ten LAGs can be observed. In this periostealmost ~1 cm, the tissue has up to two generations of secondary osteons. The periosteal-most portion exhibits an abrupt tissue change; this outermost zone is entirely composed of primary tissue with fewer and smaller diameter vascular canals. The canals are still predominantly longitudinal, but some reticular canals are present. Within this periosteal-most, less vascularized zone are three to four fairly evenly spaced LAGs which we identify as an EFS. Since an EFS is the histologic indicator of growth cessation, and in conjunction with the femur core, we hypothesize that GPDM 220 had reached skeletal maturity (in colloquial terms, analogous to an “adult”).

The collective tissue morphology of this dorsal rib indicates that GPDM 220 was a skeletally mature individual. As observed in the femur core, the presence of an EFS indicates that this animal was skeletally mature, yet the tissue morphology within the cortex (primary bone, generations of secondary osteons, etc.) suggest that this animal was not an extremely old individual. As observed within specimens of the hadrosaur *Maiasaura*, mature animals could possess an EFS, yet have little if any remodeling within the cortex [25]. Such tissue combinations reflect that the animal was growing rapidly, yet had only attained skeletal maturity for a short while before death [25]. Thus from the tissues observed in GPDM 220, we could hypothesize that the animal had lived for some time past skeletal maturity, but not long enough to result in complete cortex remodeling.

While only approximately ten LAGs can be observed in the outermost cortex, possible portions of earlier LAGs can be observed in the deeper cortex tissue. And while a minimum of ten LAGs are definitively observed, the location of these LAGs in conjunction with the tissue morphology lends support to the hypothesis that GPDM 220 was much older than 10 years old at its time of death. The retrocalculation method of Waskow and Sander [6] may be applicable in determining a more precise age for GPDM 220. Waskow and Sander’s [6] retrocalculation methodology requires making long axes on the scan image, and measuring the smallest and largest space between LAGs; with these spacing values, one then marks off the distance until reaching the origin. This retrocalculation method could be very useful for individuals with incomplete growth records; however, such a methodology implies uniform growth–and the record of sauropod ontogeny explicitly argues against such (albeit at least limbs appear more isometric). However, using a modified version of Waskow and Sander’s [6] retrocalculation method, we used the greatest LAG spacing to calculate a maximum age estimate. Using this methodology in combination with the LAG record, we calculate an estimated age of death of 35 years for GPDM 220 (note we believe this is the maximum age value, therefore the animal was in all likelihood younger, perhaps ~30).

## Discussion

### Postcranial markings

A number of the elements, particularly the limb elements, have numerous grooves and markings on the surfaces. These grooves are especially abundant on the partial tibia and fibula. With incomplete elements and such abundant surface damage, these marks were initially thought to be predation marks from theropod teeth (there were two shed theropod teeth recovered with GPDM 220, and this notion was incorporated into the display at the G.P.D.M.). While predation marks are well documented in the fossil record, such marks typically have order and structure [26]. The marks on GPDM 220 appear highly random–they typically appear to be fairly uniform in depth, and they frequently crisscross or intersect. Based on the uniform shallow depth and irregular nature of most of these marks, we believe these may be abrasion marks, perhaps due to moderate transport and that some of the deeper ones may possibly be tooth marks or more likely accidental prep damage.

### Cervical pathology

Regarding the cervical series of GPDM 220, perhaps one of the most striking features is the large pathology of the ventral surface of Cv6 (Fig 17). While histologic sampling nor computed tomography (CT) scanning was done on this structure to reveal its internal morphology or composition, it is clear that this is not a normal cervical feature. Due to the size and ventral nature of this pathology it almost certainly competed with space previously occupied by critical soft tissues (esophagus, trachea, etc.) and it would not be surprising if this structure had some effect on the animal’s quality of life. While it may make an entertaining story to say that this pathology ultimately caused the demise of GPDM 220, there is absolutely no evidence at this time to support such a claim.

**Fig 31:**
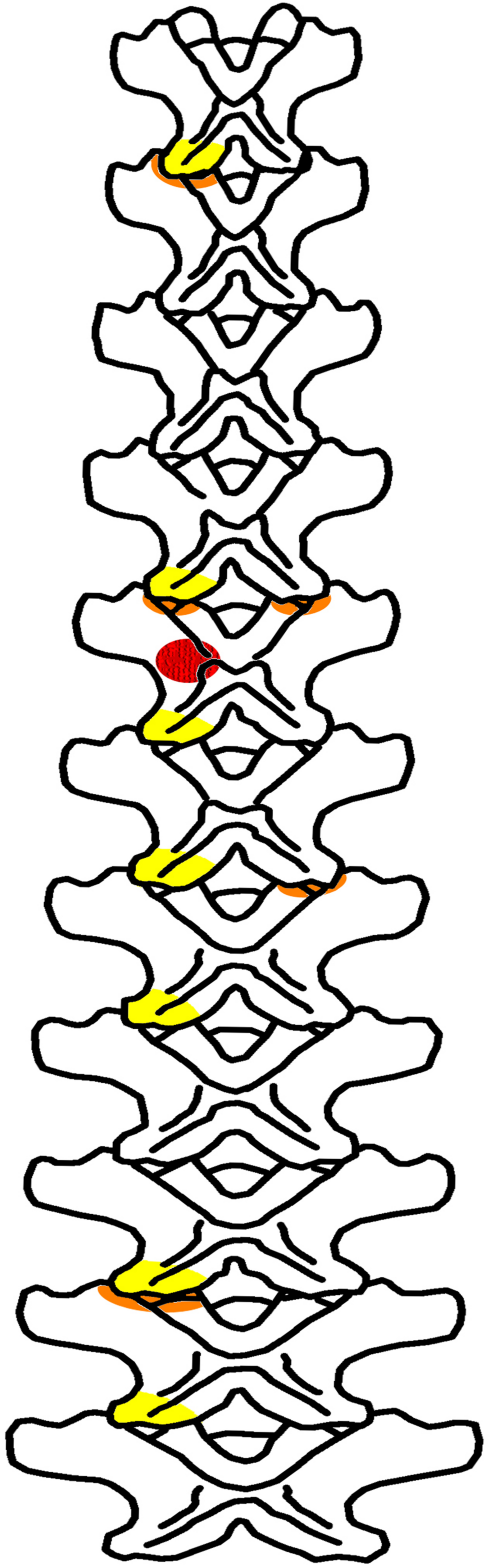
Schematic dorsal view of the cervical series of GPDM 220 highlighting the distribution of vertebral pathologies. The red circle indicates the large ventral pathology, while yellow indicates the postzygapophyseal foramina, and the orange indicates the prezygapophyseal foramina. Schematic drawing modified from Christopher [28].

However, there is a potential correlation to the unusual zygapophyseal foramina. While foramina in the centrum would be easily explainable as part of the pneumatic architecture, such features in the zygapophyses are more difficult to explain. Yet it must be stated that pneumatic structures can be present in the zygapophyses. Britt [27] demonstrated that pneumatic foramina did occur on the zygapophyseal articular surfaces of an ostrich and pterosaur ([27]; M. Wedel pers. comm. 2016). Since CT scanning was not conducted on the cervical series of GPDM 220, we cannot falsify that these zygapophyseal foramina are not pneumatic in origin, however, given the large pathology on Cv6, we suggest an alternative explanation for these structures. In reviewing the distribution of these foramina in the cervical series, the majority appear on the left side (Fig 31; [28]). This large pathology is situated on the ventral surface and towards the left lateral side of Cv6. Eight cervical vertebrae possess the foramina (Cv2, 3, 5, 6, 7, 9, 10, and 11)—seven of these foramina occur on the postzygapophyses, while five occur on the prezygapophyses; thus these features cannot be random osteological oddities. As reported in the tibia of a *Maiasaura*, Cubo et al. [29] documented secondary compensation growth associated with trauma. Likewise, in regards to cervid antlers, it has been documented that trauma can result in a plethora of abnormal growths [30]. In regards to cervical mobility, such an unevenly distributed weight (even one proportionally as small as this pathology) could result in torsional stresses. Such torsional loading would put stress on the left lateral side, and could induce an osteological response. These zygapophyseal foramina could be caused from compressional loading or the need for additional articular cartilage (extra articular “padding”).

For the time being, any causational hypothesis is speculation at best. The fact that the majority of these foramina occur of the same side, and the fact that in three vertebrae these foramina occur in articulating zygapophyses suggests a biomechanical function and origin. However, if this were the case, then why are these foramina sporadically distributed throughout the cervical series, and why is there the predominance of a postzygapophyseal location? Computer modelling to test for the effects of torsion could help to explain the location and distribution, but further analyses of the foramina themselves will be needed to verify any theories.

### Taxonomic assignment

#### Taxonomy

GPDM 220 possesses at least five autapomorphies of *Camarasaurus* as listed by Wilson [31]: lacrimal with long axis directed anterodorsally; groove passing anteroventrally from the surangular foramen to the ventral margin of the dentary; 12 cervical vertebrae (although one is missing or only partially preserved); anterior cervical neural spines bifid; and posterior cervical and anterior dorsal neural spines bifid (his *Camarasaurus* synapomorphies 1, 5, and 7–9). Wilson’s [30] quadratojugal characters (synapomorphies 2 and 3) may be present but these sutures in GPDM 220 are difficult to identify.

Within *Camarasaurus* there are currently four recognized valid species–*C*. *lentus*, *C*. *grandis*, *C*. *supremus*, and *C*. *lewisi*. Of these, *C*. *lentus* and *C*. *grandis* are most abundant and are preserved in approximately equal numbers (82% of *Camarasaurus* specimens are only identifiable at the genus level; 11.1% as *C*. *lentus* and 9.1% as *C*. *grandis*); *C*. *lewisi* and *C*. *supremus* are rare (1.5% and 4.5%). A significant number of *Camarasaurus* specimens have not been identified to species. *C*. *lentus* is distinguished from *C*. *grandis* on the basis of much wider neural arches in the anterior dorsal vertebrae and in having less transversely expanded dorsal ends of the neural spines in the anterior caudal vertebrae; *C*. *grandis* has narrow dorsal neural arches and significantly expanded caudal neural spines [3]. *C*. *supremus*, however, has wide neural arches similar to *C*. *lentus* but the expanded caudal neural spines like *C*. *grandis*. (The characters of *C*. *lewisi* will be discussed below.) GPDM 220 has narrow neural arches on the anterior dorsal vertebrae, similar to *C*. *grandis* [3], but the caudal neural spines are not preserved except for one possible fragment, which is relatively less expanded, although there is no way to determine if this is an anterior spine. The neural spine bifurcation of the cervical and dorsals of GPDM 220 is more like that of specimens assigned to *C*. *lewisi* (see below).

It is difficult to assess this mix of characters in GPDM 220, but the wide versus narrow neural arch conditions in species of *Camarasaurus* appear (when quantified as a ratio of neural arch width: centrum width in a number of specimens) to be extremes along a continuum of variation rather than clusters of discrete character states; similarly, variation is continuous when the relative width of the caudal neural spines is quantified (JRF unpublished data). Thus, the apparently diagnostic characters of *C*. *lentus* and *C*. *grandis* may be more nebulous than appreciated. Character states among species do not necessarily have to be numerically discrete in order to be valid, but with continuous variation it may be more difficult to reliably discern valid taxa.

That *C*. *supremus* and GPDM 220 have a mix of characters previously attributed to several species, and that the apparent continuous variation within characters attributed to the most common species, both suggest that the state of species assessments within *Camarasaurus* is somewhat uncertain. A specimen level phylogenetic analysis, as was conducted by Tschopp et al. [32] for diplodocids, would be an extremely laudable and fruitful endeavor to elucidate the nature of *Camarasaurus* taxonomy.

#### Ontogenetic development

In regards to growth and development, Ikejiri et al. [33] proposed the first ontogenetic trajectory for *Camarasaurus*. Based on morphologic characters (such as degree of pneumaticity, neurocentral fusion, and secondary osteological features), Ikejiri et al. [33] constructed a four-part ontogenetic trajectory ― Stage 1: juvenile–Stage 4: very old animal [33]. Considering the sheer wealth of material from this genus, *Camarasaurus* should represent our most understood North American sauropod, yet this is not the case–thus we laud the work of Ikejiri et al. [33] for beginning to understand the complicated life history of this unfortunately overlooked taxon. Similar to the Morphologic Ontogenetic Stage of Carballido and Sander [34], the methodology of Ikejiri et al. [33] relied solely on morphology (a common enough practice–see [5]). Unfortunately, more recent works on ontogenetic development are indicating that purely morphologic (or histologic) analyses do not reveal the full nature of ontogeny ([35, 16, 36, 37, 6, 38, 25, 39]). To fully understand the extreme complexities of ontogeny—especially in animals that undergo an order of magnitude size change through development—morphologic and histologic analyses must be performed. The analysis of Ikejiri et al. [33] serves as an impressive preliminary assessment into *Camarasaurus* ontogeny, yet without age determinant verification (i.e. histology), many of these “ontogenetic characters” could instead represent biomechanically linked characters.

Concerning *C*. *lewisi* (BYU 9047), we would further wonder why the secondarily proposed characters of McIntosh et al. (including degree of neural spine bifurcation, biomineralization of dorsal and sacral soft tissues, increased angle between the ilium and sacrum, and large zygapophyseal articular surfaces; [21]) were not ontogenetic as well. A histologic analysis of this specimen must be performed in order to definitively say anything about its ontogeny; yet until such is conducted, the defining characters of *C*. *lewisi* proposed by McIntosh et al. [21] could alternatively be attributes observed during late ontogeny. If this is true, then do skeletally mature individuals of *C*. *lentus* or *C*. *grandis* exhibit similar age determinant characters, and if so, how would we go about distinguishing these species? Alternatively, if these defining characters of *C*. *lewisi* merely represent an ontogenetic state, then *C*. *lewisi* would not represent a valid species, but instead an end member ontogimorph (i.e. the debated *Triceratops*–*Torosaurus*; [16]). Further and detailed analysis into this possibility must be conducted in order to determine such, yet the complex life histories of dinosaurs indicates that such a scenario is certainly not unprecedented.

**Fig 32:**
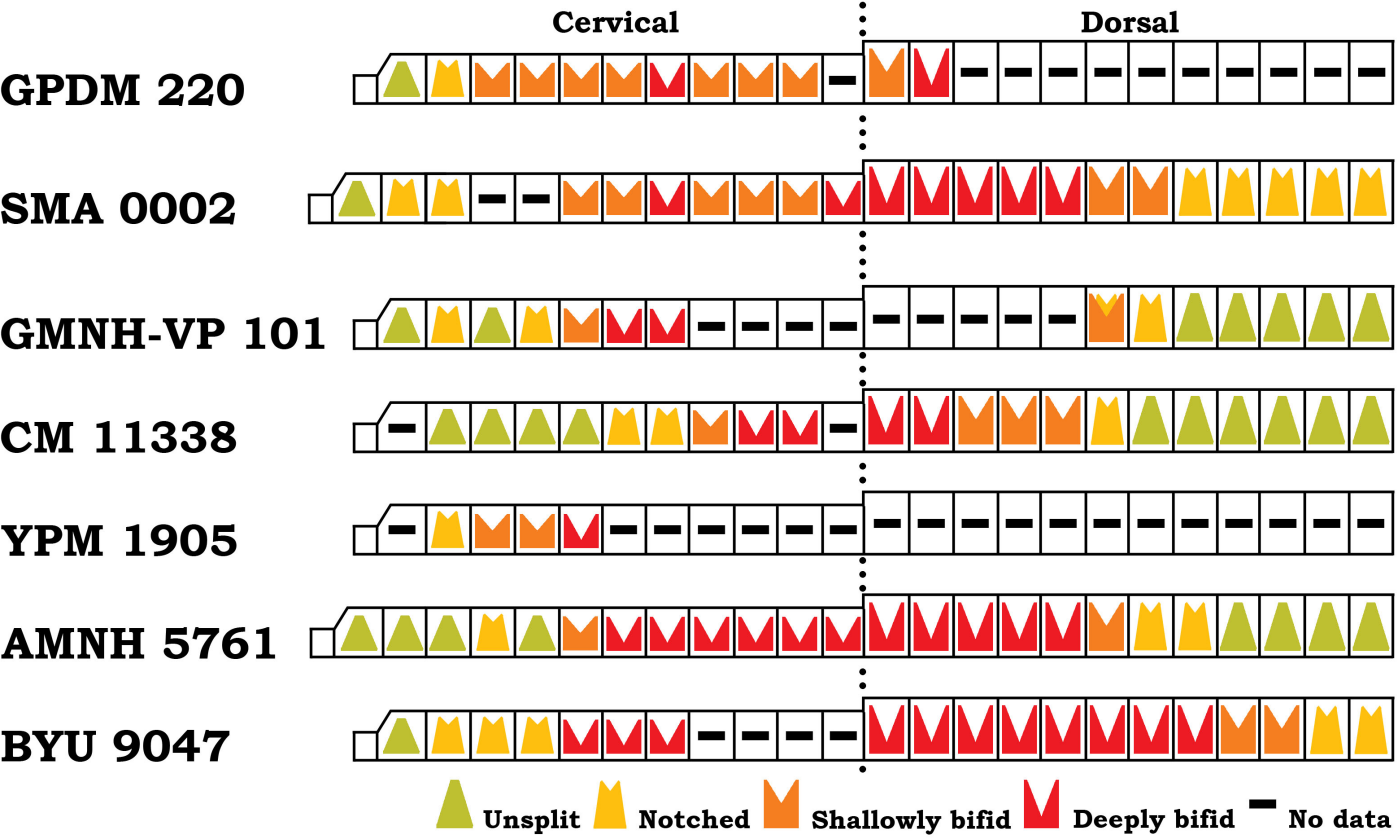
Schematic diagram of the degrees of neural spine bifurcation within *Camarasaurus* specimens. Modified from Wedel and Taylor [42]. Note that we alternatively recognize dorsal 6 of GMNH-PV 101 as “shallowly bifid”.

As Woodruff and Fowler [5] and Woodruff [40] have demonstrated, neural spine bifurcation is a complex character–it develops ontogenetically, but the degree of bifurcation can be controlled by body size. Ontogenetic vertebral allometry has likewise been documented within the spinal column of *Alligator mississippiensis* [41]. In analyses of diplodocids, neural spine bifurcation is absent or less developed in the smallest specimens, but as the animal increases in size (and the vertebral column increases in mass), there is biomechanically the need for the increasing degree in spine bifurcation. Because of this complex relationship between ontogeny and biomechanics, an immature animal with a large stature could initially appear to be more mature (expresses a greater degree of spine bifurcation) than an older individual with a small stature (expresses a lesser degree of spine bifurcation), which in turn could appear to be more immature (Fig 32; [42]). This was certainly the case for SMA 0002, and it was only because of the age determinant analysis of Waskow and Sander [6] that the maturational identity of this individual was possible.

The individual age, stature, and morphology of GPDM 220 may all initially appear counterintuitive—the overall size and some morphologic attribute (i.e. degree of spine bifurcation) suggests immaturity, while other morphologic attributes (i.e. neurocentral fusion) and the histology suggests maturity. In regards to the identification of GPDM 220, to remain conservative for the time being, we would alternatively propose that this “conflicting information”—apparent “immature” morphology and stature, and histology could be highlighting the complex relationship between biomechanics and ontogeny (furthering our understanding on the complexities of sauropodomorph skeletal plasticity; [35]). Therefore we tentatively propose that GPDM 220 is a histologically mature, small statured individual of *Camarasaurus* sp.

### Camarasaurid global distribution

The macronarian sub-clade Camarasauridae ([43]; and from here on referred to as non-titanosauriform macronarians) is definitively represented by the genus *Camarasaurus* with its four species–*C*. *lentus*, *C*. *grandis*, *C*. *supremus*, and *C*. *lewisi*. Besides the genus *Camarasaurus*–*Aragosaurus*, *Europasaurus*, *Galveosaurus*, *Tehuelchesaurus*, *Janenschia*, *Tastavinsaurus*, and *Euhelopus* are all recovered as non-titanosauriform macronarians [44]. Plotting the paleobiologic distribution, it would appear that non-titanosauriform macronarians have a Laurasian and Gondwanan presence from the Late Jurassic through the Early Cretaceous. However, among these proposed non-titanosauriform macronarians, there is disagreement regarding phylogeny. *Europasaurus* in other analyses is recovered as a member of Brachiosauridae [45, 46], *Galveosaurus* perhaps a part of the eusauropod clade Turiasauria [47, 44], *Janenschia* as a titanosaur (originally by Janensch [48]) to a sister-taxon to *Mamenchisaurus* [46], *Tastavinsaurus* as an andesauroid [46], and *Euhelopus* as a Euhelopodidea [45, 46], to a Somphospondyli [49]. Temporarily excluding these debated taxa, the non-titanosauriform macronarian genera appear to be *Aragosaurus*, *Tehuelchesaurus*, and *Tastavinsaurus*.

Based on the global and temporal distribution, it would appear that non-titanosauriform macronarians may have a Laurasian–and potentially North American–origin. As Foster and Peterson [50] noted the probability of Apatosaurinae being an endemic North American sauropod clade, the earliest and great diversity of non-titanosauriform macronarians is in North America. The earliest camarasaurid thus far identified is *Camarasaurus* from the Late Jurassic Morrison Formation (latest Oxfordian–early Tithonian) of North America (however, Moser et al. [51] claim to have fragmentary camarasaurid-like material from the Bajocian of India). Potentially at a co-occurring time is *Tehuelchesaurus* from the Late Jurassic (Oxfordian to Tithonian) Cañadón Calcáreo Formation [44] of Patagonia. Next is *Aragosaurus ischiaticus* from the Early Cretaceous (Valanginian–Hauterivian; [52]), and *Tastavinsaurus sanzi* from Early Cretaceous El Castellar Formation (Berriasian–Barremian), both from Spain. Furthermore, Sánchez-Hernández et al. [53] noted *Camarasaurus*-like teeth from the El Castellar Formation from Spain. And if Royo-Torres [54] is correct about the affinity and relationship of the derived branch Laurasiformes, then non-titanosauriform macronarians diversify and radiate during the Early Cretaceous across North America, Europe, and Asia.

### Camarasaurid distribution in North America

With its locality in the Little Snowy Mountains of Montana, GPDM 220 is the northernmost Morrison Formation sauropod recovered to date; and one of the northernmost Morrison dinosaurs (that honor belongs to the *Stegosaurus* GPDM 178). As mentioned in the Introduction, the cause of the sheer paucity of *Camarasaurus* remains from Montana could range from unintentional sampling biases or under-sampling to unfavorable ecosystems. At this time no explanation can be soundly proposed; yet regardless, GPDM 220 represents the first confirmed, described *Camarasaurus* material from Montana.

**Fig 33:**
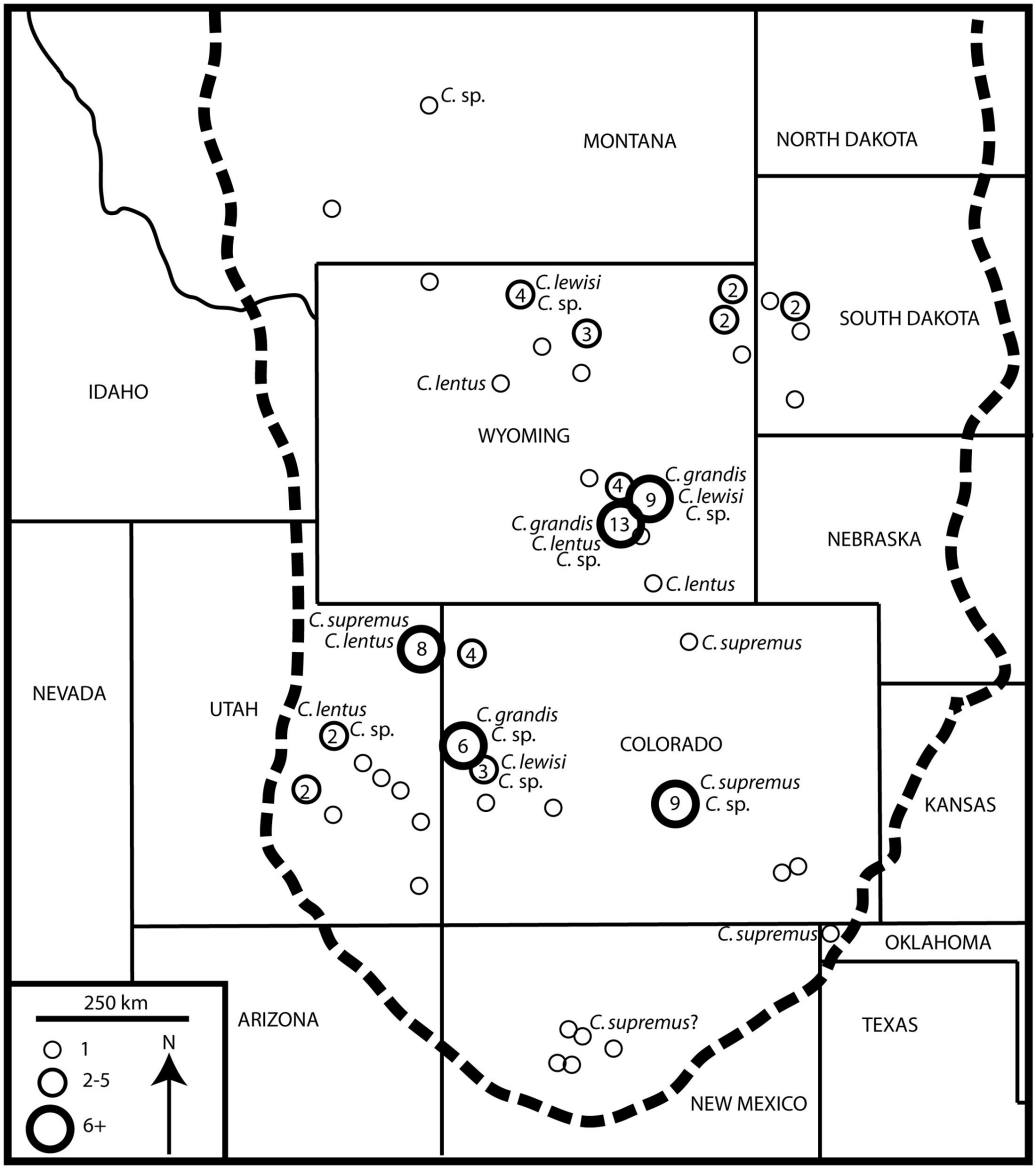
Geographic distribution of *Camarasaurus* specimens within the Morrison Formation by locality. Small open circles indicate single localities; numbers indicate numbers of quarries clustered in small areas represented by larger circles. N = 103. Dashed line is extent of Morrison Formation in outcrop and subsurface.

*Camarasaurus* is one of the best-known sauropod genera in the world, and it is the most abundant sauropod taxon in the Morrison Formation. It also appears to have had one of the highest population densities among large herbivorous dinosaurs during Morrison times [55]. *Camarasaurus* is represented in the Morrison Formation by more than 530 specimens, both isolated elements and partial to complete skeletons; approximately 83% of these specimens are identified as *Camarasaurus* sp. (73.7% by MNI). Among these 530 specimens are a minimum number of individuals of approximately 200 *Camarasaurus* representing adults to embryos (data updated from [1]). Out of this sample, there are approximately 50 partial, associated skeletons (ranging from nearly complete individuals to skulls or articulated series of vertebrae). These specimens are found at more than 100 localities throughout the outcrop area of the Morrison Formation (e.g., in every state in which the Morrison is exposed except Arizona; Fig 33) and apparently through most of its stratigraphic range from the upper Salt Wash Member into the upper Brushy Basin [56, 1, 3]. Unfortunately, stratigraphic correlation of Morrison Formation localities, and even within the Colorado Plateau, is not as reliable as we once thought it was [57], and sites at similar stratigraphic levels of the same member in the same part of the Plateau appear to be potentially of very different ages [58, 59]. This lack of stratigraphic control may undermine stratigraphic zonation of species of *Camarasaurus* [3].

**Fig 34:**
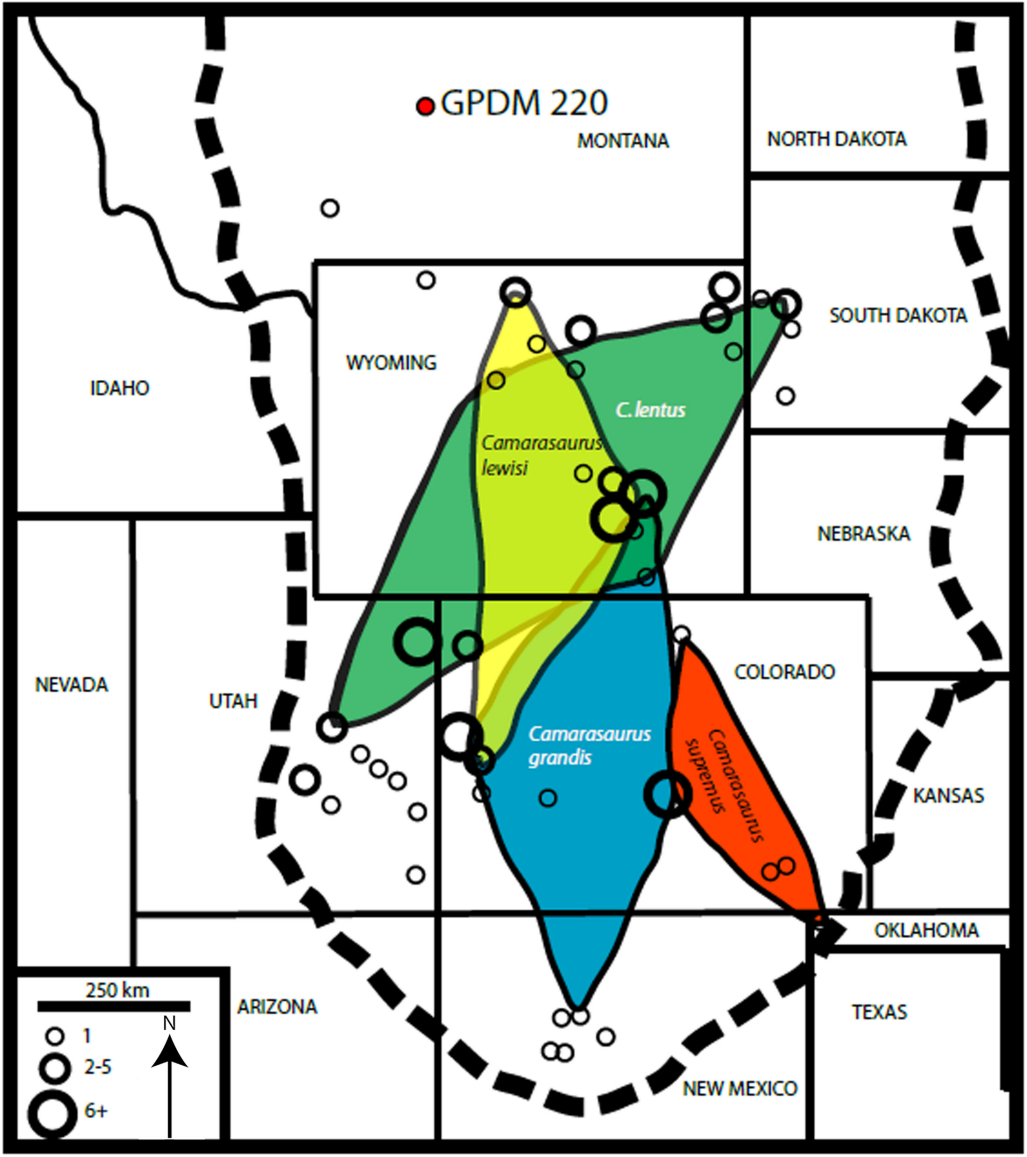
Known minimum geographic distributions of the four *Camarasaurus* species within the Morrison Formation.

With the assignment of new specimens to several of the species of *Camarasaurus*, the geographic distribution of species within the Morrison basin is slightly different than it was a decade ago (Fig 34; compare with [3] and [33]). The distribution is limited by a significant number of *C*. sp. occurrences, but *C*. *supremus* is still restricted to areas to the east (unless we include a handful of elements from the Dinosaur National Monument catalog identified as *C*. *supremus*). (We agree with Ikejiri’s, [3], reassignment of NMMNH P-21094, previously *C*. cf. *supremus* [60, 61], to *C*. *grandis*. This reassignment significantly changes the distribution patterns of those two species relative to what they would have been previous to 2005.) If a species level identification is ever possible, GPDM 220 would extend any species range far to the north. AMNH 625, from near Sturgis, South Dakota, may be referable to *C*. *lentus* by criteria of Ikejiri [3] based on its anterior dorsal vertebra, and this assignment would extend the range of this species far to the northeast. We have not included these range extensions here, pending additional study of these specimens. This pattern is difficult to interpret for significance, especially considering the lack of stratigraphic precision and the complexity and potential unreliability of species assignments, and thus the question of anagenesis or cladogenesis within the genus during Morrison times remains open.

### The *Camarasaurus* record from Montana

**Fig 35:**
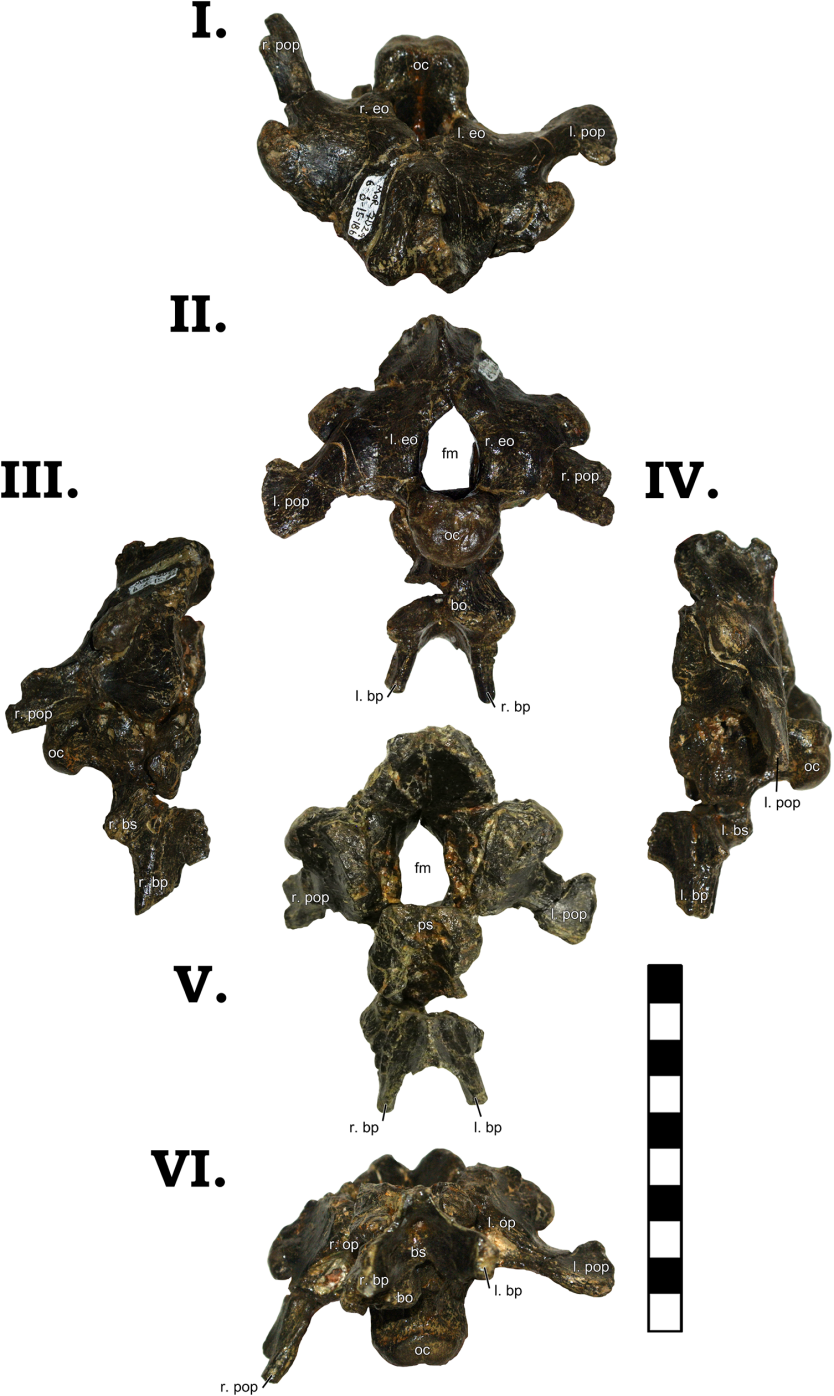
**The smallest thus identified *Camarasaurus* posterior cranial material (MOR 7029 6-6-15-186) in the six anatomical planes (dorsal [I], anterior [II], right [III] and left lateral [IV], posterior [V], and ventral [VI]).** All orientations to scale. Scale bar = 10 cm. Abbreviations: l. left, r. right, bo basioccipital, bp basipterygoid process, bs basiphenoid, eo exoccipital, fm foramen magnum, oc occipital condyle, op opisthotic, pop paraoccipital process.

*Camarasaurus* remains are remarkably sparse in Montana compared to their record in states to the south (e.g., Wyoming, South Dakota, Colorado, Utah, New Mexico). While the reason for this paucity of northernmost camarasaur material is the subject of speculation, we believe that it is simply a collecting bias due to less field activity in the Morrison in Montana compared to even northern Wyoming, where *Camarasaurus* is relatively abundant. The Black Hills and the flanks of the Big Horn Mountains have a number of sites that have produced sometimes nearly complete *Camarasaurus* specimens [62, 63, 64]. In addition to GPDM 220 and the unconfirmed identification of CM 1200 as *Camarasaurus* sp., a significant Morrison quarry in south-western Montana is producing remarkably complete Morrison taxa with copious cranial material, and one such specimen from this locality is the posterior portion of a small (presumably very immature) *Camarasaurus* skull (Fig 35). This posterior portion of the skull is 95.35 mm across at the paroccipital processes, compared to 123.45 mm in CM 11338. Thus, assuming a similar ontogenetic scaling, this Montana specimen is nearly a quarter smaller than CM 11338.

While we do not currently know the stratigraphic position of GPDM 220, the stratigraphic position of MOR 7029 6-6-15-186 is known. The quarry MOR 7029 6-6-15-186 comes from is approximately 15 meters above the underlying regressing Swift Formation and approximately 40 meters below the terrestrial and non-marine Kootenai Formation. Thus, based on relative stratigraphic boundary comparisons, the quarry would appear to be within the Lower Morrison–specifically Salt Wash equivalent. Matrix from this locality was sampled for sanidine and zircon crystals for radiometric dating (following the methodologies of Trujillo [57]); unfortunately analysis of these crystals resulted in a non-Morrison detrital date. In consideration of the regional paleogeography during this time, we would counter that this locality–and potentially much, if not all of the Morrison Formation in Montana–is temporally younger than the regional stratigraphy would appear to indicate. Throughout Morrison time, the epeiric Sundance Sea was progressively regressing northward. During the early Morrison, and initial regression of the seaway, the Colorado Plateau region would have been terrestrial, while Montana was fully marine. Thus regression, and terrestrial deposition, in the Montana region would have to have happened later. While depositional systems would and do certainly vary over such a latitudinal gradient, relative homology in these systems could be responsible for the “false positive” stratigraphic proximity. Rigorous testing is needed to substantiate this, but a new magnetostratigraphic analysis (currently underway by S. Maidment) throughout the Morrison Formation is potentially yielding supporting data for this hypothesis.

## Conclusions

Consisting of a complete skull, a nearly intact cervical series, and other fragments of associated post-crania, the specimen GPDM 220 represents the first *Camarasaurus* known from Montana. The remains of GPDM 220 also represent tentatively both the northernmost sauropod yet recovered from the Morrison Formation, and the northernmost sauropod remains in North America. With greater exploration of the Morrison Formation in Montana, we will undoubtedly discover more typical Morrison taxa, and likewise further verification that *Camarasaurus* is a part of the local sauropod fauna.

## Abbreviations

AMNH: American Museum of Natural History, New York, New York
BYU: Museum of Paleontology, Brigham Young University, Provo, Utah
CM: Carnegie Museum of Natural History, Pittsburgh, Pennsylvania
DNM: Dinosaur National Monument, Jensen, Utah
GPDM: Great Plains Dinosaur Museum, Malta, Montana
GMNH: Gunma Museum of Natural History, Japan
SDSM: Museum of Geology, South Dakota School of Mines and Technology, Rapid City, South Dakota
SMA: Sauriermuseum Aathal, Switzerland
UUVP: Natural History Museum of Utah, Salt Lake City, Utah
YPM: Yale Peabody Museum, New Haven, Connecticut
